# Supplementary material for: Implementing person-centred outcome measures (PCOMs) into routine palliative care: A protocol for a mixed-methods process evaluation of The RESOLVE PCOM Implementation Strategy
Source: BMJ Open. 2021 Sep 3;11(9):e051904. doi: 10.1136/bmjopen-2021-051904 (PMC8420722; doi:10.1136/bmjopen-2021-051904)
Supplement: Supplementary data [file bmjopen-2021-051904supp001.pdf]

Aspects of implementation									
TIDIER Checklist Components									
**Component Name**	**Why**	**What**	**Who**	**How**	**Where**	**When/How much**	**Tailoring**	**Modifications**	**How well**
**General implementation strategies**									
**Development of educational resources**	To enhance healthcare professionals' knowledge of what outcome measures are, their skills in using them, and how they may build practice around their use.	***Materials and procedures:***<br>Providing healthcare professionals with informational support to facilitate their understanding of what outcome measures are and how/why to use them. This is through the development of teaching and learning materials, including:<br><br>Pocket-guides, outcomes manual, instructional videos, online quizzes	Quality improvement facilitator (Martina), research nurse (Kath), PI (Fliss)	Print-based resources that can be used as an aide to memoir<br><br>Online resources (e.g., quizzes and instructional videos for each measure) available online through the RESOLVE website	Materials are for use during clinical practice and for reflection	Provided to all RESOLVE sites, with additional material being sent upon request	n/a. All sites have access to the same genenral educational resources	To complete retrospectively	To complete retrospectively
**Workshops and conference events**	Workshop and conference events aimed at responding to and discussing the common challenges that participating sites experience with regards to collecting, storing, and using outcome measures	***Materials and procedures:***<br>Providing informational support through teaching and guidance materials on outcome measures. This is through:<br><br>- Collective workshops and conference training events including representatives from all sites across the project	Entire RESOLVE team	Powerpoint presentations, collaborative group-based activities, question and answer sessions, and expert panel discussions.<br><br>Site representatives present at these events are encouraged to disseminate the knowledge and resources gained from attendance with their colleagues at site.	Conference/workshop events	Twice a year.	The content and agendas of workshop and conference events are derived from the common needs shared across sites participating in RESOLVE	To complete retrospectively	To complete retrospectively
**Site-specific implementation strategies**									
**Determining organisational needs**	Aimed at understanding the site-specific challenges to implementing PCOMs so that we can tailor our intervention according to each site's needs	A baseline assessment - developed through initial interview data and additional liaison with service leads at each site	Andy (research fellow)<br>Martina (QIF)	Interviews, email, telephone communications, site visits	All sites	At the start of tailoring our intervention for each site and revisited regularly throughout implementation	Tailoring intervention components to site-specific needs	To complete retrospectively	To complete retrospectively
**Formal training**	Enhance health care professionals' individual and collective understanding of, skills in using, and ability to build organisational practices around, PCOMs	***Materials and procedures:***<br>Providing participating sites with informational and organisational support through teaching and guidance on outcome measures. This is through:<br><br>- Site-based training sessions	Quality improvement facilitator (Martina), research nurse (Kath), PI (Fliss)	Working with sites to understand the challenges that they face to using outcome measures in practice. This information is then used to develop formal training sessions (e.g., presentations, case studies etc.) that help in addressing these issues. This is so that we can ensure that participating sites have the knowledge, understanding, and appropriate organisational resources in place to facilitate the consistent collection and use of outcome measures	At sites	Upon request from site	The nature and content of training sessions are tailored to each site's needs and priorities	To complete retrospectively	To complete retrospectively
**I.T. support**	To ensure local I.T. system readiness. That is, making sure that participating services have the analytic capacity and infrastructure in place so that they are able to input the appropriate data items into an electronic system and extract/ submit these to the RESOLVE team	***Materials and procedures:*** Informational, practical and technical support provided to data teams/analysts to assist them with inputting outcomes data into, and extracting it out of, their electronic system	Fliss (PI) and Assem (Data Analyst)	Practical and technical advice via face-to-face site visits, email and telephone	At sites/electronically	Upon request from site	Site specific support for:<br>- Inputting<br>- Viewing and using<br>- Extraction<br>- Submission of routine outcomes data to RESOLVE team	To complete retrospectively	To complete retrospectively
**Reporting and feedback**	Motivating and reinforcing the use of outcome measures through providing sites with meaningful feedback of their outcomes data. This can then be used to demonstrate how what they do as individuals/teams/organisations impacts patient care and outcomes	***Materials and procedures:***<br>Producing site based reports and graphs on outcomes data through our Registry protocol and feeding this back to each site.	Fliss (PI) and Assem (Data Analyst)	Electronic feedback via email	Electronic	Twice yearly	Site-specific support for:<br>- Producing outcomes reports<br>- Using outcomes data to inform practice at patinet, team, and service levels	To complete retrospectively	To complete retrospectively
**Running through general and site-specific implementation strategies**									
**Quality improvement facilitator (QIF)**	To champion the implementation of PCOMs at sites	Liase and work work sites locally in driving the implementation of PCOMs into practice, identifying and responding to local challenges/needs, keeping PCOMs on the agenda, and acting as a direct link between the research team and sites.	QIF (Martina)	Face-to-face support, email and telephone correspondence	At site	Throughout the RESOLVE implementation strategy	All support from the WIF will be tailored to local site needs and challenges	To complete retrospectively	To complete retrospectively
