# Supplementary material for: Implementing person-centred outcome measures (PCOMs) into routine palliative care: A protocol for a mixed-methods process evaluation of The RESOLVE PCOM Implementation Strategy
Source: BMJ Open. 2021 Sep 3;11(9):e051904. doi: 10.1136/bmjopen-2021-051904 (PMC8420722; doi:10.1136/bmjopen-2021-051904)
Supplement: Supplementary data [file bmjopen-2021-051904supp002.pdf]

Version 1.0 08/07/2019

Data item specification

Note: Whilst we plan on collecting data items on dates (e.g., date of referral, first contact data, date ready for care etc.), these will be pseudonymised in Excel by participating sites prior to being submitted to the Registry through the formula below.

Before pseudonymising dates, please make sure that there are no cases from before the date requested. For example, if data is requested from June onwards, disregard any cases with a starting episode date before June.

1. Excel handles dates numerically in the form of 'serial numbers', with each date being represented by a specific serial number. For example, the serial number for the date 24/10/2019 is '42667'. In pseudonymising dates, therefore, each site will use Excel to convert each date (dd/mm/yy) into its serial number through formatting cells into 'general' instead of 'date'.

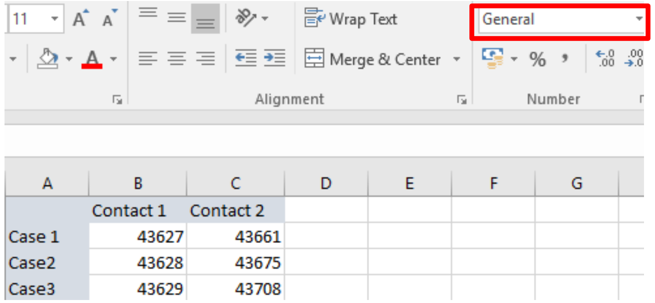

2. Hospices will then select and consistently use a random number between 1-500 (that the research team will not be made aware of) and add this to the serial number before submitting it to the registry.

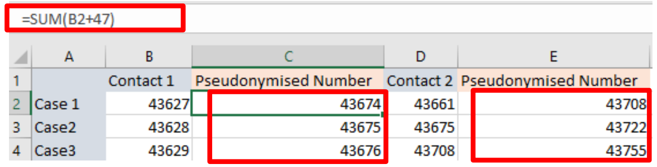

3. This will allow dates to be pseudonymised without the research team being able to trace back to the original date. The numbers that are produced can then be used to determine temporal factors of care that we are interested in(e.g., length of stay, time from referral to first contact) but without receiving dates which may identify participants.

Version 1.0 08/07/2019

Level 1: Patient Details – reported at patient level

No.	Variable Name	Definition	Format/Document	Use
1	Site ID	Unique identifier for each provider	A two letter and two digit code issued by the RESOLVE team (e.g., AA00)	To identify what palliative care provider is submitting data to the registry
2	Pseudonymised Unique Patient ID	A unique and pseudonymised patient identifier within the service's IT system that is used to identify the patient that is being cared by service providers.	This will be created by applying a macro to the NHS number, which provides a unique code which cannot be use to reverse to NHS number	To ensure the positive identification of a patient and that each individual's health records are associated with that individual and no other, while also enabling identification of repeat episodes of care for any one individual.
3	Age	The age of a patient at the start of their first episode of care	Age (in years old)	To determine the age of individual patients for conducting demographic analyses.
4	Gender	The gender of a patient	One of the following:<br>• Male<br>• Female<br>• Other<br>• Unknown	To determine gender of individual patients in a standardized format so that data can be used for related to service utilization, service needs, and epidemiological studies
5	Ethnicity	The ethnicity of the patient	One of the following:<br>• Mixed/multiple ethic groups<br>• Asian/Asian British<br>• Black/African/Caribbean/Black British<br>• Other ethnic group<br>• White	To determine ethnicity of individual patients in a standardized format so that data can be used for related to service utilization, service needs, and epidemiological studies
6	Primary Diagnosis	The primary life-limiting illness responsible for the patient receiving palliative care	Options showed in appendix 1 (ICD10 classification)	To determine a patients primary diagnosis.
7a	Secondary Diagnosis 1	Any secondary conditions / co-morbidities the patient has.	Options showed in appendix 1 (ICD10 classification)	To determine a patients secondary diagnosis.
7b	Secondary Diagnosis 2			
7c	Secondary Diagnosis 3			
8	Lives alone?	Whether or not the patient lives alone.	One of the following options:<br>• Yes	To understand a patients living arrangements for analysis (e.g., impact of living arrangements of cost of care and other outcomes)

Version 1.0 08/07/2019

No.	Variable Name	Definition	Format/Document	Use
			• No<br>• Unknown	
9	Usual Place of Residence	The patient's current main or permanent place of residence	One of the following:<br>• Patient's own home<br>• Other Private Residence (e.g., Relative's/Carers home)<br>• Care Home (residential/nursing facility)<br>• Hospice (inpatient specialist palliative care)<br>• Hospital<br>• Homeless<br>• Traveler<br>• Other	To understand place of care
10	Marital Status	A patient's marital status.	One of the following options:<br><br>• Single<br>• Married/Civil Partner/Cohabiting<br>• Divorced/Person whose Civil Partnership has been dissolved<br>• Widowed/Surviving Civil Partner<br>• Separated<br>• Not disclosed<br>• Unknown	To understand a patients marital status for analysis

Version 1.0 08/07/2019

Level 2: Episode Details – reported at episode level

No.	Variable Name	Definition	Format/Document	Use
11	Palliative episode number	The number episode that a patient is in	The corresponding number with a patient's episode (e.g., 1 for their first episode of care, 2 for their second, 3 for their third etc.)	To identify the number episodes a patient has experienced.
12	Referral Date	The date that a service receives a referral to provide palliative care for a patient for this episode	Date of referral (DD/MM/YYYY)	This will serve as a **key benchmarking variable**; the measurement of the time between referral and other variables (e.g., first contact date, date ready for care, episode start date). ***This date will be pseudonymised using the formula outlined at the start of this document.**
13	Primary Reason for referral	The primary reason for referral to specialist palliative care, as identified by person who refers patient.	One of the following options:<br>• Pain<br>• Other symptom control<br>• Emotional/psychological/spiritual support (patient)<br>• Emotional/psychological/spiritual support (family)<br>• Social/financial support (patient)<br>• Social/financial support (family)<br>• Rehabilitation<br>• Discharge planning<br>• Care in last days of life<br>• Respite<br>• Other	For analyses regarding why patients are referred to specialist palliative care services
14	Referral Source	The facility/organization which the patient was referred from for the start of a particular episode.	One of the following:<br>• Hospital – NHS<br>• Hospital – Outpatient<br>• Hospice – Inpatient<br>• Hospice – Day services<br>• Hospice – Outpatient<br>• Community – GP<br>• Community – Hospice at home<br>• Community – Out of hours services	To assist in understanding referral patterns and service planning.

Version 1.0 08/07/2019

No.	Variable Name	Definition	Format/Document	Use
			• Community – District/Community matron<br>• Self-referral/family-referral<br>• Internal referral within organisation<br>• Other (please state)	
15	First Contact Date	The date the clinical team makes contact with patient to assess needs after referral	Date of first clinical contact (DD/MM/YYYY)	This is a **key benchmarking variable** as it can be used to measure response time. ***This date will be pseudonymised using the formula outlined at the start of this document.**
16	Date Ready for Care	The date the patient is ready and available to receive palliative care. This may be determined through referral or first contact with patient	Date identified that patient is ready to receive palliative care (DD/MM/YYYY)	**Key benchmarking variable** as it is used to identify and account for early referrals (i.e., when a referral is received by a patient is not ready for care). ***This date will be pseudonymised using the formula outlined at the start of this document.**
17	Episode Start Date	The date of the first face-to-face palliative care assessment, using the set of core outcomes listed above. This is when clinical care starts	The date when clinical care starts (DD/MM/YYYY)	This date (along with episode end date) will be used to determine the length of stay for each episode of care. ***This date will be pseudonymised using the formula outlined at the start of this document.**
18	Care Setting	The setting/location where a patient is overnight.	One of the following:<br>• Hospital – Inpatient<br>• Hospital – Outpatient<br>• Hospice – Inpatient<br>• Community – Outpatient<br>• Community - Home visiting<br>• Community – Day care or day service<br>• Community – Nursing/residential home<br>• Community – Patient's/Carer's own home<br>• Community – Community hospital<br>• Other (please state)	To determine where an episode of care takes place
19	Team delivering care	The team delivering care within setting	Provided by the reporting organisation	Each organisation has different 'labels' for their teams (such as different clinics, therapy teams etc), yet may require sub-analysis by team to support effective quality improvement – we will therefore allow each reporting organisation to self-specify this variable
20	Episode end date	The date when a patient moves from one setting of care to another, dies, or	Date of episode end (DD/MM/YYYY)	To use with episode start date to determine the length of care for each episode. ***This date will be pseudonymised using the formula outlined at the**

Version 1.0 08/07/2019

No.	Variable Name	Definition	Format/Document	Use
		is no longer receiving palliative care		**start of this document.**
21	Episode result	The reason that an episode of palliative care ended	One of the following:<br>• Discharged to own home (or relatives/carers home)<br>• Discharged to nursing/residential home<br>• Discharged to hospital<br>• Discharged to NHS hospice<br>• Discharged to non-NHS hospice<br>• Died<br>• Unknown	Describes how the episode ended, determines number of deaths, and discharge locations.
22	Preferred place of death (if relevant)		One of the following:<br>• Nursing/residential home<br>• Home<br>• Hospice<br>• Hospital<br>• Other	
23	Whether patient is now deceased		• Yes<br>• No<br>• Unknown	Reporting may occur some time after episodes of care are completed. Knowing if the patient subsequently died will enable reporting of how early in the trajectory of illness patients are being seen.
24	Date of death		Date of death (DD/MM/YYYY)	This date will be used to determine at what point in the illness trajectory, the episode of care was delivered. ***This date will be pseudonymised using the formula outlined at the start of this document.**
25	Place of death (if relevant)		One of the following:<br>• Nursing/residential home<br>• Home<br>• Hospice<br>• Hospital<br>• Other	

Version 1.0 08/07/2019

Level 3: Phase Details – reported at contact level

No.	Variable Name	Definition	Format/Document	Use
26	Palliative Phase Number	The number phase that a patient has within an episode.	The corresponding number with a patient's phase (e.g., 1 for their first phase of illness, 2 for their second, 3 for their third etc.)	To identify the number phases a patient has experienced within an episode and to work out number of patient contacts.
27	Palliative Phase of Illness	Stage of a patient's illness, assessed at the start of the phase as assessed by Phase of Illness questionnaire (see appendix X)	One of the following:<br>• Stable<br>• Unstable<br>• Deteriorating<br>• Dying<br>• Deceased<br>• Bereavement/post death support<br>• Unknown (see appendix X for phase definitions)	To identify the stage of a patient's illness
28	Phase Start Date	The date each new phase of illness begins	Phase start date (DD/MM/YYYY)	When used with phase end date, can tell us the length of a phase of illness. ***This date will be pseudonymised using the formula outlined at the start of this document.**
29	Phase End Date	The date each phase of illness ends	Phase end date (DD/MM/YYYY)	When used with phase start date, can tell us the length of a phase of illness. ***This date will be pseudonymised using the formula outlined at the start of this document.**
30	Is this the End of a Phase?	Whether the patient has reached the end of a phase of illness.	One of the following:<br>• Yes<br>• No	To clarify the end of a phase of illness
31	Has the Phase Changed?	To highlight whether a patient has undergone a change in their phase of illness.	One of the following:<br>• Start Phase<br>• Continuing Phase<br>• End Phase	To cross check with phase of illness assessments
32	Contact Date	The date a health-care professional has clinical contact with patient.	DD/MM/YYYY.	To align contact dates – and clinical data collected at these - with changes in episodes and phases. ***This date will be pseudonymised using the formula outlined at the start of this document.**
33	Australia-Modified Karnofsky Performance	An assessment a patient's functional status at the beginning of a phase of illness	Functional status, as measured by AKPS ranging from 0-100% in 10% increments, (where 100% is fully functioning and 10% is unarousable). See	**Key benchmarking variable**. An analysis of functional status at phase start

Version 1.0 08/07/2019

No.	Variable Name	Definition	Format/Document	Use
	Scale (AKPS) Score		appendix X for scale	
34	Integrated Patient Outcomes Scale (IPOS) at	An assessment of a patient's symptoms and other concerns at phase start (see items between numbers X-X)	Inputting data from each item of IPOS (see appendix X)	**A key benchmarking variable**. A measure of a patient's perceptions of their symptoms at the start of a phase of illness
35	Barthel Activities of Daily Living	To assess patient's activities of daily living at phase start	Inputting data from each item of BARTHEL (see appendix X)	
36	Views on Care (VOC)	To assess a patient's ratings of their quality of life, overall well-being, and their view of the impact of the service on their problem(s) at the start of a phase of illness	Inputting data from each item of VOC (see appendix X)	To measure a patients views on care

Version 1.0 08/07/2019

Appendix 1: ICD10 Classifications

No.	RESOLVE Diagnoses Groups
1	Cancer - Cancer of bone, skin, mesothelial and soft tissue, thyroid or endocrine [C40-C49 & C73-C75]
2	Cancer - Cancer of breast [C50]
3	Cancer - Cancer of independent multiple sites [C97]
4	Cancer - Cancer of liver, intrahepatic bile ducts, gallbladder [C22-C24]
5	Cancer - Cancer of respiratory & intrathoracic organs, including lung (C30-C39)
6	Cancer - Cancer of the digestive organs, including colon, rectum, stomach, excluding liver, GB, pancreas [C15-C21 & C26]
7	Cancer - Cancer of the eye, brain and other CNS [C69-C72]
8	Cancer - Cancer of the female genital organs [C51-58]
9	Cancer - Cancer of the lip, oral cavity & pharynx [C00-C14]
10	Cancer - Cancer of the male genital organs, including prostate [C60-C63]
11	Cancer - Cancer of the pancreas [C25]
12	Cancer - Cancer of the urinary tract [C64-C68]
13	Cancer - Cancer of unknown primary or other unspecified [C76-C80]
14	Cancer - Lymphoid & haematopoietic cancer [C81-C96]
15	Multiple non-cancer conditions - addition to help with multi-morbidity
16	Non-cancer - All other non-cancer diagnoses
17	Non-cancer - Chronic renal failure [N18]
18	Non-cancer - Chronic respiratory disease [J40-J70]
19	Non-cancer - Dementia including Alzheimer's disease [G30 & F00-F03]
20	Non-cancer - Diabetes mellitus [E10-E14]
21	Non-cancer - Heart failure [I50]
22	Non-cancer - HIV disease/AIDS [B20-B24]
23	Non-cancer - Liver failure, chronic liver disease, other non-malignant liver disease [K70-K77]
24	Non-cancer - Motor neurone disease [G12]
25	Non-cancer - Neurological conditions (excluding MND and Alzheimer's) [G00-G99

No.	RESOLVE Diagnoses Groups
	excluding G12 & G30]
26	Non-cancer - Other heart & circulatory conditions excluding heart failure and stroke [I00-I99 excluding I50, I61, I63]
27	Non-cancer - Stroke, infarction or haemorrhagic [I61 & I63]
28	Unknown
