# Supplementary material for: Implementing person-centred outcome measures (PCOMs) into routine palliative care: A protocol for a mixed-methods process evaluation of The RESOLVE PCOM Implementation Strategy
Source: BMJ Open. 2021 Sep 3;11(9):e051904. doi: 10.1136/bmjopen-2021-051904 (PMC8420722; doi:10.1136/bmjopen-2021-051904)
Supplement: Supplementary data [file bmjopen-2021-051904supp003.pdf]

Version 1 (15/12/2020)

IMPLEMENTING PERSON-CENTRED OUTCOMES MEASURES INTO PALLIATIVE CARE

Information about this survey

What is this survey about?

This survey is part of a study called 'RESOLVE'. RESOLVE is a research programme aimed at facilitating the implementation of Person-Centred Outcome Measures into routine palliative care. This is so that, as a palliative care community, we ensure regular assessment and monitoring of symptoms and other concerns of those receiving palliative care. As part of our project we have developed an intervention strategy, and this survey is one way in which we are trying to understand if it is effective at facilitating the integration of outcome measures into everyday practice. More information on the RESOLVE project can be found through our website: https://www.hyms.ac.uk/research/research-centres-and-groups/wolfson/resolve.

Why have I been chosen?

You have been selected to complete this survey as you are a staff member at a RESOLVE-affiliated site that has received the RESOLVE implementation strategy. Sites with the best completion rate will receive a voucher for a textbook or one-year journal subscription.

Do you consent to taking part in this survey?

☐ Yes ☐ No

What about my data?

By completing and submitting this survey you are providing consent for your responses to be used as part of the RESOLVE research project. All data is anonymised and confidential and will be stored in accordance with General Data Protection Regulation (GDPR). If you have any questions, please contact the RESOLVE team (resolve@hyms.ac.uk).

Survey Instructions

This survey is split into 3 parts and should take no longer than 15 minutes to complete. Part A asks some brief questions about yourself and your role. Part B includes questions about what outcome measures you use and how you use them to inform your everyday practice. Part C asks more detailed questions about PCOMs.

Please take the time to decide which answer **best suits your experience for each statement and tick the appropriate box**

Part A: About yourself

- What is your professional role?
  ☐Nurse ☐Doctor ☐Allied health professional ☐Healthcare assistant ☐Chief exec/manager ☐ I.T. or data ☐Other (dropdown box)

- What is the name of your organisation?
  ☐ Dove House Hospice ☐ Marie Curie Bradford Hospice ☐ Wheatfields Hospice ☐ St Gemma's Hospice ☐ St Andrew's Hospice
  ☐ Grimsby Care Plus Group ☐ Kirkwood Hospice ☐ St Luke's Hospice ☐ St Catherine's Hospice
  ☐ City Health Care Partnership Hull ☐ Bradford District Care Trust ☐ York Teaching Hospital NHS Foundation Trust

- What is your age?
  ☐ 18-24 ☐ 25-34 ☐ 35-44 ☐ 45-54 ☐ 55+ ☐ Prefer not to say

- What is your gender?
  ☐ Male ☐ Female ☐ Other ☐ Prefer not to say

- By which ethnicity do you identify?
  ☐ White ☐ Black/African/Caribbean/Black British ☐ Asian/Asian British ☐ Mixed/multiple ethic groups ☐ Other
  ☐ Prefer not to say

- What setting do you work in (tick multiple if you work across settings)?
  ☐ In-patient hospice ☐ Outpatient/day therapy ☐ Community ☐ Hospital

- How long have you worked in palliative care?
  ☐ Less than a year ☐ 1-5 years ☐ 6-10 years ☐ 11-15 years ☐ 15+ years

- From the statements below, please choose an option that best describes ***your main role*** in relation to outcome measures:
  ☐ I am involved in managing or overseeing the implementation and use of outcome measures
  ☐ I am involved in using outcome measures as part of my everyday practice
  ☐ I use outcome measures occasionally as part of my everyday practice
  ☐ I am not using outcome measures as part of my everyday practice

Part B: What outcome measures do you use?

Outcome measures are short questionnaires that provide healthcare professionals with information on a person's own perception of their health status and well-being. They include palliative Phase of Illness, Integrated Palliative care Outcome Scale (IPOS), and Australia-modified Karnofsky Performance Scale (AKPS). This survey relates to your use of these outcome measures.

- Do you use the following outcome measures?

	At 1st assessment	At follow-up
palliative Phase of Illness	☐ Yes ☐ No	☐ Daily ☐ Weekly ☐ At clinical assessment<br>☐ Other (open free text response box)

	At 1st assessment	At follow-up
Integrated Palliative care Outcome Scale (IPOS)	☐ Yes ☐ No	☐ Yes ☐ No
Australia-modified Karnofsky Performance Scale (AKPS)	☐ Yes ☐ No	☐ Yes ☐ No

- At what follow up interval do you collect IPOS and AKPS data?
  ☐ At change of palliative Phase of Illness ☐ End of episode of care ☐ Other (open free text response box)

- Do you or your team use these measures to shape the care of individual patients, including in multi-disciplinary team meetings?

- Do you or your team use these measures to shape the services that your team or organisation delivers?

Part C: Detailed questions about outcome measures

These questions are designed to help us find out more about whether people and teams understand what outcome measures are and how they may be used during practice. For each statement please select an answer that best suits your experience.

**Section C1**	Strongly agree	Agree	Neither agree nor disagree	Disagree	Strongly disagree	Not relevant to my role	Don't know
**I can see how outcome measures differs from usual ways of working**	☐	☐	☐	☐	☐	☐	☐
**Palliative care staff in this organisation have a shared understanding of the purpose of outcome measures**	☐	☐	☐	☐	☐	☐	☐
**I understand how using outcome measures affects the nature of my own work**	☐	☐	☐	☐	☐	☐	☐
**I can see the potential value of outcome measures**	☐	☐	☐	☐	☐	☐	☐

- Please feel free to expand on your responses below

These questions are designed to help us find out more about how everyday practice is built around using outcome measures and how you think they relate to your role. For each statement please select an answer that best suits your experience.

**Section C2**	Strongly agree	Agree	Neither agree nor disagree	Disagree	Strongly disagree	Not relevant to my role	Don't know
**There are key people who drive the use of outcome measures and get others involved**	☐	☐	☐	☐	☐	☐	☐
**I believe that using outcome measures is a legitimate part of my role**	☐	☐	☐	☐	☐	☐	☐
**I am open to working with colleagues in new ways to use outcome measures**	☐	☐	☐	☐	☐	☐	☐
**I will continue to support the use of outcome measures**	☐	☐	☐	☐	☐	☐	☐

- Please feel free to expand on your responses below

These questions are designed to help us better understand the ways in which you experience using outcome measures as part of your role. For each statement please select an answer that best suits your experience.

**Section C3**	Strongly agree	Agree	Neither agree nor disagree	Disagree	Strongly disagree	Not relevant to my role	Don't know
**I can easily integrate outcome measures into existing work**	☐	☐	☐	☐	☐	☐	☐
**Outcome measures disrupt working relationships**	☐	☐	☐	☐	☐	☐	☐
**I have confidence in other people's ability to use outcome measures**	☐	☐	☐	☐	☐	☐	☐
**Work is assigned to those with skills appropriate to using outcome measures**	☐	☐	☐	☐	☐	☐	☐
**Sufficient training is provided to enable palliative care staff to implement and use outcome measures**	☐	☐	☐	☐	☐	☐	☐
**Sufficient resources are available to support outcome measures use**	☐	☐	☐	☐	☐	☐	☐
**Management adequately supports the use of outcome measures**	☐	☐	☐	☐	☐	☐	☐

- Please feel free to expand on your responses below

These questions are designed to help us understand the ways in you appraise the value of outcome measures. For each statement please select an answer that best suits your experience.

**Section C4**	Strongly agree	Agree	Neither agree nor disagree	Disagree	Strongly disagree	Not relevant to my role	Don't know
**I am aware of reports about the effects of outcome measures on patient care**	☐	☐	☐	☐	☐	☐	☐
**The palliative care staff agree that using outcome measures is worthwhile**	☐	☐	☐	☐	☐	☐	☐
**I value the effects that outcome measures have had on my work**	☐	☐	☐	☐	☐	☐	☐
**Feedback about outcome measure data can be used to improve how they are used in future**	☐	☐	☐	☐	☐	☐	☐
**I can modify how I work with outcome measures**	☐	☐	☐	☐	☐	☐	☐

- Please feel free to expand on your responses below

- Are you happy to be contacted to complete this survey again (in around 8-12 months' time)? This is so that we are able to explore the impact of the RESOLVE implementation strategy over time.

  ☐ Yes (if selected then include dropdown box that asks for email address so that we are able to contact, alongside statement that we will send them a certificate of participation for this survey)

  ☐ No (if selected then include dropdown box that asks if respondent wants to receive a certification of participation. If they do, prompt for email address)

Thank you very much for completing this survey.

What happens next?

This data will be stored confidentially and analysed as part of the RESOLVE project to understand the ways in which the RESOLVE implementation strategy has (or has not) worked to help implement outcome measures into routine clinical practice. This data will be used as part of reports, publications, and conference presentation. All information included within these outputs will be anonymised and it will not be possible for others to identify you. If you have any questions, please contact the RESOLVE team (resolve@hyms.ac.uk).
