# Supplementary material for: Implementing person-centred outcome measures (PCOMs) into routine palliative care: A protocol for a mixed-methods process evaluation of The RESOLVE PCOM Implementation Strategy
Source: BMJ Open. 2021 Sep 3;11(9):e051904. doi: 10.1136/bmjopen-2021-051904 (PMC8420722; doi:10.1136/bmjopen-2021-051904)
Supplement: Supplementary data [file bmjopen-2021-051904supp004.pdf]

Version 1 (15/12/2020)

Introduction to the Interview

Thank you very much for volunteering to be interviewed for the RESOLVE project. The aim of this project is to implement Person-Centred Outcome Measures (PCOMs) into routine palliative care across Yorkshire.

As part of RESOLVE, we have developed an implementation strategy to facilitate the implementation of PCOMs into practice. This strategy was developed based on what health professionals' thought were important challenges that needed to be addressed in order to integrate PCOMs into routine practice. Our strategy includes:

- Educational resources
- Workshop and conference events
- Determining organisational and team needs
- Formal and informal training
- IT support
- Reporting and feedback
- Quality improvement facilitator

The purpose of this discussion is to understand your thoughts and feelings on the extent to which you think this implementation strategy has been effective at enhancing the uptake and routine use of PCOMs in everyday practice. It should take around 30 minutes.

Do you have any questions before we start?

The first few questions are around what measures you currently use and how you use them.

- Are you able to tell me what palliative care outcome measures you use in your everyday practice? (probe for use of the three core measures: palliative Phase of Illness, AKPS, and IPOS)
- When were these measures introduced? (probe for when each of the three core was introduced)
- How do you use these measures in practice? Probe for:
  - Who collects these measures?
  - How often they are collected/timings?

Version 1 (15/12/2020)

  - Which version is used (largely version of IPOS, but also version of Barthel if mentioned)?

- Has your use of outcome measures changed over the last 1-2 years?
  - Collecting?
  - Scoring?
  - Reporting?
  - General understanding of each measure (i.e., what they are, how to use them, their value)?
- Do you think that the way in which you currently use outcome measures is better or worse than previously?
  - What has changed or remained the same?
  - Why?

The next few questions focus on the ways in which you may have used different components of the RESOLVE implementation strategy and how, if at all, you feel as though it may have impacted how you use outcome measures.

- Have you, or your team/service, used any of the RESOLVE implementation strategies
  - Educational resources
  - Workshop and conference events
  - Determining organisational and team needs
  - Formal and informal training
  - IT support
  - Reporting and feedback
  - Quality Improvement Facilitator, or other RESOLVE team members

Version 1 (15/12/2020)

- Can you comment on the extent to which you feel the RESOLVE implementation has been put into practice within your service?
    - Which elements?
    - How much?

Coherence

- What do outcome measures mean to you in relation to your role?
    - Have these views changed over time?
    - Why?
- Do you feel as though the RESOLVE implementation strategy has changed your own (and other colleagues') understandings of what outcome measures are and how to use them?
    - On scoring consistently and confidently?
    - On what they mean (especially Phase of Illness)?
    - Frequency of collection (and selecting the relevant versions)?
- How and why do you think these things?

Cognitive Participation

- Are you able to reflect on the ways in which the RESOLVE implementation strategy may have contributed to building clinical/team practice around outcome measures?
    - Has it influenced communicating with outcome measures in relation to individual patients (i.e., ward rounds, MDT's, handovers etc.)?
    - How?
    - Has it shaped the services that your team or organisation delivers?
    - How?

Version 1 (15/12/2020)

Collective Action

- Thinking back over the last year, has the way in which you use outcome measures (both individually and as part of your team) changed as a result of the RESOLVE implementation strategy?
    - How?
    - What is different now?
    - What parts of the RESOLVE implementation strategy do you feel are responsible for these changes?
- Has the implementation strategy helped to develop your own, and your teams, skills, and confidence in using outcome measure to inform care at a patient/team/service level?
    - What has helped with this?
- Were there any organisational barriers to using outcome measures (individually and as part of your team) that the RESOLVE implementation strategy has helped to address?
    - What were these?
    - How/why did the implementation strategy help?
- Has your confidence to confidently/efficiently input, share, and extract outcomes data within your electronic systems changed?
    - If any, what part of the RESOLVE implementation strategy has been key to this?

Reflexive Monitoring

- Are you able to reflect on the ways in which the RESOLVE implementation strategy has changed your views on how you value outcome measures in everyday practice?

  - How?
  - Why?
- How do you share feedback on outcome measures?
  - Do you feel as though the RESOLVE implementation strategy has had an impact on for you/your team/the organisation to do this?
  - How/Why?
- Are there any aspects of the RESOVLE implementation strategy that you think have contributed/will contribute to sustained use of outcome measures?
  - What are these?
  - Why?
  - How will use be sustained once the RESOLVE project is over?

Thank you for taking part in this interview. Before we finish, do you have any other questions?
