# Supplementary material for: Implementing person-centred outcome measures (PCOMs) into routine palliative care: A protocol for a mixed-methods process evaluation of The RESOLVE PCOM Implementation Strategy
Source: BMJ Open. 2021 Sep 3;11(9):e051904. doi: 10.1136/bmjopen-2021-051904 (PMC8420722; doi:10.1136/bmjopen-2021-051904)
Supplement: Author's manuscript [file bmjopen-2021-051904.draft_revisions.pdf]

BMJ Open

BMJ Open is committed to open peer review. As part of this commitment we make the peer review history of every article we publish publicly available.

When an article is published we post the peer reviewers' comments and the authors' responses online. We also post the versions of the paper that were used during peer review. These are the versions that the peer review comments apply to.

The versions of the paper that follow are the versions that were submitted during the peer review process. They are not the versions of record or the final published versions. They should not be cited or distributed as the published version of this manuscript.

BMJ Open is an open access journal and the full, final, typeset and author-corrected version of record of the manuscript is available on our site with no access controls, subscription charges or pay-per-view fees (http://bmjopen.bmj.com).

If you have any questions on BMJ Open's open peer review process please email info.bmjopen@bmj.com

BMJ Open

Implementing Person-Centred Outcome Measures (PCOMs) into routine palliative care: Protocol for a mixed-methods process evaluation of the RESOLVE PCOM Implementation Strategy

Journal:	*BMJ Open*
Manuscript ID	bmjopen-2021-051904
Article Type:	Protocol
Date Submitted by the Author:	01-Apr-2021

Complete List of Authors:	Bradshaw, Andy; University of Hull<br>Santarelli, Martina; University of Hull<br>Khamis, Assem M; Hull York Medical School<br>Sartain, Kathryn; University of Hull; York and Scarborough Teaching Hospitals NHS Foundation Trust<br>Johnson, Miriam; The University of Hull, Hull York Medical School<br>Boland, Jason; Hull York Medical School<br>Pearson, Mark; Hull York Medical School, Wolfson Palliative Care Research Centre<br>Murtagh, Fliss; Wolfson Palliative Care Research Centre, Hull York Medical School, University of Hull, UK

Keywords:	PALLIATIVE CARE, Protocols & guidelines < HEALTH SERVICES ADMINISTRATION & MANAGEMENT, Adult palliative care < PALLIATIVE CARE

SCHOLARONE™
Manuscripts

I, the Submitting Author has the right to grant and does grant on behalf of all authors of the Work (as defined in the below author licence), an exclusive licence and/or a non-exclusive licence for contributions from authors who are: i) UK Crown employees; ii) where BMJ has agreed a CC-BY licence shall apply, and/or iii) in accordance with the terms applicable for US Federal Government officers or employees acting as part of their official duties; on a worldwide, perpetual, irrevocable, royalty-free basis to BMJ Publishing Group Ltd ("BMJ") its licensees and where the relevant Journal is co-owned by BMJ to the co-owners of the Journal, to publish the Work in this journal and any other BMJ products and to exploit all rights, as set out in our licence.

The Submitting Author accepts and understands that any supply made under these terms is made by BMJ to the Submitting Author unless you are acting as an employee on behalf of your employer or a postgraduate student of an affiliated institution which is paying any applicable article publishing charge ("APC") for Open Access articles. Where the Submitting Author wishes to make the Work available on an Open Access basis (and intends to pay the relevant APC), the terms of reuse of such Open Access shall be governed by a Creative Commons licence – details of these licences and which Creative Commons licence will apply to this Work are set out in our licence referred to above.

Other than as permitted in any relevant BMJ Author's Self Archiving Policies, I confirm this Work has not been accepted for publication elsewhere, is not being considered for publication elsewhere and does not duplicate material already published. I confirm all authors consent to publication of this Work and authorise the granting of this licence.

For peer review only

Title: Implementing Person-Centred Outcome Measures (PCOMs) into routine palliative care: Protocol for a mixed-methods process evaluation of the RESOLVE PCOM Implementation Strategy

Bradshaw, A [1]., Santarelli, M [1]., Khamis, A [1]., Sartain, K [1,2]., Johnson, M. J [1]., Boland, J. W [1]., Pearson, M [1]., Murtagh, F.E.M [1].*

[1] *Wolfson Palliative Care Research Centre, Hull York Medical School, University of Hull, Hull, UK*

[2] *York and Scarborough Teaching Hospitals NHS Foundation Trust, York, UK*

*Corresponding author: Andy Bradshaw, Allam Medical Building, University of Hull, Hull, HU6 7RX. Email: andrew.bradshaw@hyms.ac.uk

Word count (excluding title page, abstract, references, figures and tables): 3,977

For peer review only

For peer review only - http://bmjopen.bmj.com/site/about/guidelines.xhtml

Abstract

Introduction: Person-centred outcome measures improve quality of care and patient outcomes but are used inconsistently in palliative care practice. To address this implementation gap, we developed the 'RESOLVE Implementation Strategy'. This protocol outlines a process evaluation to explore mechanisms through which this strategy does, or does not, support the implementation of outcome measures in routine palliative care practice.

Methods and analysis: Multistrand, mixed methods process evaluation. Strand one will collect routine outcomes data to map the changes in use of outcome measures over time. Strand two will collect survey data to explore how professionals' understanding of, skills in using, and ability to build organisational practices around, outcome measures change over time. Strand three will collect interview data to understand the mechanisms underpinning/affecting our implementation strategy. Thematic Framework analysis and descriptive statistics will be used to analyse qualitative and quantitative data, respectively.

Ethics and dissemination: For strand one, ethical approval has been obtained (Cambridge REC, REF: 20/EE/0188). For strands two and three ethical approval has been obtained from Hull York Medical School ethics committee [21 05]. Tailored feedback of study findings will be provided to participating sites. Abstracts and papers will be submitted to national/international conferences and peer-reviewed journals. Lay and policy briefings and newsletters will be shared through PPI and project networks, plus via the project website.

Keywords: Implementation science; Outcome measures; Palliative care; Process evaluation

For peer review only - http://bmjopen.bmj.com/site/about/guidelines.xhtml

Strengths and limitations of this study

- This is the first process evaluation of an implementation strategy designed to facilitate the integration of Person-Centred Outcome Measures into routine palliative care practice.
- This protocol follows the Medical Research Council's guidance on process evaluations by adopting a systematic and theoretically informed approach to study design and conduct.
- We will adopt a multi-strand, mixed methods approach in which we will integrate routine outcomes, survey, and focused interview data to demonstrate the mechanisms through which our intervention strategy may or may not work across different settings of palliative care.
- Since we only focus on implementing a core set of outcome measures, the applicability of findings may be limited to other outcome measures, many of which have measure-specific challenges to implementation.

For peer review only - http://bmjopen.bmj.com/site/about/guidelines.xhtml

INTRODUCTION

Person-Centred Outcome Measures in Palliative Care

Palliative care aims to alleviate suffering due to progressive, life-limiting medical conditions and improve quality of life through the adoption of a holistic, person-centred, and multidisciplinary approach that is delivered across multiple settings (in-patient, outpatient, hospital, and community).[1] It is projected that by 2060, the global need for palliative care will double (from 26 million to 48 million people).[2] It is important, therefore, that we are able to clearly demonstrate the quality of palliative care services by showing if and how they are able to improve a person's symptoms, and sustain or improve wellbeing and functional ability. To do this, focusing on outcomes is essential.

In a healthcare context, an outcome refers to 'the change in a patient's current and future health status that can be attributed to preceding healthcare'.[3] Within palliative care, measuring outcomes is important because they allow us to evaluate whether the care that is given to patients and their families makes a difference to their quality of life. [4] In capturing this information, Person-Centred Outcome Measures (PCOMs) are considered the 'gold standard' for outcome measurement in palliative care. [5, 6] These are standard, validated questionnaires – usually filled in by patients themselves - that provide healthcare professionals with important information on a person's own perception of their health status and well-being. [7] Moreover, because many patients with life-limiting illnesses may have impaired cognition and/or be too unwell to fill out PCOMs on their own accord, [8] PCOMs may sometimes be completed using proxy-reported ratings (e.g., by healthcare professionals and/or a patient's family member).

Current evidence demonstrates the value of PCOMs in palliative care in helping to identify (often unrecognised), continually monitor, and take direct clinical action to address, a

For peer review only - http://bmjopen.bmj.com/site/about/guidelines.xhtml

person's needs/symptoms; facilitate communication between patients and clinicians; and improve outcomes. [4, 7, 9] This is through using PCOMs at individual/patient (e.g., for clinical assessment), team (e.g., focusing patient reviews and workload planning), service (e.g., service evaluation/development and making business cases), and population (e.g., benchmarking) levels of practice. [10] For these reasons, the European Association for Palliative Care Task Force on Outcome Measurement recommended that PCOMs should be firmly embedded into routine clinical practice across all settings in which palliative care is delivered. [6]

Despite the evidence underpinning the value of using PCOMs, they are used inconsistently in clinical practice, if at all. Reasons for this include issues such as time constraints, lack of training and knowledge, tools being perceived as burdensome, negative attitudes towards outcome measures, inefficient electronic systems, top-down approaches to implementation, and fear of added work. [5, 11-13] In addressing these challenges and facilitating the implementation of PCOMs into everyday clinical practice, various studies have provided tables of recommendations to consider before and during implementation. [5, 12, 13] These have recommended focusing on individual, management, and organisational issues during the preparation and roll-out stages of implementation. To date, however, there has been no attempt to develop these into, and subsequently evaluate, an implementation strategy that may be applied across all palliative care settings.

The RESOLVE project

The RESOLVE study is a multi-site implementation and research programme aimed at implementing PCOMs into routine clinical practice in 11 specialist palliative care services across Yorkshire, England. These services vary in size, funding source (e.g., charitable/NHS), location (e.g., urban/semi-rural/rural), and span inpatient, outpatient/day therapy, and home-based/community settings. As part of this study, we conducted an exploratory qualitative

For peer review only - http://bmjopen.bmj.com/site/about/guidelines.xhtml

study in order to understand the processes and causal mechanisms that underpin the successful implementation of PCOMs into routine practice. [13] By using these insights and collaborating closely with sites, we have developed a complex intervention – named 'The RESOLVE PCOM Implementation Strategy' – aimed at facilitating the integration of PCOMs into routine practice. It is important to evaluate these types of interventions to understand what does (and does not work) and in what contexts.

Process evaluations are useful in understanding the ways in which complex interventions work because they provide insight into the causal mechanisms and contextual factors at play during implementation. [14] Currently, however, no process evaluations have been conducted that explore the impact of implementation strategies aimed to promote the uptake and sustained use of PCOMs within palliative care contexts.

Therefore, we aim to conduct a process evaluation that explores the mechanisms through which The RESOLVE PCOM Implementation Strategy does, or does not, support the implementation of PCOMs in routine palliative care practice. We aim to achieve this by answering three research questions:

1. Does The RESOLVE PCOM implementation strategy enhance the uptake and routine use of PCOMs?
2. Does The RESOLVE PCOM implementation strategy enhance health care professionals' individual and collective understanding of, skills in using, and ability to build organisational practices around, PCOMs?
3. If The RESOLVE PCOM implementation strategy is effective, through which mechanisms are its intended effects achieved?

For peer review only - http://bmjopen.bmj.com/site/about/guidelines.xhtml

METHODS AND ANALYSIS

Study design

A multistrand, mixed methods, longitudinal process evaluation informed by The Medical Research Council guidance. [15] To do this, we will adopt a systematic approach in which we provide clear descriptions of, and collect relevant data pertaining to: (i) our complex intervention; (ii) the implementation process; (iii) mechanisms; (iv) context; and (v) outcomes/results (see Table 1). This will be achieved through three strands of work. An overview these can be seen in Table 2.

Core PCOMs set

The three outcome measures we aim to implement are a nationally-recognised and validated core set of PCOMs for specialist palliative care. [16] These measures are based on what patients and their families prioritise as important in advanced illness and have been endorsed by NHS England and Public Health England. They include the palliative Phase of Illness, [17] Integrated Palliative care Outcomes Scale (IPOS), [18, 19] and Australia-modified Karnofsky Performance Status (AKPS). [20] More information about each of these measures can be seen in Table 3.

The implementation of these PCOMs is not simply about increased use, but the wrap-around work that is needed for the effective and appropriate use of these measures to improve care. This includes: organizational and team recognition of why using measures is important, using the right measures at the right time, ensuring follow-up assessments using the measures, getting all teams/team members on board, having efficient feedback systems in place, having all members of the team understanding the purpose of PCOMs, and embedding PCOMs into the 'cultural fabric' of how teams and services operate. [13]

For peer review only - http://bmjopen.bmj.com/site/about/guidelines.xhtml

Table 1: Key components of a process evaluation and how this study has considered these. Grey shaded areas represent key components of a process evaluation according to MRC guidance.

**Key components of process evaluations**	**MRC guidance** [15]	**This study**
**Describing the complex intervention and clarifying causal assumptions**	'A clear description of the intended intervention, how it will be implemented, and how it is expected to work, will ideally have been developed before evaluation'	We clearly describe The RESOLVE PCOM Implementation strategy and have drawn on Normalisation Process Theory to explain the causal assumptions through which we expect it to work (see pages 10-12)
**Implementation: what is implemented, and how?**	• 'Implementation process (How delivery is achieved; training, resources etc.)'<br>• 'What is delivered (fidelity, dose, adaptations, reach)'	We have outlined, in detail, the features of our complex interventions using the TIDieR checklist (see supplementary file 1). This outlines what each component is, why we are doing it, who is delivering, how much they are delivering, where and when it is being delivered, how it may be tailored/modified, and how well it will be delivered (e.g., fidelity).
**Mechanisms of impacts**	• 'Participant responses to and interactions with the intervention'<br>• 'Mediators'<br>• 'Unexpected pathways and consequences'	In understanding mechanisms of impacts, we are collecting three types of data:<br>- Routinely collected PCOMs data (see pages 13-14).<br>- Survey data (see pages 14-17).<br>- Focused interview data (see page 17-19).
**Context**	• 'Contextual factors that shape theories of how the intervention works'<br>• 'Contextual factors that affect (and may be affected by) implementation, intervention mechanisms and outcomes'<br>• 'Causal mechanisms present within the context which act to sustain the status quo, or potentiate effects'	Analysis of focused interview data which provide insight into the contextual factors and mechanisms (e.g., settings of care or organisational characteristics) that affect (and are affected by) the implementation process. (see page 19).
**Outcomes**	Analysis of process data, and integration of process and outcome data	We will combine results/analysis from quantitative and qualtiative analyses to produce numerous outputs that answer the research questions of this study. (see page 19-20).

For peer review only - http://bmjopen.bmj.com/site/about/guidelines.xhtml

Table 2: An overview of the research design for this study

Strand	Data collection method	Data collection timepoints	Reason/output
**1**	Routine outcomes	Baseline and follow-up	To map changes in use (i.e., implementation status) of PCOMs over time **(RQ 1)**
**2**	Survey	Initial piloting phase and then at baseline and follow-up	To determine how changes in PCOM use relates to changes in participants' coherence, engagement, action, and reflexive monitoring of measures **(RQ 2)**
**3**	Focused interviews	Towards end of project	To determine why? how? context? Of any changes (i.e., mechanisms) **(RQ 2&3)**

Table 3: Description of the core set of PCOMs that we aim to implement into routine palliative care practice as part of the RESOLVE project.

Measure	Description
palliative Phase of Illness [17]	Palliative Phase of Illness is a measure which describes the urgency of care needs for a person receiving palliative care. It does so by describing four distinct phases of a patient's illness, including: stable, unstable, deteriorating, dying, and deceased. These Phases are measured through determining the care needs of a patient and/or their family and provide a clinical indication of a patient's Phase of Illness which can be used to inform care planning.
Integrated Palliative care Outcomes Scale (IPOS) [18, 19]	IPOS is a holistic, well-validated, and global measure of symptom burden that uses 10 questions (scored on a 0-4 Likert type scale) to assess the most important symptoms and concerns of patients affected by life-limiting illnesses across physical, psychological, social, and existential domains of well-being. There are two forms of IPOS; patient-IPOS (where patients complete the questionnaire as a self-report) and staff-IPOS (a proxy version which is completed by staff).
Australia-modified Karnofsky Performance Status (AKPS) [20]	The AKPS assesses a patient' overall performance/functional status across 3 dimensions: activity, work, and self-care. Healthcare professionals use their observations of patients' ability to perform everyday tasks and scores them at 10% increments between 0% (i.e., the patient is dead) and 100% (i.e., no complaints or evidence of disease).

For peer review only

For peer review only - http://bmjopen.bmj.com/site/about/guidelines.xhtml

Description and causal assumptions of the RESOLVE PCOM implementation strategy

Description of complex intervention

In facilitating the implementation of PCOMs into routine practice we have worked collaboratively with specialist palliative care services and health professionals to develop 'The RESOLVE PCOM Implementation Strategy'. This represents a complex intervention in that it 'comprise[s] [of] multiple interacting components' (see Table 4 for a detailed breakdown of each component). [15] The first two of these are general elements that are mostly pre-specified and provided to sites in a standardised manner. The next four are tailored closely to the needs of each site. The final component of this implementation strategy is the role of the quality improvement facilitator which permeates both general and site-specific implementation strategies.

All elements of this implementation strategy are rooted in an assessment of learning needs at each site, the development of rapport with staff, and a visible response to ongoing feedback. They are intended to empower clinical staff to appropriately use PCOMs. This may take the form of sharing formal knowledge about PCOMs and how to use them, practical and technical knowledge about IT systems, and/or experiential knowledge about the use of PCOMs in practice. Where PCOM use is affected by team or organisational issues, quality improvement facilitators can empower clinical staff to address these issues and may also play an advocacy role through liaison with senior clinical staff or management. A more in-depth version of the RESOLVE PCOM implementation strategy (mapped against the different components included in the TIDIER checklist [21]) can be found in supplementary file 1.

For peer review only - http://bmjopen.bmj.com/site/about/guidelines.xhtml

Table 4: An outline of the main components of the RESOLVE PCOM implementation strategy

Implementation component	Characteristics
**General implementation strategies**	
**1. Development of educational resources**	Providing healthcare professionals with resources (e.g., brief instructional videos and pocket guides/outcomes manual) aiming to enhance their knowledge and skills of what outcome measures are, and how to use the right measures at the right time in clinical practice
**2. Workshop and conference events**	Developing and running national and regional workshop events that aim to address common challenges to implementation and facilitate collaborative learning networks between palliative care services
**Site-specific implementation strategies**	
**3. Determining organisational needs**	A baseline assessment - developed through initial interview data and additional liaison with service leads at each site – aimed at understanding the site-specific challenges to implementing PCOMs so that the intervention can be tailored to each site's needs
**4. Formal training**	Face-to-face and online educational sessions/presentations with healthcare professionals at sites in order to address local implementation issues/challenges
**5. I.T. support**	Providing informational, practical, and technical support to ensure that each site has the analytic capacity to input outcomes data into, and extract it back out of, electronic systems
**6. Reporting and feedback**	Providing sites with tailored, service-level reports and feedback of their outcomes data to motivate continued use
**Running through general and site-specific implementation strategy**	
**7. Quality improvement facilitator**	Working directly with sites by local site liaison, championing the implementation of PCOMs into practice, identifying and responding to local challenges/needs, keeping PCOMs on the agenda, and acting as a direct link between the research team and sites. An important aspect of this role is to make judicious use of the implementation strategy elements, rather than necessarily deliver the strategy as a whole

Theoretical approach and causal assumptions

As part of their guidance on process evaluations, Moore et al. [15] recommend drawing on theory to propose causal assumptions underpinning how and why The RESOLVE PCOM Implementation Strategy is expected to work. To this end, the components of our implementation strategy are theoretically informed by Normalisation Process Theory (NPT). [22, 23] The reason behind selecting NPT is because there is evidence of its effectiveness and value in implementing complex interventions within healthcare settings. [23]

For peer review only - http://bmjopen.bmj.com/site/about/guidelines.xhtml

NPT is a type of implementation theory that describes the different types of 'work' that individuals and organisations do in order to 'normalise' complex interventions into clinical practice (i.e., such deep embedment that they become 'invisible'). It does so through proposing four inter-related constructs:

Coherence: The 'sense-making work' that individuals and teams do in order to understand PCOMs.

Cognitive participation: The 'relational work' that occurs in legitimising PCOMs and building everyday clinical practice around using them.

Collective action: The 'operational work' that is performed in order to use PCOMs, including the skills-sets of a workforce and the organisational resources in place that enable the use of PCOMs.

Reflexive monitoring: The 'appraisal work' through which people assess and evaluate the relative value of PCOMs. [22, 24]

We therefore propose that the RESOLVE PCOM implementation strategy will facilitate the implementation of PCOMs into routine practice in four ways: (1) by enhancing professionals' understanding and awareness of how and why to use PCOMs (i.e., improving coherence); (2) by creating an organisational culture where PCOMs are valued and seen as a legitimate way to improve patient care (i.e., enhancing cognitive participation); (3) by ensuring that systems and resources are in place that allows healthcare professionals to easily and meaningfully use PCOMs in everyday practice (i.e., facilitating collective action); and (4) by providing meaningful feedback on PCOMs data as to motivate people to continue using them (i.e., enhancing reflexive monitoring).

For peer review only - http://bmjopen.bmj.com/site/about/guidelines.xhtml

Patient and public involvement

We support the UK Standards for Public Involvement in research as outlined by the National Institute of Health Research. [25] We have already conducted patient public involvement for strand one of this project (the collection and use of routine outcomes data) in which we consulted with a diverse group of patients about implementation. Furthermore, the RESOLVE study has recruited a lay representative who attends regular project meetings and will be involved in the discussions regarding the interpretation and dissemination of study findings throughout the duration of the project.

Strand one: Mapping change through routinely collected outcomes data

Sampling and recruitment

In recruiting to this study, we will send an invitation letter and participant information sheet to local collaborators at each site. Should sites wish to participate, they will be required to sign and return the study invitation letter. We expect all 11 RESOLVE-affiliated sites to participate in this project.

Data collection

The RESOLVE team have already been working with sites in implementing the palliative Phase of Illness, Integrated Palliative care Outcomes Scales (IPOS), and Australia-modified Karnofsky Performance Status (AKPS) into routine clinical practice. Sites will be required to submit pseudonymised data related to these PCOMs to the RESOLVE team at baseline and follow-up (between 10-12 months later). A more detailed description of the data specification can be found in supplementary file 2.

For peer review only - http://bmjopen.bmj.com/site/about/guidelines.xhtml

Data Analysis

In order to map whether or not the uptake and routine use of PCOMs change over time, we will use descriptive and analytical statistics to report the levels of missing data at baseline and follow-up (missing complete measures, and missing items).

For each site at baseline, we will assess the missing outcome data at three timepoints: a) start of episode of care, b) first change of Phase of Illness, and c) end of episode of care. To highlight the change in routine use of PCOMs, we will compare the missing outcome data between baseline and follow-up at these 3 timepoints. We will assess missing data on the level of these subscales: physical symptoms (10 items), psychological/emotional symptoms (4 items), and communication/practical issues (3 items). We defined missing outcome data in physical symptoms as 4 or more items missing data out of the 10 items at one point of time. For emotional and communication subscales, missing outcome data was defined as missing at least one item in any of them. We will also compare between the population samples at baseline and follow-up to investigate the comparability of both groups. To do this we will use a t-test to compare continuous data (e.g., age) with mean and SD and chi-square to compare categorical data (e.g., gender).

Strand two: Online survey data

Conducting survey research will help us to understand whether The RESOLVE PCOM Implementation Strategy has enhanced health care professionals' understandings of, skills in using, and ability to build organisational practices around, PCOMs. The design of the survey strand of this project is informed by Kelley et al.'s [26] guidance on good practice on the conduct and reporting of survey research.

Sampling and recruitment

For peer review only - http://bmjopen.bmj.com/site/about/guidelines.xhtml

We estimate that, when combined, the number of staff across all of the participating services equates to approximately 550 participants. Of this number, due to the positive relationships that we have fostered with sites throughout this project, we expect between a 50%-60% response rate. Thus, for the survey part of this research, we estimate between 275-330 participants.

We will use a purposive maximum variation sampling technique [27] as a guide for recruitment, and sample across the following purposive criteria: role, seniority, experience, and settings of care (inpatient, outpatient/day therapy, home-based/community). Through recruiting in this way, we aim to sample an accurate cross-section of the different settings in which PCOMs are used, and the different types of staff members who use them within these settings. This will translate into recruiting staff at different levels/seniority (e.g., those at a medical, senior staff, and junior level) and of differing roles (e.g., doctors, nurses, healthcare assistants, educators, allied healthcare professionals).

To recruit participants, local collaborators at each site will be asked to disseminate an online survey link to healthcare professionals within their organisations. The introductory part of the survey will include the relevant participant information that explains what the purpose of the survey is, why they have been selected, and how data will be used. It will also state that by completing and submitting the survey that they are providing consent to participate in this study. To optimise recruitment and survey completion, we will offer participating sites the incentive of a textbook and/or journal subscription for the organisation for one year.

Data collection

In keeping with the theoretical framework of this project, we will collect survey data using an adapted version of the NPT survey (The Normalisation MeAsure Development questionnaire

For peer review only - http://bmjopen.bmj.com/site/about/guidelines.xhtml

(NoMAD). This is a 23-item structured questionnaire that has been developed by the authors of NPT and measures the dynamic processes that underpin the implementation of PCOMs into clinical practice from the perspectives of healthcare professionals directly involved in their use. The NoMAD measure has been found to be an acceptable and psychometrically strong measure which reports good face validity, construct validity, and internal consistency.[28]

Our version of the NoMAD survey consists of three parts which comprise of a combination of closed- and open-ended questions. Part A asks participants to provide demographic information about themselves. Part B asks short questions on what PCOMs participants use and how they use them in everyday practice. Part C is split into four sections, each of which corresponds and ask questions in relation to the different constructs of NPT (coherence, cognitive participation, collective action, reflexive monitoring). This survey can be seen in more detail in supplementary file 3.

We will host our survey online using RedCap software in which participants will be sent a link through which they are able to access and complete the survey. The acceptability of this method, alongside the intelligibility of survey questions, will be piloted on a select number of participants to ensure that they understand the questions being asked and are easily able to complete the online form. Following this, we will make any necessary minor amendments to the NoMAD survey and then administer this to participants at baseline and then at follow-up (10-12 months after baseline collection).

Data analysis

We will conduct four analyses of survey data:

i. Response rate by capturing the number of participants who completed the NoMAD survey compared to the proportion of those who were approached.

For peer review only - http://bmjopen.bmj.com/site/about/guidelines.xhtml

ii. Descriptive statistics to describe the general characteristics of healthcare staff who participated in the survey.

iii. Comparison between respondent answers at baseline and follow-up using statistics tests such as paired t-test and McNemar test (when needed) to indicate any changes in PCOMs usage and understanding. We recognise that there are numerous potential limitations/challenges of conducting follow-up surveys 12 months after baseline. These include losing participants to follow-up, withdrawal, staff turnover, changed positions, each of which may lead to decrease the sample size at follow-up time. Thus, we decided if any group lost more than 70% of its participants, we will recruit other health staff from the site (who did not participate at baseline) to complete our follow-up sample. Therefore, we will change the analysis plan and treat both groups two independent samples.

iv. Free-text survey responses will be deductively thematically analysed using NPT constructs (coherence, cognitive participation, collective action, reflexive monitoring) as a framework, whilst also allowing for inductive thematic analysis if responses do not fit within the framework.

Strand three: Focused interview data

For strand three, we will collect focused interview data. The collection of qualitative interview data will be able to provide further insight into research questions two and three by providing rich and insightful data on the processes, mechanisms, and contextual factors that underpin the ways in which The RESOLVE PCOM Implementation Strategy does, or does not, work to implement PCOMs into routine practice. [29]

Sampling and recruitment

For peer review only - http://bmjopen.bmj.com/site/about/guidelines.xhtml

For each site, depending on organisational size, we will recruit between two-five participants. To do this, we will use purposive sampling [27] (based on roles, disciplines, and experience) as a guide to recruit staff who are directly involved in the implementation of PCOMs across different settings, and able to offer a distinct insight/experience into the issues identified from the survey about each of the NPT constructs. Recruitment will be achieved through working with service leads at each site to approach the relevant participants for interview in this study. Participants will then be approached by their service leads with a participant information sheet. Willing participants will then be required to provide informed consent prior to participation.

Data collection

We will conduct a single focused interview with each participant towards the end of the project (i.e., when we finish our implementation work). These interviews will be used as an opportunity to generate rich insight and details of the processes, mechanisms, and contextual factors that underpin how The RESOLVE PCOM Implementation Strategy does, or does not, work to facilitate the implementation of PCOMs into routine palliative care practice. The interview guide (see supplementary file 4) will consist of a series of questions that relate to the different components of NPT (coherence, cognitive participation, collective action, reflexive monitoring) and will present participants with an opportunity to reflect on how their use of PCOMs has changed over time, and how The RESOLVE PCOM Implementation Strategy has impacted this. In mitigating for the COVID-19 pandemic, these interviews will take place online (on whichever platform is most convenient for services) at a time that works for them.

Data analysis

For peer review only - http://bmjopen.bmj.com/site/about/guidelines.xhtml

Data will be analysed in NVivo using a thematic framework approach. [30] NPT constructs (coherence, cognitive participation, collective action, and reflexive monitoring) will provide a theoretical framework that will be applied deductively to interview transcripts, whilst also allowing for inductive thematic analysis of responses and outliers that do not fit within the framework. This will be through conducting the following interconnected steps: (1) transcription; (2) familiarisation; (3) coding; (4) developing an analytic framework based on NPT; (5) indexing this framework to transcripts; (6) charting data into a framework matrix (and then performing between- and within-case analyses for each theme); and (7) interpreting the data by using theory.

Outputs: Integration of datasets

Each strand of this project will produce different outputs. In strand one, we will use routine outcomes data to produce a traffic light system in which green represents that PCOMs are used appropriately, amber that PCOMs are used but inconsistently, and red that PCOMs are not being used at all. At a glance, this will provide an objective indicator of whether there has been a change (from baseline to follow-up) in the uptake and use of each PCOM across settings, thus enabling us to determine whether the RESOLVE implementation strategy has been successful in implementing PCOMs into routine palliative care practice.

For strand two, we will produce outputs for NoMAD survey data in the form of spider plot graphs. By calculating how mean changes in each NPT component changes from baseline to follow-up, this data will be able to complement our analyses of routine outcomes data by seeing if changes in use of PCOMs status coincides with changes in components of NPT.

For strand three, data from interviews will complement routine outcomes and NoMAD survey data by illuminating the mechanisms as to how and why the use of PCOMs

For peer review only

For peer review only - http://bmjopen.bmj.com/site/about/guidelines.xhtml

have (or have not) changed over time. It will also be able to provide important information about the contextual/organisational factors that impacted this process.

Figure 1 demonstrates how the integration of outputs produced from the analysis of quantitative and qualitative data may look. It will provide a snapshot that will be able to demonstrate the effectiveness The RESOLVE Implementation Strategy at facilitating the implementation of PCOMs into routine palliative care practice. It will also provide us with explanations as to how, why, and in what contexts our intervention works/needs refining so that it can be tailored for particular organisational contexts and/or settings of care.

[INSERT FIGURE 1 HERE]

For peer review only

For peer review only - http://bmjopen.bmj.com/site/about/guidelines.xhtml

ETHICAL CONSIDERATIONS

Ethical approval for this project was obtained on 16th September 2020 from the East of England - Cambridge Central NHS Research Ethics Committee [ref: 20/EE/0188]. Ethical approval for strands 2 and 3 of this study was obtained on 18th January 2021 from Hull York Medical School ethics committee [21 05].

DISSEMINATION

We will publish the findings of this study in peer reviewed journals and abstracts will be submitted to national and international conferences. We will also disseminate this work to patients, healthcare professionals, academics, and anybody else who is interested in this work through various non-traditional routes. These will include:

- Producing lay briefs of research findings that we will disseminate to patients and service users through our Palliative Daycare Patient Group and the PPIE network at the University of Hull.
- Producing policy briefs of research findings for policymakers and commissioners
- Sharing the results of research and study newsletters on the RESOLVE and Wolfson Palliative Care Research Centre websites and sharing them on social media.
- Delivering educational workshops to clinicians in order to feedback site-specific findings on what does and does not work with regards to implementing PCOMs within their service
- Utilise the Hospice UK's ECHO Network for Outcomes - monthly national interactive webinars to disseminate the results to clinicians, allied health-care professionals, and others who have an interest in palliative care.

For peer review only

For peer review only - http://bmjopen.bmj.com/site/about/guidelines.xhtml

Author contributions: Conceptualisation and design – AB, AK, MP, and FEM. Development of analysis plan – AB, AK, and FEM. Leading the writing process and drafting the original article – AB. Critically reviewed article for important content – AB, MS, AK, KS, JB, MJ and FEM. All authors take responsibility for the protocol and approved the final version of this paper.

Funding statement: This work was supported by Yorkshire Cancer Research, grant number [L412]. Professor Fliss Murtagh is a National Institute for Health Research (NIHR) Senior Investigator. The views expressed in this article are those of the author(s) and not necessarily those of the NIHR, or the UK Department of Health and Social Care.

Competing interest's statement: The author(s) declared no potential conflicts of interest with respect to the research, authorship, and/or publication of this article.

For peer review only

For peer review only - http://bmjopen.bmj.com/site/about/guidelines.xhtml

References

1. Twycross RG. *Introducing palliative care*. Radcliffe Publishing, 2003.
2. Sleeman KE, de Brito M, Etkind S, et al. The escalating global burden of serious health-related suffering: projections to 2060 by world regions, age groups, and health conditions. *The Lancet Global Health* 2019; 7: e883-e892. DOI: 10.1016/S2214-109X(19)30172-X.
3. Donabedian A. *Explorations in quality assessment and monitoring*. Ann Arbor, MI: Health Administration Press, 1980.
4. Dudgeon D. The impact of measuring patient-reported outcome measures on quality of and access to palliative care. *Journal of palliative medicine* 2018; 21: S-76-S-80.
5. Antunes B, Harding R, Higginson IJ, et al. Implementing patient-reported outcome measures in palliative care clinical practice: a systematic review of facilitators and barriers. *Palliative medicine* 2014; 28: 158-175.
6. Bausewein C, Daveson BA, Currow DC, et al. EAPC White Paper on outcome measurement in palliative care: Improving practice, attaining outcomes and delivering quality services–Recommendations from the European Association for Palliative Care (EAPC) Task Force on Outcome Measurement. *Palliative medicine* 2016; 30: 6-22.
7. Etkind SN, Daveson BA, Kwok W, et al. Capture, transfer, and feedback of patient-centered outcomes data in palliative care populations: does it make a difference? A systematic review. *Journal of pain and symptom management* 2015; 49: 611-624.
8. Hill N. Use of quality-of-life scores in care planning in a hospice setting: a comparative study. *International journal of palliative nursing* 2002; 8: 540-547.
9. Currow DC, Allingham S, Yates P, et al. Improving national hospice/palliative care service symptom outcomes systematically through point-of-care data collection, structured feedback and benchmarking. *Supportive Care in Cancer* 2015; 23: 307-315.
10. Greenhalgh J. The applications of PROs in clinical practice: what are they, do they work, and why? *Qual Life Res* 2009; 18: 115-123. 2008/12/24. DOI: 10.1007/s11136-008-9430-6.
11. Dunckley M, Aspinal F, Addington-Hall JM, et al. A research study to identify facilitators and barriers to outcome measure implementation. *International journal of palliative nursing* 2005; 11: 218-225.
12. Pinto C, Bristowe K, Witt J, et al. Perspectives of patients, family caregivers and health professionals on the use of outcome measures in palliative care and lessons for implementation: a multi-method qualitative study. *Annals of palliative medicine* 2018; 7: S137.
13. Bradshaw A, Santarelli M, Mulderrig M, et al. Implementing person-centred outcome measures in palliative care: An exploratory qualitative study using Normalisation Process Theory to understand processes and context. *Palliative Medicine*; 0: 0269216320972049. DOI: 10.1177/0269216320972049.
14. Craig P, Dieppe P, Macintyre S, et al. Developing and evaluating complex interventions: the new Medical Research Council guidance. *BMJ* 2008; 337: a1655. DOI: 10.1136/bmj.a1655.
15. Moore GF, Audrey S, Barker M, et al. Process evaluation of complex interventions: Medical Research Council guidance. *BMJ : British Medical Journal* 2015; 350: h1258. DOI: 10.1136/bmj.h1258.
16. Witt J, Murtagh F, de Wolf-Linder S, et al. Introducing the Outcome Assessment and Complexity Collaborative (OACC) Suite of Measures-A Brief Introduction. *Kings College London* 2014.
17. Masso M, Allingham SF, Banfield M, et al. Palliative care phase: inter-rater reliability and acceptability in a national study. *Palliative medicine* 2015; 29: 22-30.
18. Hearn J and Higginson I. Development and validation of a core outcome measure for palliative care: the palliative care outcome scale. Palliative Care Core Audit Project Advisory Group. *BMJ Quality & Safety* 1999; 8: 219-227.
19. Murtagh FE, Ramsenthaler C, Firth A, et al. A brief, patient- and proxy-reported outcome measure in advanced illness: Validity, reliability and responsiveness of the Integrated Palliative care

For peer review only - http://bmjopen.bmj.com/site/about/guidelines.xhtml

Outcome Scale (IPOS). *Palliative medicine* 2019; 33: 1045-1057. 2019/06/12. DOI: 10.1177/0269216319854264.
20. Abernethy AP, Shelby-James T, Fazekas BS, et al. The Australia-modified Karnofsky Performance Status (AKPS) scale: a revised scale for contemporary palliative care clinical practice [ISRCTN81117481]. *BMC Palliative Care* 2005; 4: 7. DOI: 10.1186/1472-684X-4-7.
21. Hoffmann TC, Glasziou PP, Boutron I, et al. Better reporting of interventions: template for intervention description and replication (TIDieR) checklist and guide. *BMJ : British Medical Journal* 2014; 348: g1687. DOI: 10.1136/bmj.g1687.
22. May CR, Mair F, Finch T, et al. Development of a theory of implementation and integration: Normalization Process Theory. *Implementation Science* 2009; 4: 29.
23. May CR, Cummings A, Girling M, et al. Using Normalization Process Theory in feasibility studies and process evaluations of complex healthcare interventions: a systematic review. *Implementation Science* 2018; 13: 80.
24. Murray E, Treweek S, Pope C, et al. Normalisation process theory: a framework for developing, evaluating and implementing complex interventions. *BMC medicine* 2010; 8: 63.
25. Research NIoH. UK standards for public involvement: Better public involvement for better health and social care research, https://www.invo.org.uk/current-work/standards/ (2021).
26. Kelley K, Clark B, Brown V, et al. Good practice in the conduct and reporting of survey research. *International Journal for Quality in Health Care* 2003; 15: 261-266. DOI: 10.1093/intqhc/mzg031.
27. Etikan I, Musa SA and Alkassim RS. Comparison of convenience sampling and purposive sampling. *American journal of theoretical and applied statistics* 2016; 5: 1-4.
28. Finch TL, Girling M, May CR, et al. Improving the normalization of complex interventions: part 2 - validation of the NoMAD instrument for assessing implementation work based on normalization process theory (NPT). *BMC Medical Research Methodology* 2018; 18: 135. DOI: 10.1186/s12874-018-0591-x.
29. Sparkes AC and Smith B. *Qualitative research methods in sport, exercise & health. From process to product*. London: Routledge, 2014.
30. Ritchie L, Lewis J, Nicholls CM, et al. *Qualitative research practice: A guide for social science students and researchers* 2ed. Los Angeles, CA: Sage, 2013.

For peer review only

For peer review only - http://bmjopen.bmj.com/site/about/guidelines.xhtml

Figure 1: Example of how outputs will be integrated after analysis.

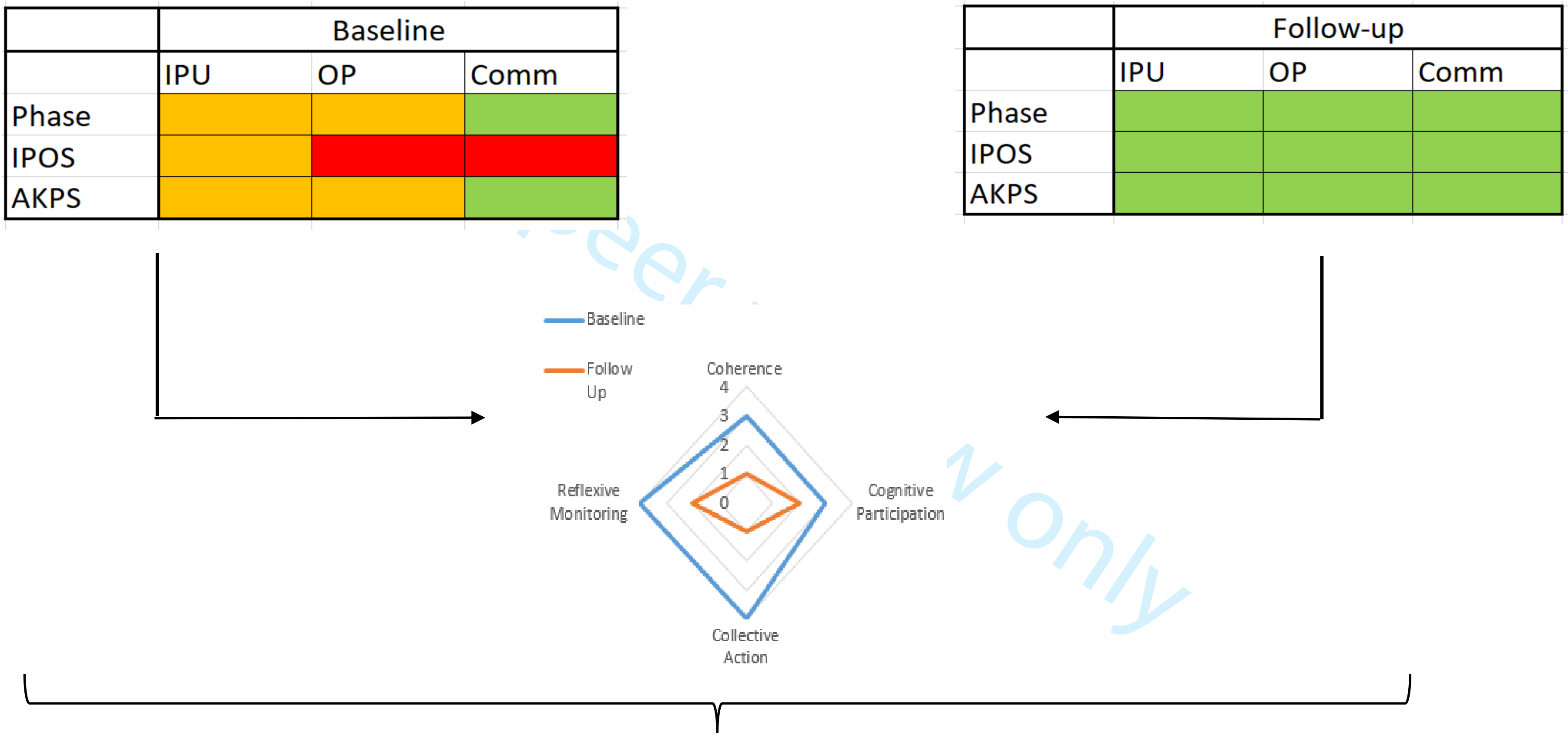

Qualitative data explaining processes, mechanisms, and contextual factors of how our complex intervention led to change.

For peer review only - http://bmjopen.bmj.com/site/about/guidelines.xhtml

Aspects of implementation									
TIDIER Checklist Components									
**Component Name**	**Why**	**What**	**Who**	**How**	**Where**	**When/How much**	**Tailoring**	**Modifications**	**How well**
**General implementation strategies**									
**Development of educational resources**	To enhance healthcare professionals' knowledge of what outcome measures are, their skills in using them, and how they may build practice around their use.	***Materials and procedures:*** Providing healthcare professionals with informational support to facilitate their understanding of what outcome measures are and how/why to use them. This is through the development of teaching and learning materials, including:<br><br>Pocket-guides, outcomes manual, instructional videos, online quizzes	Quality improvement facilitator (Martina), research nurse (Kath), PI (Fliss)	Print-based resources that can be used as an aide to memoir<br><br>Online resources (e.g., quizzes and instructional videos for each measure) available online through the RESOLVE website	Materials are for use during clinical practice and for reflection	Provided to all RESOLVE sites, with additional material being sent upon request	n/a. All sites have access to the same genenral educational resources	To complete retrospectively	To complete retrospectively
**Workshops and conference events**	Workshop and conference events aimed at responding to and discussing the common challenges that participating sites experience with regards to collecting, storing, and using outcome measures	***Materials and procedures:*** Providing informational support through teaching and guidance materials on outcome measures. This is through:<br><br>- Collective workshops and conference training events including representatives from all sites across the project	Entire RESOLVE team	Powerpoint presentations, collaborative group-based activities, question and answer sessions, and expert panel discussions.<br><br>Site representatives present at these events are encouraged to disseminate the knowledge and resources gained from attendance with their colleagues at site.	Conference/workshop events	Twice a year.	The content and agendas of workshop and conference events are derived from the common needs shared across sites participating in RESOLVE	To complete retrospectively	To complete retrospectively
**Site-specific implementation strategies**									
**Determining organisational needs**	Aimed at understanding the site-specific challenges to implementing PCOMs so that we can tailor our intervention according to each site's needs	A baseline assessment - developed through initial interview data and additional liaison with service leads at each site	Andy (research fellow) Martina (QIF)	Interviews, email, telephone communications, site visits	All sites	At the start of tailoring our intervention for each site and revisited regularly throughout implementation	Tailoring intervention components to site-specific needs	To complete retrospectively	To complete retrospectively
**Formal training**	Enhance health care professionals' individual and collective understanding of, skills in using, and ability to build organisational practices around, PCOMs	***Materials and procedures:*** Providing participating sites with informational and organisational support through teaching and guidance on outcome measures. This is through:<br><br>- Site-based training sessions	Quality improvement facilitator (Martina), research nurse (Kath), PI (Fliss)	Working with sites to understand the challenges that they face to using outcome measures in practice. This information is then used to develop formal training sessions (e.g., presentations, case studies etc.) that help in addressing these issues. This is so that we can ensure that participating sites have the knowledge, understanding, and appropriate organisational resources in place to facilitate the consistent collection and use of outcome measures	At sites	Upon request from site	The nature and content of training sessions are tailored to each site's needs and priorities	To complete retrospectively	To complete retrospectively
**I.T. support**	To ensure local I.T. system readiness. That is, making sure that participating services have the analytic capacity and infrastructure in place so that they are able to input the appropriate data items into an electronic system and extract/ submit these to the RESOLVE team	***Materials and procedures:*** Informational, practical and technical support provided to data teams/analysts to assist them with inputting outcomes data into, and extracting it out of, their electronic system	Fliss (PI) and Assem (Data Analyst)	Practical and technical advice via face-to-face site visits, email and telephone	At sites/electronically	Upon request from site	Site specific support for:<br>- Inputting<br>- Viewing and using<br>- Extraction<br>- Submission of routine outcomes data to RESOLVE team	To complete retrospectively	To complete retrospectively
**Reporting and feedback**	Motivating and reinforcing the use of outcome measures through providing sites with meaningful feedback of their outcomes data. This can then be used to demonstrate how what they do as individuals/teams/organisations impacts patient care and outcomes	***Materials and procedures:*** Producing site based reports and graphs on outcomes data through our Registry protocol and feeding this back to each site.	Fliss (PI) and Assem (Data Analyst)	Electronic feedback via email	Electronic	Twice yearly	Site-specific support for:<br>- Producing outcomes reports<br>- Using outcomes data to inform practice at patinet, team, and service levels	To complete retrospectively	To complete retrospectively
**Running through general and site-specific implementation strategies**									
**Quality improvement facilitator (QIF)**	To champion the implementation of PCOMs at sites	Liase and work work sites locally in driving the implementation of PCOMs into practice, identifying and responding to local challenges/needs, keeping PCOMs on the agenda, and acting as a direct link between the research team and sites.	QIF (Martina)	Face-to-face support, email and telephone correspondence	At site	Throughout the RESOLVE implementation strategy	All support from the WIF will be tailored to local site needs and challenges	To complete retrospectively	To complete retrospectively

For peer review only

Version 1.0 08/07/2019

Data item specification

Note: Whilst we plan on collecting data items on dates (e.g., date of referral, first contact data, date ready for care etc.), these will be pseudonymised in Excel by participating sites prior to being submitted to the Registry through the formula below.

Before pseudonymising dates, please make sure that there are no cases from before the date requested. For example, if data is requested from June onwards, disregard any cases with a starting episode date before June.

1. Excel handles dates numerically in the form of 'serial numbers', with each date being represented by a specific serial number. For example, the serial number for the date 24/10/2019 is '42667'. In pseudonymising dates, therefore, each site will use Excel to convert each date (dd/mm/yy) into its serial number through formatting cells into ‘general’ instead of ‘date’.

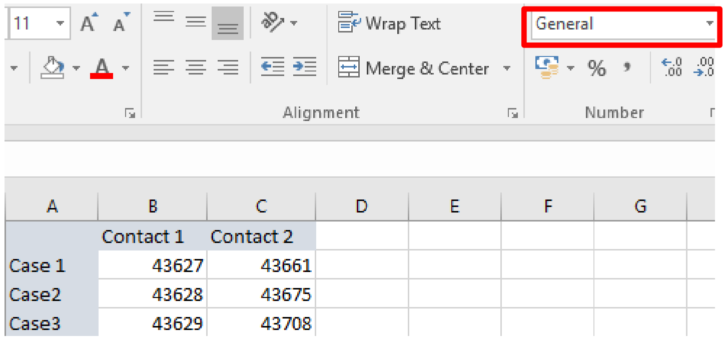

2. Hospices will then select and consistently use a random number between 1-500 (that the research team will not be made aware of) and add this to the serial number before submitting it to the registry.

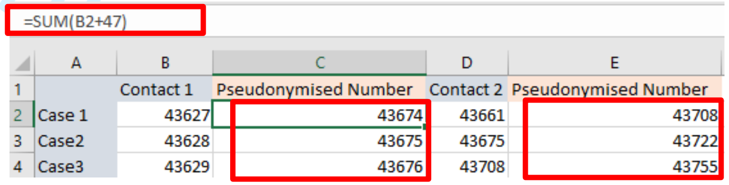

3. This will allow dates to be pseudonymised without the research team being able to trace back to the original date. The numbers that are produced can then be used to determine temporal factors of care that we are interested in(e.g., length of stay, time from referral to first contact) but without receiving dates which may identify participants.

For peer review only - http://bmjopen.bmj.com/site/about/guidelines.xhtml

Version 1.0 08/07/2019

Level 1: Patient Details – reported at patient level

No.	Variable Name	Definition	Format/Document	Use
1	Site ID	Unique identifier for each provider	A two letter and two digit code issued by the RESOLVE team (e.g., AA00)	To identify what palliative care provider is submitting data to the registry
2	Pseudonymised Unique Patient ID	A unique and pseudonymised patient identifier within the service's IT system that is used to identify the patient that is being cared by service providers.	This will be created by applying a macro to the NHS number, which provides a unique code which cannot be use to reverse to NHS number	To ensure the positive identification of a patient and that each individual's health records are associated with that individual and no other, while also enabling identification of repeat episodes of care for any one individual.
3	Age	The age of a patient at the start of their first episode of care	Age (in years old)	To determine the age of individual patients for conducting demographic analyses.
4	Gender	The gender of a patient	One of the following:<br>• Male<br>• Female<br>• Other<br>• Unknown	To determine gender of individual patients in a standardized format so that data can be used for related to service utilization, service needs, and epidemiological studies
5	Ethnicity	The ethnicity of the patient	One of the following:<br>• Mixed/multiple ethic groups<br>• Asian/Asian British<br>• Black/African/Caribbean/Black British<br>• Other ethnic group<br>• White	To determine ethnicity of individual patients in a standardized format so that data can be used for related to service utilization, service needs, and epidemiological studies
6	Primary Diagnosis	The primary life-limiting illness responsible for the patient receiving palliative care	Options showed in appendix 1 (ICD10 classification)	To determine a patients primary diagnosis.
7a	Secondary Diagnosis 1	Any secondary conditions / co-morbidities the patient has.	Options showed in appendix 1 (ICD10 classification)	To determine a patients secondary diagnosis.
7b	Secondary Diagnosis 2			
7c	Secondary Diagnosis 3			
8	Lives alone?	Whether or not the patient lives alone.	One of the following options:<br>• Yes	To understand a patients living arrangements for analysis (e.g., impact of living arrangements of cost of care and other outcomes)

For peer review only

Version 1.0 08/07/2019

No.	Variable Name	Definition	Format/Document	Use
			• No<br>• Unknown	
9	Usual Place of Residence	The patient's current main or permanent place of residence	One of the following:<br>• Patient's own home<br>• Other Private Residence (e.g., Relative's/Carers home)<br>• Care Home (residential/nursing facility)<br>• Hospice (inpatient specialist palliative care)<br>• Hospital<br>• Homeless<br>• Traveler<br>• Other	To understand place of care
10	Marital Status	A patient's marital status.	One of the following options:<br>• Single<br>• Married/Civil Partner/Cohabiting<br>• Divorced/Person whose Civil Partnership has been dissolved<br>• Widowed/Surviving Civil Partner<br>• Separated<br>• Not disclosed<br>• Unknown	To understand a patients marital status for analysis

For peer review only

Version 1.0 08/07/2019

Level 2: Episode Details – reported at episode level

No.	Variable Name	Definition	Format/Document	Use
11	Palliative episode number	The number episode that a patient is in	The corresponding number with a patient's episode (e.g., 1 for their first episode of care, 2 for their second, 3 for their third etc.)	To identify the number episodes a patient has experienced.
12	Referral Date	The date that a service receives a referral to provide palliative care for a patient for this episode	Date of referral (DD/MM/YYYY)	This will serve as a **key benchmarking variable**; the measurement of the time between referral and other variables (e.g., first contact date, date ready for care, episode start date). ***This date will be pseudonymised using the formula outlined at the start of this document.**
13	Primary Reason for referral	The primary reason for referral to specialist palliative care, as identified by person who refers patient.	One of the following options:<br>• Pain<br>• Other symptom control<br>• Emotional/psychological/spiritual support (patient)<br>• Emotional/psychological/spiritual support (family)<br>• Social/financial support (patient)<br>• Social/financial support (family)<br>• Rehabilitation<br>• Discharge planning<br>• Care in last days of life<br>• Respite<br>• Other	For analyses regarding why patients are referred to specialist palliative care services
14	Referral Source	The facility/organization which the patient was referred from for the start of a particular episode.	One of the following:<br>• Hospital – NHS<br>• Hospital – Outpatient<br>• Hospice – Inpatient<br>• Hospice – Day services<br>• Hospice – Outpatient<br>• Community – GP<br>• Community – Hospice at home<br>• Community – Out of hours services	To assist in understanding referral patterns and service planning.

For peer review only

Version 1.0 08/07/2019

No.	Variable Name	Definition	Format/Document	Use
			• Community – District/Community matron<br>• Self-referral/family-referral<br>• Internal referral within organisation<br>• Other (please state)	
15	First Contact Date	The date the clinical team makes contact with patient to assess needs after referral	Date of first clinical contact (DD/MM/YYYY)	This is a **key benchmarking variable** as it can be used to measure response time. ***This date will be pseudonymised using the formula outlined at the start of this document.**
16	Date Ready for Care	The date the patient is ready and available to receive palliative care. This may be determined through referral or first contact with patient	Date identified that patient is ready to receive palliative care (DD/MM/YYYY)	**Key benchmarking variable** as it is used to identify and account for early referrals (i.e., when a referral is received by a patient is not ready for care). ***This date will be pseudonymised using the formula outlined at the start of this document.**
17	Episode Start Date	The date of the first face-to-face palliative care assessment, using the set of core outcomes listed above. This is when clinical care starts	The date when clinical care starts (DD/MM/YYYY)	This date (along with episode end date) will be used to determine the length of stay for each episode of care. ***This date will be pseudonymised using the formula outlined at the start of this document.**
18	Care Setting	The setting/location where a patient is overnight.	One of the following:<br>• Hospital – Inpatient<br>• Hospital – Outpatient<br>• Hospice – Inpatient<br>• Community – Outpatient<br>• Community - Home visiting<br>• Community – Day care or day service<br>• Community – Nursing/residential home<br>• Community – Patient's/Carer's own home<br>• Community – Community hospital<br>• Other (please state)	To determine where an episode of care takes place
19	Team delivering care	The team delivering care within setting	Provided by the reporting organisation	Each organisation has different 'labels' for their teams (such as different clinics, therapy teams etc), yet may require sub-analysis by team to support effective quality improvement – we will therefore allow each reporting organisation to self-specify this variable
20	Episode end date	The date when a patient moves from one setting of care to another, dies, or	Date of episode end (DD/MM/YYYY)	To use with episode start date to determine the length of care for each episode. ***This date will be pseudonymised using the formula outlined at the**

For peer review only

Version 1.0 08/07/2019

No.	Variable Name	Definition	Format/Document	Use
		is no longer receiving palliative care		**start of this document.**
21	Episode result	The reason that an episode of palliative care ended	One of the following:<br>• Discharged to own home (or relatives/carers home)<br>• Discharged to nursing/residential home<br>• Discharged to hospital<br>• Discharged to NHS hospice<br>• Discharged to non-NHS hospice<br>• Died<br>• Unknown	Describes how the episode ended, determines number of deaths, and discharge locations.
22	Preferred place of death (if relevant)		One of the following:<br>• Nursing/residential home<br>• Home<br>• Hospice<br>• Hospital<br>• Other	
23	Whether patient is now deceased		• Yes<br>• No<br>• Unknown	Reporting may occur some time after episodes of care are completed. Knowing if the patient subsequently died will enable reporting of how early in the trajectory of illness patients are being seen.
24	Date of death		Date of death (DD/MM/YYYY)	This date will be used to determine at what point in the illness trajectory, the episode of care was delivered. ***This date will be pseudonymised using the formula outlined at the start of this document.**
25	Place of death (if relevant)		One of the following:<br>• Nursing/residential home<br>• Home<br>• Hospice<br>• Hospital<br>• Other	

For peer review only

Version 1.0 08/07/2019

Level 3: Phase Details – reported at contact level

No.	Variable Name	Definition	Format/Document	Use
26	Palliative Phase Number	The number phase that a patient has within an episode.	The corresponding number with a patient's phase (e.g., 1 for their first phase of illness, 2 for their second, 3 for their third etc.)	To identify the number phases a patient has experienced within an episode and to work out number of patient contacts.
27	Palliative Phase of Illness	Stage of a patient's illness, assessed at the start of the phase as assessed by Phase of Illness questionnaire (see appendix X)	One of the following:<br>• Stable<br>• Unstable<br>• Deteriorating<br>• Dying<br>• Deceased<br>• Bereavement/post death support<br>• Unknown (see appendix X for phase definitions)	To identify the stage of a patient's illness
28	Phase Start Date	The date each new phase of illness begins	Phase start date (DD/MM/YYYY)	When used with phase end date, can tell us the length of a phase of illness. ***This date will be pseudonymised using the formula outlined at the start of this document.**
29	Phase End Date	The date each phase of illness ends	Phase end date (DD/MM/YYYY)	When used with phase start date, can tell us the length of a phase of illness. ***This date will be pseudonymised using the formula outlined at the start of this document.**
30	Is this the End of a Phase?	Whether the patient has reached the end of a phase of illness.	One of the following:<br>• Yes<br>• No	To clarify the end of a phase of illness
31	Has the Phase Changed?	To highlight whether a patient has undergone a change in their phase of illness.	One of the following:<br>• Start Phase<br>• Continuing Phase<br>• End Phase	To cross check with phase of illness assessments
32	Contact Date	The date a health-care professional has clinical contact with patient.	DD/MM/YYYY.	To align contact dates – and clinical data collected at these - with changes in episodes and phases. ***This date will be pseudonymised using the formula outlined at the start of this document.**
33	Australia-Modified Karnofsky Performance	An assessment a patient's functional status at the beginning of a phase of illness	Functional status, as measured by AKPS ranging from 0-100% in 10% increments, (where 100% is fully functioning and 10% is unarousable). See	**Key benchmarking variable**. An analysis of functional status at phase start

For peer review only

Version 1.0 08/07/2019

No.	Variable Name	Definition	Format/Document	Use
	Scale (AKPS) Score		appendix X for scale	
34	Integrated Patient Outcomes Scale (IPOS) at	An assessment of a patient's symptoms and other concerns at phase start (see items between numbers X-X)	Inputting data from each item of IPOS (see appendix X)	**A key benchmarking variable**. A measure of a patient's perceptions of their symptoms at the start of a phase of illness
35	Barthel Activities of Daily Living	To assess patient's activities of daily living at phase start	Inputting data from each item of BARTHEL (see appendix X)	
36	Views on Care (VOC)	To assess a patient's ratings of their quality of life, overall well-being, and their view of the impact of the service on their problem(s) at the start of a phase of illness	Inputting data from each item of VOC (see appendix X)	To measure a patients views on care

For peer review only

Version 1.0 08/07/2019

Appendix 1: ICD10 Classifications

No.	RESOLVE Diagnoses Groups
1	Cancer - Cancer of bone, skin, mesothelial and soft tissue, thyroid or endocrine [C40-C49 & C73-C75]
2	Cancer - Cancer of breast [C50]
3	Cancer - Cancer of independent multiple sites [C97]
4	Cancer - Cancer of liver, intrahepatic bile ducts, gallbladder [C22-C24]
5	Cancer - Cancer of respiratory & intrathoracic organs, including lung (C30-C39)
6	Cancer - Cancer of the digestive organs, including colon, rectum, stomach, excluding liver, GB, pancreas [C15-C21 & C26]
7	Cancer - Cancer of the eye, brain and other CNS [C69-C72]
8	Cancer - Cancer of the female genital organs [C51-58]
9	Cancer - Cancer of the lip, oral cavity & pharynx [C00-C14]
10	Cancer - Cancer of the male genital organs, including prostate [C60-C63]
11	Cancer - Cancer of the pancreas [C25]
12	Cancer - Cancer of the urinary tract [C64-C68]
13	Cancer - Cancer of unknown primary or other unspecified [C76-C80]
14	Cancer - Lymphoid & haematopoietic cancer [C81-C96]
15	Multiple non-cancer conditions - addition to help with multi-morbidity
16	Non-cancer - All other non-cancer diagnoses
17	Non-cancer - Chronic renal failure [N18]
18	Non-cancer - Chronic respiratory disease [J40-J70]
19	Non-cancer - Dementia including Alzheimer's disease [G30 & F00-F03]
20	Non-cancer - Diabetes mellitus [E10-E14]
21	Non-cancer - Heart failure [I50]
22	Non-cancer - HIV disease/AIDS [B20-B24]
23	Non-cancer - Liver failure, chronic liver disease, other non-malignant liver disease [K70-K77]
24	Non-cancer - Motor neurone disease [G12]
25	Non-cancer - Neurological conditions (excluding MND and Alzheimer's) [G00-G99

For peer review only

Version 1.0 08/07/2019

No.	RESOLVE Diagnoses Groups
	excluding G12 & G30]
26	Non-cancer - Other heart & circulatory conditions excluding heart failure and stroke [I00-I99 excluding I50, I61, I63]
27	Non-cancer - Stroke, infarction or haemorrhagic [I61 & I63]
28	Unknown

For peer review only

Version 1 (15/12/2020)

IMPLEMENTING PERSON-CENTRED OUTCOMES MEASURES INTO PALLIATIVE CARE

Information about this survey

What is this survey about?

This survey is part of a study called 'RESOLVE'. RESOLVE is a research programme aimed at facilitating the implementation of Person-Centred Outcome Measures into routine palliative care. This is so that, as a palliative care community, we ensure regular assessment and monitoring of symptoms and other concerns of those receiving palliative care. As part of our project we have developed an intervention strategy, and this survey is one way in which we are trying to understand if it is effective at facilitating the integration of outcome measures into everyday practice. More information on the RESOLVE project can be found through our website: https://www.hyms.ac.uk/research/research-centres-and-groups/wolfson/resolve.

Why have I been chosen?

You have been selected to complete this survey as you are a staff member at a RESOLVE-affiliated site that has received the RESOLVE implementation strategy. Sites with the best completion rate will receive a voucher for a textbook or one-year journal subscription.

Do you consent to taking part in this survey?

☐ Yes ☐ No

What about my data?

By completing and submitting this survey you are providing consent for your responses to be used as part of the RESOLVE research project. All data is anonymised and confidential and will be stored in accordance with General Data Protection Regulation (GDPR). If you have any questions, please contact the RESOLVE team (resolve@hyms.ac.uk).

Survey Instructions

This survey is split into 3 parts and should take no longer than 15 minutes to complete. Part A asks some brief questions about yourself and your role. Part B includes questions about what outcome measures you use and how you use them to inform your everyday practice. Part C asks more detailed questions about PCOMs.

For peer review only - http://bmjopen.bmj.com/site/about/guidelines.xhtml

Please take the time to decide which answer **best suits your experience for each statement and tick the appropriate box**

Part A: About yourself

➢ What is your professional role?

☐Nurse ☐Doctor ☐Allied health professional ☐Healthcare assistant ☐Chief exec/manager ☐ I.T. or data ☐Other (dropdown box)

➢ What is the name of your organisation?

☐ Dove House Hospice ☐ Marie Curie Bradford Hospice ☐ Wheatfields Hospice ☐ St Gemma's Hospice ☐ St Andrew's Hospice
☐ Grimsby Care Plus Group ☐ Kirkwood Hospice ☐ St Luke's Hospice ☐ St Catherine's Hospice
☐ City Health Care Partnership Hull ☐ Bradford District Care Trust ☐ York Teaching Hospital NHS Foundation Trust

➢ What is your age?

☐ 18-24 ☐ 25-34 ☐ 35-44 ☐ 45-54 ☐ 55+ ☐ Prefer not to say

➢ What is your gender?

☐ Male ☐ Female ☐ Other ☐ Prefer not to say

➢ By which ethnicity do you identify?

☐ White ☐ Black/African/Caribbean/Black British ☐ Asian/Asian British ☐ Mixed/multiple ethic groups ☐ Other
☐ Prefer not to say

➢ What setting do you work in (tick multiple if you work across settings)?

☐ In-patient hospice ☐ Outpatient/day therapy ☐ Community ☐ Hospital

➢ How long have you worked in palliative care?

☐ Less than a year ☐ 1-5 years ☐ 6-10 years ☐ 11-15 years ☐ 15+ years

➢ From the statements below, please choose an option that best describes ***your main role*** in relation to outcome measures:

☐ I am involved in managing or overseeing the implementation and use of outcome measures
☐ I am involved in using outcome measures as part of my everyday practice
☐ I use outcome measures occasionally as part of my everyday practice
☐ I am not using outcome measures as part of my everyday practice

For peer review only

For peer review only - http://bmjopen.bmj.com/site/about/guidelines.xhtml

Part B: What outcome measures do you use?

Outcome measures are short questionnaires that provide healthcare professionals with information on a person's own perception of their health status and well-being. They include palliative Phase of Illness, Integrated Palliative care Outcome Scale (IPOS), and Australia-modified Karnofsky Performance Scale (AKPS). This survey relates to your use of these outcome measures.

- Do you use the following outcome measures?

	At 1st assessment	At follow-up
palliative Phase of Illness	☐ Yes ☐ No	☐ Daily ☐ Weekly ☐ At clinical assessment<br>☐ Other (open free text response box)

	At 1st assessment	At follow-up
Integrated Palliative care Outcome Scale (IPOS)	☐ Yes ☐ No	☐ Yes ☐ No
Australia-modified Karnofsky Performance Scale (AKPS)	☐ Yes ☐ No	☐ Yes ☐ No

- At what follow up interval do you collect IPOS and AKPS data?

  ☐ At change of palliative Phase of Illness ☐ End of episode of care ☐ Other (open free text response box)

- Do you or your team use these measures to shape the care of individual patients, including in multi-disciplinary team meetings?

- Do you or your team use these measures to shape the services that your team or organisation delivers?

For peer review only

Part C: Detailed questions about outcome measures

These questions are designed to help us find out more about whether people and teams understand what outcome measures are and how they may be used during practice. For each statement please select an answer that best suits your experience.

**Section C1**	Strongly agree	Agree	Neither agree nor disagree	Disagree	Strongly disagree	Not relevant to my role	Don't know
**I can see how outcome measures differs from usual ways of working**	☐	☐	☐	☐	☐	☐	☐
**Palliative care staff in this organisation have a shared understanding of the purpose of outcome measures**	☐	☐	☐	☐	☐	☐	☐
**I understand how using outcome measures affects the nature of my own work**	☐	☐	☐	☐	☐	☐	☐
**I can see the potential value of outcome measures**	☐	☐	☐	☐	☐	☐	☐

- Please feel free to expand on your responses below

These questions are designed to help us find out more about how everyday practice is built around using outcome measures and how you think they relate to your role. For each statement please select an answer that best suits your experience.

**Section C2**	Strongly agree	Agree	Neither agree nor disagree	Disagree	Strongly disagree	Not relevant to my role	Don't know
**There are key people who drive the use of outcome measures and get others involved**	☐	☐	☐	☐	☐	☐	☐
**I believe that using outcome measures is a legitimate part of my role**	☐	☐	☐	☐	☐	☐	☐
**I am open to working with colleagues in new ways to use outcome measures**	☐	☐	☐	☐	☐	☐	☐
**I will continue to support the use of outcome measures**	☐	☐	☐	☐	☐	☐	☐

- Please feel free to expand on your responses below

For peer review only

These questions are designed to help us better understand the ways in which you experience using outcome measures as part of your role. For each statement please select an answer that best suits your experience.

**Section C3**	Strongly agree	Agree	Neither agree nor disagree	Disagree	Strongly disagree	Not relevant to my role	Don't know
**I can easily integrate outcome measures into existing work**	☐	☐	☐	☐	☐	☐	☐
**Outcome measures disrupt working relationships**	☐	☐	☐	☐	☐	☐	☐
**I have confidence in other people's ability to use outcome measures**	☐	☐	☐	☐	☐	☐	☐
**Work is assigned to those with skills appropriate to using outcome measures**	☐	☐	☐	☐	☐	☐	☐
**Sufficient training is provided to enable palliative care staff to implement and use outcome measures**	☐	☐	☐	☐	☐	☐	☐
**Sufficient resources are available to support outcome measures use**	☐	☐	☐	☐	☐	☐	☐
**Management adequately supports the use of outcome measures**	☐	☐	☐	☐	☐	☐	☐

- Please feel free to expand on your responses below

For peer review only

These questions are designed to help us understand the ways in you appraise the value of outcome measures. For each statement please select an answer that best suits your experience.

**Section C4**	Strongly agree	Agree	Neither agree nor disagree	Disagree	Strongly disagree	Not relevant to my role	Don't know
**I am aware of reports about the effects of outcome measures on patient care**	☐	☐	☐	☐	☐	☐	☐
**The palliative care staff agree that using outcome measures is worthwhile**	☐	☐	☐	☐	☐	☐	☐
**I value the effects that outcome measures have had on my work**	☐	☐	☐	☐	☐	☐	☐
**Feedback about outcome measure data can be used to improve how they are used in future**	☐	☐	☐	☐	☐	☐	☐
**I can modify how I work with outcome measures**	☐	☐	☐	☐	☐	☐	☐

- Please feel free to expand on your responses below

For peer review only

- Are you happy to be contacted to complete this survey again (in around 8-12 months' time)? This is so that we are able to explore the impact of the RESOLVE implementation strategy over time.

  ☐ Yes (if selected then include dropdown box that asks for email address so that we are able to contact, alongside statement that we will send them a certificate of participation for this survey)

  ☐ No (if selected then include dropdown box that asks if respondent wants to receive a certification of participation. If they do, prompt for email address)

Thank you very much for completing this survey.

What happens next?

This data will be stored confidentially and analysed as part of the RESOLVE project to understand the ways in which the RESOLVE implementation strategy has (or has not) worked to help implement outcome measures into routine clinical practice. This data will be used as part of reports, publications, and conference presentation. All information included within these outputs will be anonymised and it will not be possible for others to identify you. If you have any questions, please contact the RESOLVE team (resolve@hyms.ac.uk).

For peer review only

Version 1 (15/12/2020)

Introduction to the Interview

Thank you very much for volunteering to be interviewed for the RESOLVE project. The aim of this project is to implement Person-Centred Outcome Measures (PCOMs) into routine palliative care across Yorkshire.

As part of RESOLVE, we have developed an implementation strategy to facilitate the implementation of PCOMs into practice. This strategy was developed based on what health professionals' thought were important challenges that needed to be addressed in order to integrate PCOMs into routine practice. Our strategy includes:

- Educational resources
- Workshop and conference events
- Determining organisational and team needs
- Formal and informal training
- IT support
- Reporting and feedback
- Quality improvement facilitator

The purpose of this discussion is to understand your thoughts and feelings on the extent to which you think this implementation strategy has been effective at enhancing the uptake and routine use of PCOMs in everyday practice. It should take around 30 minutes.

Do you have any questions before we start?

The first few questions are around what measures you currently use and how you use them.

- Are you able to tell me what palliative care outcome measures you use in your everyday practice? (probe for use of the three core measures: palliative Phase of Illness, AKPS, and IPOS)
- When were these measures introduced? (probe for when each of the three core was introduced)
- How do you use these measures in practice? Probe for:
  - Who collects these measures?
  - How often they are collected/timings?

Version 1 (15/12/2020)

- Which version is used (largely version of IPOS, but also version of Barthel if mentioned)?

- Has your use of outcome measures changed over the last 1-2 years?
  - Collecting?
  - Scoring?
  - Reporting?
  - General understanding of each measure (i.e., what they are, how to use them, their value)?
- Do you think that the way in which you currently use outcome measures is better or worse than previously?
  - What has changed or remained the same?
  - Why?

The next few questions focus on the ways in which you may have used different components of the RESOLVE implementation strategy and how, if at all, you feel as though it may have impacted how you use outcome measures.

- Have you, or your team/service, used any of the RESOLVE implementation strategies
  - Educational resources
  - Workshop and conference events
  - Determining organisational and team needs
  - Formal and informal training
  - IT support
  - Reporting and feedback
  - Quality Improvement Facilitator, or other RESOLVE team members

For peer review only

Version 1 (15/12/2020)

- Can you comment on the extent to which you feel the RESOLVE implementation has been put into practice within your service?
    - Which elements?
    - How much?

Coherence

- What do outcome measures mean to you in relation to your role?
    - Have these views changed over time?
    - Why?
- Do you feel as though the RESOLVE implementation strategy has changed your own (and other colleagues') understandings of what outcome measures are and how to use them?
    - On scoring consistently and confidently?
    - On what they mean (especially Phase of Illness)?
    - Frequency of collection (and selecting the relevant versions)?
- How and why do you think these things?

Cognitive Participation

- Are you able to reflect on the ways in which the RESOLVE implementation strategy may have contributed to building clinical/team practice around outcome measures?
    - Has it influenced communicating with outcome measures in relation to individual patients (i.e., ward rounds, MDT's, handovers etc.)?
    - How?
    - Has it shaped the services that your team or organisation delivers?
    - How?

For peer review only

Version 1 (15/12/2020)

Collective Action

- Thinking back over the last year, has the way in which you use outcome measures (both individually and as part of your team) changed as a result of the RESOLVE implementation strategy?
    - How?
    - What is different now?
    - What parts of the RESOLVE implementation strategy do you feel are responsible for these changes?
- Has the implementation strategy helped to develop your own, and your teams, skills, and confidence in using outcome measure to inform care at a patient/team/service level?
    - What has helped with this?
- Were there any organisational barriers to using outcome measures (individually and as part of your team) that the RESOLVE implementation strategy has helped to address?
    - What were these?
    - How/why did the implementation strategy help?
- Has your confidence to confidently/efficiently input, share, and extract outcomes data within your electronic systems changed?
    - If any, what part of the RESOLVE implementation strategy has been key to this?

Reflexive Monitoring

- Are you able to reflect on the ways in which the RESOLVE implementation strategy has changed your views on how you value outcome measures in everyday practice?

For peer review only

Version 1 (15/12/2020)

  - How?
  - Why?

- How do you share feedback on outcome measures?
  - Do you feel as though the RESOLVE implementation strategy has had an impact on for you/your team/the organisation to do this?
  - How/Why?

- Are there any aspects of the RESOVLE implementation strategy that you think have contributed/will contribute to sustained use of outcome measures?
  - What are these?
  - Why?
  - How will use be sustained once the RESOLVE project is over?

Thank you for taking part in this interview. Before we finish, do you have any other questions?

For peer review only

BMJ Open

Implementing Person-Centred Outcome Measures (PCOMs) into routine palliative care: Protocol for a mixed-methods process evaluation of the RESOLVE PCOM Implementation Strategy

Journal:	*BMJ Open*
Manuscript ID	bmjopen-2021-051904.R1
Article Type:	Protocol
Date Submitted by the Author:	06-Jul-2021
Complete List of Authors:	Bradshaw, Andy; University of Hull<br>Santarelli, Martina; University of Hull<br>Khamis, Assem M; Hull York Medical School<br>Sartain, Kathryn; University of Hull; York and Scarborough Teaching Hospitals NHS Foundation Trust<br>Johnson, Miriam; The University of Hull, Hull York Medical School<br>Boland, Jason; Hull York Medical School<br>Pearson, Mark; Hull York Medical School, Wolfson Palliative Care Research Centre<br>Murtagh, Fliss; Wolfson Palliative Care Research Centre, Hull York Medical School, University of Hull, UK
**Primary Subject Heading**:	Palliative care
Secondary Subject Heading:	Evidence based practice, Patient-centred medicine, Qualitative research, Research methods
Keywords:	PALLIATIVE CARE, Protocols & guidelines < HEALTH SERVICES ADMINISTRATION & MANAGEMENT, Adult palliative care < PALLIATIVE CARE

SCHOLARONE™
Manuscripts

I, the Submitting Author has the right to grant and does grant on behalf of all authors of the Work (as defined in the below author licence), an exclusive licence and/or a non-exclusive licence for contributions from authors who are: i) UK Crown employees; ii) where BMJ has agreed a CC-BY licence shall apply, and/or iii) in accordance with the terms applicable for US Federal Government officers or employees acting as part of their official duties; on a worldwide, perpetual, irrevocable, royalty-free basis to BMJ Publishing Group Ltd ("BMJ") its licensees and where the relevant Journal is co-owned by BMJ to the co-owners of the Journal, to publish the Work in this journal and any other BMJ products and to exploit all rights, as set out in our licence.

The Submitting Author accepts and understands that any supply made under these terms is made by BMJ to the Submitting Author unless you are acting as an employee on behalf of your employer or a postgraduate student of an affiliated institution which is paying any applicable article publishing charge ("APC") for Open Access articles. Where the Submitting Author wishes to make the Work available on an Open Access basis (and intends to pay the relevant APC), the terms of reuse of such Open Access shall be governed by a Creative Commons licence – details of these licences and which Creative Commons licence will apply to this Work are set out in our licence referred to above.

Other than as permitted in any relevant BMJ Author's Self Archiving Policies, I confirm this Work has not been accepted for publication elsewhere, is not being considered for publication elsewhere and does not duplicate material already published. I confirm all authors consent to publication of this Work and authorise the granting of this licence.

For peer review only

Title: Implementing Person-Centred Outcome Measures (PCOMs) into routine palliative care: Protocol for a mixed-methods process evaluation of the RESOLVE PCOM Implementation Strategy

Bradshaw, A [1]., Santarelli, M [1]., Khamis, A [1]., Sartain, K [1,2]., Johnson, M. J [1]., Boland, J. W [1]., Pearson, M [1]., Murtagh, F.E.M [1].*

[1] *Wolfson Palliative Care Research Centre, Hull York Medical School, University of Hull, Hull, UK*

[2] *York and Scarborough Teaching Hospitals NHS Foundation Trust, York, UK*

*Corresponding author: Andy Bradshaw, Allam Medical Building, University of Hull, Hull, HU6 7RX. Email: andrew.bradshaw@hyms.ac.uk

Word count (excluding title page, abstract, references, figures and tables): 4,052

For peer review only - http://bmjopen.bmj.com/site/about/guidelines.xhtml

Abstract

Introduction: Person-centred outcome measures improve quality of care and patient outcomes but are used inconsistently in palliative care practice. To address this implementation gap, we developed the 'RESOLVE Implementation Strategy'. This protocol describes a process evaluation to explore mechanisms through which this strategy does, or does not, support the implementation of outcome measures in routine palliative care practice.

Methods and analysis: Multistrand, mixed methods process evaluation. Strand one will collect routine outcomes data (palliative Phase of Illness, Integrated Palliative care Outcomes Scale, Australia-modified Karnofsky Performance Status) to map the changes in use of outcome measures over 12 months (July 2021-July 2022). Strand two will collect survey data over the same 12-month period to explore how professionals' understandings of, skills in using, and ability to build organisational practices around, outcome measures change over time. Strand three will collect interview data to understand the mechanisms underpinning/affecting our implementation strategy. Thematic Framework analysis and descriptive statistics will be used to analyse qualitative and quantitative data, respectively.

Ethics and dissemination: For strand one, ethical approval has been obtained (Cambridge REC, REF: 20/EE/0188). For strands two and three ethical approval has been obtained from Hull York Medical School ethics committee [21 05]. Tailored feedback of study findings will be provided to participating sites. Abstracts and papers will be submitted to national/international conferences and peer-reviewed journals. Lay and policy briefings and newsletters will be shared through PPI and project networks, plus via the project website.

Keywords: Implementation science; Outcome measures; Palliative care; Process evaluation

For peer review only

For peer review only - http://bmjopen.bmj.com/site/about/guidelines.xhtml

Strengths and limitations of this study

- This is the first process evaluation of an implementation strategy designed to facilitate the integration of Person-Centred Outcome Measures into routine palliative care practice.
- This protocol follows the Medical Research Council's guidance on process evaluations by adopting a systematic and theoretically informed approach to study design and conduct.
- We will adopt a multi-strand, mixed methods approach in which we will integrate routine outcomes, survey, and focused interview data to demonstrate the mechanisms through which our intervention strategy may or may not work across different settings of palliative care.
- Since we only focus on implementing a core set of outcome measures, the applicability of findings may be limited to other outcome measures, many of which have measure-specific challenges to implementation.

For peer review only - http://bmjopen.bmj.com/site/about/guidelines.xhtml

INTRODUCTION

Person-Centred Outcome Measures in Palliative Care

Palliative care aims to alleviate suffering due to progressive, life-limiting medical conditions and improve quality of life through the adoption of a holistic, person-centred, and multidisciplinary approach that is delivered across multiple settings (in-patient, outpatient, hospital, and community).[1] It is projected that by 2060, the global need for palliative care will double (from 26 million to 48 million people).[2] It is important, therefore, that we are able to clearly demonstrate the quality of palliative care services by showing if and how they are able to improve a person's symptoms, and sustain or improve wellbeing and functional ability. To do this, focusing on outcomes is essential.

In a healthcare context, an outcome refers to 'the change in a patient's current and future health status that can be attributed to preceding healthcare'.[3] Within palliative care, measuring outcomes is important because they allow us to evaluate whether the care that is given to patients and their families makes a difference to their quality of life. [4] In capturing this information, Person-Centred Outcome Measures (PCOMs) are considered the 'gold standard' for outcome measurement in palliative care. [5, 6] These are standard, validated questionnaires – usually filled in by patients themselves - that provide healthcare professionals with important information on a person's own perception of their health status and well-being. [7] Moreover, because many patients with life-limiting illnesses may have impaired cognition and/or be too unwell to fill out PCOMs on their own accord, [8] PCOMs may sometimes be completed using proxy-reported ratings (e.g., by healthcare professionals and/or a patient's family member).

Current evidence demonstrates the value of PCOMs in palliative care in helping to identify (often unrecognised), continually monitor, and take direct clinical action to address, a

For peer review only - http://bmjopen.bmj.com/site/about/guidelines.xhtml

person's needs/symptoms; facilitate communication between patients and clinicians; and improve outcomes. [4, 7, 9] This is through using PCOMs at individual/patient (e.g., for clinical assessment), team (e.g., focusing patient reviews and workload planning), service (e.g., service evaluation/development and making business cases), and population (e.g., benchmarking) levels of practice. [10] For these reasons, the European Association for Palliative Care Task Force on Outcome Measurement recommended that PCOMs should be firmly embedded into routine clinical practice across all settings in which palliative care is delivered. [6]

Despite the evidence underpinning the value of using PCOMs, they are used inconsistently in clinical practice, if at all. Reasons for this include issues such as time constraints, lack of training and knowledge, tools being perceived as burdensome, negative attitudes towards outcome measures, inefficient electronic systems, top-down approaches to implementation, and fear of added work. [5, 11-13] In addressing these challenges and facilitating the implementation of PCOMs into everyday clinical practice, various studies have provided tables of recommendations to consider before and during implementation. [5, 12, 13] These have recommended focusing on individual, management, and organisational issues during the preparation and roll-out stages of implementation. To date, however, there has been no attempt to develop these into, and subsequently evaluate, an implementation strategy that may be applied across all palliative care settings.

The RESOLVE project

The RESOLVE project is a multi-site implementation and research programme aimed at implementing PCOMs into routine clinical practice in 11 specialist palliative care services across Yorkshire, England. These services vary in size, funding source (e.g., charitable/NHS), location (e.g., urban/semi-rural/rural), and span hospice inpatient, outpatient/day therapy, and home-based/community settings. As part of this study, we conducted an exploratory

For peer review only

qualitative study in which semi-structured interviews were used to understand the processes and causal mechanisms that underpin the successful implementation of PCOMs into routine practice. [13] Building on insights from this study about implementation and our collaborative work with sites, this study protocol describes a process evaluation of the complex intervention – named 'The RESOLVE PCOM Implementation Strategy' – aimed at facilitating the integration of PCOMs into routine practice. It is important to evaluate these types of interventions to understand what does (and does not work) and in what contexts.

Process evaluations are useful in understanding the ways in which complex interventions work because they provide insight into the causal mechanisms and contextual factors at play during implementation. [14] Currently, however, no process evaluations have been conducted that explore the impact of implementation strategies aimed to promote the uptake and sustained use of PCOMs within palliative care contexts.

Therefore, we aim to conduct a process evaluation that explores the mechanisms through which The RESOLVE PCOM Implementation Strategy does, or does not, support the implementation of PCOMs in routine palliative care practice. We aim to achieve this by answering three research questions:

1. Does The RESOLVE PCOM implementation strategy enhance the uptake and routine use of PCOMs?
2. Does The RESOLVE PCOM implementation strategy enhance health care professionals' individual and collective understanding of, skills in using, and ability to build organisational practices around, PCOMs?
3. If The RESOLVE PCOM implementation strategy is effective, through which mechanisms are its intended effects achieved?

For peer review only - http://bmjopen.bmj.com/site/about/guidelines.xhtml

METHODS AND ANALYSIS

Study design

A multistrand, mixed methods, longitudinal process evaluation informed by The Medical Research Council guidance. [15] To do this, we will adopt a systematic approach in which we provide clear descriptions of, and collect relevant data pertaining to: (i) our complex intervention; (ii) the implementation process; (iii) mechanisms; (iv) context; and (v) outcomes/results (see Table 1). This will be achieved through three strands of work. An overview these can be seen in Table 2.

Core PCOMs set

The three outcome measures we aim to implement are a nationally-recognised and validated core set of PCOMs for specialist palliative care. [16] These measures are based on what patients and their families prioritise as important in advanced illness and have been endorsed by NHS England and Public Health England. They include the palliative Phase of Illness, [17] Integrated Palliative care Outcomes Scale (IPOS), [18, 19] and Australia-modified Karnofsky Performance Status (AKPS). [20] More information about each of these measures can be seen in Table 3.

The implementation of these PCOMs is not simply about increased use, but the wrap-around work that is needed for the effective and appropriate use of these measures to improve care. This includes: organizational and team recognition of why using measures is important, using the right measures at the right time, ensuring follow-up assessments using the measures, getting all teams/team members on board, having efficient feedback systems in place, having all members of the team understanding the purpose of PCOMs, and embedding PCOMs into the 'cultural fabric' of how teams and services operate. [13]

For peer review only - http://bmjopen.bmj.com/site/about/guidelines.xhtml

Table 1: Key components of a process evaluation and how this study has considered these. Grey shaded areas represent key components of a process evaluation according to Medical Research Council guidance.

**Key components of process evaluations**	**Medical Research Council guidance [15]**	**This study**
**Describing the complex intervention and clarifying causal assumptions**	'A clear description of the intended intervention, how it will be implemented, and how it is expected to work, will ideally have been developed before evaluation'	We describe The RESOLVE Person-Centred Outcome Measure (PCOM) Implementation strategy and have drawn on Normalisation Process Theory to explain the causal assumptions through which we expect it to work (see pages 10-12)
**Implementation: what is implemented, and how?**	• 'Implementation process (How delivery is achieved; training, resources etc.)'<br>• 'What is delivered (fidelity, dose, adaptations, reach)'	We have outlined, in detail, the features of our complex interventions using the template for intervention description and replication (TIDieR) checklist (see supplementary file 1). This outlines what each component is, why we are doing it, who is delivering, how much they are delivering, where and when it is being delivered, how it may be tailored/modified, and how well it will be delivered (e.g., fidelity).
**Mechanisms of impacts**	• 'Participant responses to and interactions with the intervention'<br>• 'Mediators'<br>• 'Unexpected pathways and consequences'	In understanding mechanisms of impacts, we are collecting three types of data:<br>- Routinely collected Person-Centred Outcome Measures data (see pages 13-14).<br>- Survey data (see pages 14-17).<br>- Focused interview data (see page 17-19).
**Context**	• 'Contextual factors that shape theories of how the intervention works'<br>• 'Contextual factors that affect (and may be affected by) implementation, intervention mechanisms and outcomes'<br>• 'Causal mechanisms present within the context which act to sustain the status quo, or potentiate effects'	Analysis of focused interview data which provide insight into the contextual factors and mechanisms (e.g., settings of care or organisational characteristics) that affect (and are affected by) the implementation process. (see page 19).
**Outcomes**	Analysis of process data, and integration of process	We will combine results/analysis from quantitative and

For peer review only - http://bmjopen.bmj.com/site/about/guidelines.xhtml

	and outcome data	qualtiative analyses to produce numerous outputs that answer the research questions of this study. (see page 19-20).

For peer review only

For peer review only - http://bmjopen.bmj.com/site/about/guidelines.xhtml

Table 2: An overview of the research design for this study

Strand	Data collection method	Data collection timepoints	Reason/output
**1**	Routine outcomes	Baseline and follow-up	To map changes in use (i.e., implementation status) of Person-Centred Outcome Measures over time **(RQ 1)**
**2**	Survey	Initial piloting phase and then at baseline and follow-up	To determine how changes in Person-Centred Outcome Measures over time (RQ 1) use relates to changes in participants' coherence, engagement, action, and reflexive monitoring of measures **(RQ 2)**
**3**	Focused interviews	Towards end of project	To determine why? how? context? Of any changes (i.e., mechanisms) **(RQ 2&3)**

Table 3: Description of the core set of PCOMs that we aim to implement into routine palliative care practice as part of the RESOLVE project.

Measure	Description	Frequency/timing of collection
palliative Phase of Illness [17]	Palliative Phase of Illness is a measure which describes the urgency of care needs for a person receiving palliative care. It does so by describing four distinct phases of a patient's illness, including: stable, unstable, deteriorating, dying, and deceased. These Phases are measured through determining the care needs of a patient and/or their family and provide a clinical indication of a patient's Phase of Illness which can be used to inform care planning.	**Inpatients (hospice)**<br>- Daily<br><br>**Community**<br>- Each contact
Integrated Palliative care Outcomes Scale (IPOS) [18, 19]	A holistic, well-validated, and global measure of symptom burden that uses 10 questions (scored on a 0-4 Likert type scale) to assess the most important symptoms and concerns of patients affected by life-limiting illnesses across physical, psychological, social, and existential domains of well-being. There are two forms of IPOS; patient-IPOS (where patients complete the questionnaire as a self-report) and staff-IPOS (a proxy version which is completed by staff).	**Inpatients (hospice)**<br>- Initial assessment<br>- Change in Phase of Illness<br>- End of episode<br><br>**Community**<br>- Each contact
Australia-modified Karnofsky Performance Status (AKPS) [20]	Assesses a patient' overall performance/functional status across 3 dimensions: activity, work, and self-care. Healthcare professionals use their observations of patients' ability to perform everyday tasks and scores them at 10% increments between 0% (i.e., the patient is dead) and 100% (i.e., no complaints or evidence of disease).	

For peer review only

Description and causal assumptions of the RESOLVE PCOM implementation strategy

Description of complex intervention

Whilst all participating sites collect PCOMs in some form, their use is variable. For example, some use all three measures but not consistently across settings, others may use certain measures consistently in some settings but not others, whilst some are seeking to introduce new measures that they do not currently collect. In facilitating the implementation of PCOMs into routine practice in these instances, we have worked collaboratively with specialist palliative care services and health professionals to develop 'The RESOLVE PCOM Implementation Strategy'. This represents a complex intervention in that it 'comprise[s] [of] multiple interacting components' (see Table 4 for a detailed breakdown of each component).[15] The first two of these are general elements that are mostly pre-specified and provided to sites in a standardised manner. The next four are tailored closely to the needs of each site. The final component of this implementation strategy is the role of the quality improvement facilitator which permeates both general and site-specific implementation strategies.

All elements of this implementation strategy are rooted in an assessment of learning needs at each site, the development of rapport with staff, and a visible response to ongoing feedback. They are intended to empower clinical staff to appropriately use PCOMs, including guidance on ensuring the frequency/timing of collection is suitable for the setting in which they are working (see table 3). This may take the form of sharing formal knowledge about PCOMs and how to use them, practical and technical knowledge about IT systems, and/or experiential knowledge about the use of PCOMs in practice. Where PCOM use is affected by team or organisational issues, quality improvement facilitators can empower clinical staff to address these issues and may also play an advocacy role through liaison with senior clinical staff or management. A more in-depth version of the RESOLVE PCOM implementation

For peer review only - http://bmjopen.bmj.com/site/about/guidelines.xhtml

strategy (mapped against the different components included in the TIDIER checklist [21]) can be found in supplementary file 1.

Table 4: An outline of the main components of the RESOLVE PCOM implementation strategy

Implementation component	Characteristics
**General implementation strategies**	
**1. Development of educational resources**	Providing healthcare professionals with resources (e.g., brief instructional videos and pocket guides/outcomes manual) aiming to enhance their knowledge and skills of what outcome measures are, and how to use the right measures at the right time in clinical practice
**2. Workshop and conference events**	Developing and running national and regional workshop events that aim to address common challenges to implementation and facilitate collaborative learning networks between palliative care services
**Site-specific implementation strategies**	
**3. Determining organisational needs**	A baseline assessment - developed through initial interview data and additional liaison with service leads at each site – aimed at understanding the site-specific challenges to implementing PCOMs so that the intervention can be tailored to each site's needs. This includes understanding whether sites want help with better implementing a measure that is already used, or with implementing new measure(s) across or in specific settings
**4. Formal training**	Face-to-face and online educational sessions/presentations with healthcare professionals at sites in order to address local implementation issues/challenges
**5. I.T. support**	Providing informational, practical, and technical support to ensure that each site has the analytic capacity to input outcomes data into, and extract it back out of, electronic systems
**6. Reporting and feedback**	Providing sites with tailored, service-level reports and feedback of their outcomes data to motivate continued use
**Running through general and site-specific implementation strategy**	
**7. Quality improvement facilitator**	Working directly with sites by local site liaison, championing the implementation of PCOMs into practice, identifying and responding to local challenges/needs, keeping PCOMs on the agenda, and acting as a direct link between the research team and sites. An important aspect of this role is to make judicious use of the implementation strategy elements, rather than necessarily deliver the strategy as a whole

Theoretical approach and causal assumptions

As part of their guidance on process evaluations, Moore et al. [15] recommend drawing on theory to propose causal assumptions underpinning how and why The RESOLVE PCOM Implementation Strategy is expected to work. To this end, the components of our

For peer review only - http://bmjopen.bmj.com/site/about/guidelines.xhtml

implementation strategy are theoretically informed by Normalisation Process Theory (NPT).[22, 23] The reason behind selecting NPT is because there is evidence of its effectiveness and value in implementing complex interventions within healthcare settings.[23]

NPT is a type of implementation theory that describes the different types of 'work' that individuals and organisations do in order to 'normalise' complex interventions into clinical practice (i.e., such deep embedment that they become 'invisible'). It does so through proposing four inter-related constructs:

Coherence: The 'sense-making work' that individuals and teams do in order to understand PCOMs.

Cognitive participation: The 'relational work' that occurs in legitimising PCOMs and building everyday clinical practice around using them.

Collective action: The 'operational work' that is performed in order to use PCOMs, including the skills-sets of a workforce and the organisational resources in place that enable the use of PCOMs.

Reflexive monitoring: The 'appraisal work' through which people assess and evaluate the relative value of PCOMs.[22, 24]

We therefore propose that the RESOLVE PCOM implementation strategy will facilitate the implementation of PCOMs into routine practice in four ways: (1) by enhancing professionals' understanding and awareness of how and why to use PCOMs (i.e., improving coherence); (2) by creating an organisational culture where PCOMs are valued and seen as a legitimate way to improve patient care (i.e., enhancing cognitive participation); (3) by ensuring that systems and resources are in place that allows healthcare professionals to easily and meaningfully use PCOMs in everyday practice (i.e., facilitating collective action); and (4) by providing

For peer review only - http://bmjopen.bmj.com/site/about/guidelines.xhtml

meaningful feedback on PCOMs data as to motivate people to continue using them (i.e., enhancing reflexive monitoring).

Patient and public involvement

We support the UK Standards for Public Involvement in research as outlined by the National Institute of Health Research. [25] We have already conducted patient public involvement for strand one of this project (the collection and use of routine outcomes data) in which we consulted with a diverse group of patients about implementation. Furthermore, the RESOLVE project has recruited a lay representative who attends regular project meetings and will be involved in the discussions regarding the interpretation and dissemination of study findings throughout the duration of the project.

Strand one: Mapping change through routinely collected outcomes data

Sampling and recruitment

In recruiting to this study, we will send an invitation letter and participant information sheet to local collaborators at each site. Should sites wish to participate, they will be required to sign and return the study invitation letter. We expect all 11 RESOLVE-affiliated sites to participate in this project.

Data collection

The RESOLVE team have already been working with sites in implementing the palliative Phase of Illness, Integrated Palliative care Outcomes Scales (IPOS), and Australia-modified Karnofsky Performance Status (AKPS) into routine clinical practice. Sites will be required to submit pseudonymised data related to these PCOMs to the RESOLVE team at baseline and follow-up (between 10-12 months later). A more detailed description of the data specification can be found in supplementary file 2.

For peer review only - http://bmjopen.bmj.com/site/about/guidelines.xhtml

Data Analysis

In order to map whether or not the uptake and routine use of PCOMs changes over time, we will use descriptive and analytical statistics to report the levels of missing data at baseline and follow-up (missing complete measures, and missing items).

For each site at baseline, we will assess the missing outcome data at three timepoints: a) start of episode of care, b) first change of Phase of Illness, and c) end of episode of care. To highlight the change in routine use of PCOMs, we will compare the missing outcome data between baseline and follow-up at these 3 timepoints. We will assess missing data on the level of these subscales: physical symptoms (10 items), psychological/emotional symptoms (4 items), and communication/practical issues (3 items). We defined missing outcome data in physical symptoms as 4 or more items missing data out of the 10 items at one point of time. For emotional and communication subscales, missing outcome data was defined as missing at least one item in any of them. We will also compare between the population samples at baseline and follow-up to investigate the comparability of both groups. The variables that we will use to do this will be gender, age, primary diagnosis, and whether a patient lives alone. We will then use t-tests to compare continuous data (e.g., age) with mean and SD, and chi-square to compare categorical data (e.g., gender). All statistical analyses will be conducted at alpha value of 0.05 two-sided level of significance.

Strand two: Online survey data

Conducting survey research will help us to understand whether The RESOLVE PCOM Implementation Strategy has enhanced health care professionals' understandings of, skills in using, and ability to build organisational practices around, PCOMs. The design of the survey strand of this project is informed by Kelley et al.'s [26] guidance on good practice on the conduct and reporting of survey research.

For peer review only - http://bmjopen.bmj.com/site/about/guidelines.xhtml

Sampling and recruitment

We estimate that, when combined, the number of staff across all of the participating services equates to approximately 550 participants. Whilst we aim to distribute this survey to all of these potential participants, we recognise the challenges to online surveys (e.g., participants interests/attitudes to research, communication, structure and length of survey etc.). [27] Therefore, of this number, due to the positive relationships that we have fostered with sites throughout this project, we expect between a 50%-60% response rate. Thus, for the survey part of this research, we estimate between 275-330 participants.

We will use a purposive maximum variation sampling technique [28] as a guide for recruitment, and sample across the following purposive criteria: role, seniority, experience, and settings of care (inpatient, outpatient/day therapy, home-based/community). Through recruiting in this way, we aim to sample an accurate cross-section of the different settings in which PCOMs are used, and the different types of staff members who use them within these settings. This will translate into recruiting staff at different levels/seniority (e.g., those at a medical, senior staff, and junior level) and of differing roles (e.g., doctors, nurses, healthcare assistants, educators, allied healthcare professionals).

To recruit participants, local collaborators at each site will be asked to disseminate an online survey link to healthcare professionals within their organisations. The introductory part of the survey will include the relevant participant information that explains what the purpose of the survey is, why they have been selected, and how data will be used. It will also state that by completing and submitting the survey that they are providing consent to participate in this study. To optimise recruitment and survey completion, we will offer participating sites the incentive of a textbook and/or journal subscription for the organisation for one year.

For peer review only

For peer review only - http://bmjopen.bmj.com/site/about/guidelines.xhtml

Data collection

In keeping with the theoretical framework of this project, we will collect survey data using an adapted version of the NPT survey (The Normalisation MeAsure Development questionnaire (NoMAD). This is a 23-item structured questionnaire that has been developed by the authors of NPT and measures the dynamic processes that underpin the implementation of PCOMs into clinical practice from the perspectives of healthcare professionals directly involved in their use. The NoMAD measure has been found to be an acceptable and psychometrically strong measure which reports good face validity, construct validity, and internal consistency.[29]

Our version of the NoMAD survey consists of three parts which comprise of a combination of closed- and open-ended questions. Part A asks participants to provide demographic information about themselves. Part B asks short questions on what PCOMs participants use and how they use them in everyday practice. Part C is split into four sections, each of which corresponds and asks questions in relation to the different constructs of NPT (coherence, cognitive participation, collective action, reflexive monitoring). This survey can be seen in more detail in supplementary file 3.

We will host our survey online using RedCap software in which participants will be sent a link through which they are able to access and complete the survey. The acceptability of this method, alongside the intelligibility of survey questions, will be piloted on a select number of participants to ensure that they understand the questions being asked and are easily able to complete the online form. Following this, we will make any necessary minor amendments to the NoMAD survey and then administer this to participants at baseline and then at follow-up (10-12 months after baseline collection).

Data analysis

For peer review only - http://bmjopen.bmj.com/site/about/guidelines.xhtml

We will conduct four analyses of survey data:

i. Response rate by capturing the number of participants who completed the NoMAD survey compared to the proportion of those who were approached.
ii. Descriptive statistics to describe the general characteristics of healthcare staff who participated in the survey.
iii. Comparison between respondent answers at baseline and follow-up using statistics tests such as paired t-test and McNemar test (when needed) to indicate any changes in PCOMs usage and understanding. All statistical analyses will be conducted at alpha value of 0.05 two-sided level of significance. We recognise that there are numerous potential limitations/challenges of conducting follow-up surveys 12 months after baseline. These include losing participants to follow-up, withdrawal, staff turnover, changed positions, each of which may lead to decrease the sample size at follow-up time. Thus, we decided if any group lost more than 70% of its participants, we will recruit other health staff from the site (who did not participate at baseline) to complete our follow-up sample. Therefore, we will change the analysis plan and treat both groups two independent samples.
iv. Free-text survey responses will be deductively thematically analysed using NPT constructs (coherence, cognitive participation, collective action, reflexive monitoring) as a framework, whilst also allowing for inductive thematic analysis if responses do not fit within the framework.

Strand three: Focused interview data

For strand three, we will collect focused interview data. The collection of qualitative interview data will be able to provide further insight into research questions two and three by providing rich and insightful data on the processes, mechanisms, and contextual factors that

For peer review only - http://bmjopen.bmj.com/site/about/guidelines.xhtml

underpin the ways in which The RESOLVE PCOM Implementation Strategy does, or does not, work to implement PCOMs into routine practice. [30]

Sampling and recruitment

For each site, depending on organisational size, we will recruit between two-five participants. To do this, we will use purposive sampling [28] (based on roles, disciplines, and experience) as a guide to recruit staff who are directly involved in the implementation of PCOMs across different settings, and able to offer a distinct insight/experience into the issues identified from the survey about each of the NPT constructs. Recruitment will be achieved through working with service leads at each site to approach the relevant participants for interview in this study. Participants will then be approached by their service leads with a participant information sheet. Willing participants will then be required to provide informed consent prior to participation.

Data collection

We will conduct a single focused interview with each participant towards the end of the project (i.e., when we finish our implementation work). These interviews will be used as an opportunity to generate rich insight and details of the processes, mechanisms, and contextual factors that underpin how The RESOLVE PCOM Implementation Strategy does, or does not, work to facilitate the implementation of PCOMs into routine palliative care practice. The interview guide (see supplementary file 4) will consist of a series of questions that relate to the different components of NPT (coherence, cognitive participation, collective action, reflexive monitoring) and will present participants with an opportunity to reflect on how their use of PCOMs has changed over time, and how The RESOLVE PCOM Implementation Strategy has impacted this. In mitigating for the COVID-19 pandemic, these interviews will

For peer review only - http://bmjopen.bmj.com/site/about/guidelines.xhtml

take place online (on whichever platform is most convenient for services) at a time that works for them.

Data analysis

Data will be analysed in NVivo using a thematic framework approach. [31] NPT constructs (coherence, cognitive participation, collective action, and reflexive monitoring) will provide a theoretical framework that will be applied deductively to interview transcripts, whilst also allowing for inductive thematic analysis of responses and outliers that do not fit within the framework. This will be through conducting the following interconnected steps: (1) transcription; (2) familiarisation; (3) coding; (4) developing an analytic framework based on NPT; (5) indexing this framework to transcripts; (6) charting data into a framework matrix (and then performing between- and within-case analyses for each theme); and (7) interpreting the data by using theory.

Outputs: Integration of datasets

Each strand of this project will produce different outputs. In strand one, we will use routine outcomes data to produce a traffic light system in which green represents that PCOMs are used appropriately (e.g., in line with the timings/frequencies of collections outlined in table 3), amber that PCOMs are used but inconsistently (e.g., they are used but not in line with the timings/frequencies of collection in table 3), and red that PCOMs are not being used at all. At a glance, this will provide an objective indicator of whether there has been a change (from baseline to follow-up) in the uptake and use of each PCOM across settings, thus enabling us to determine whether the RESOLVE implementation strategy has been successful in implementing PCOMs into routine palliative care practice.

For strand two, we will produce outputs for NoMAD survey data in the form of spider plot graphs. By calculating how mean changes in each NPT component changes from

For peer review only - http://bmjopen.bmj.com/site/about/guidelines.xhtml

baseline to follow-up, this data will be able to complement our analyses of routine outcomes data by seeing if changes in use of PCOMs status coincides with changes in components of NPT.

For strand three, data from interviews will complement routine outcomes and NoMAD survey data by illuminating the mechanisms as to how and why the use of PCOMs have (or have not) changed over time. It will also be able to provide important information about the contextual/organisational factors that impacted this process.

Figure 1 demonstrates how the integration of outputs produced from the analysis of quantitative and qualitative data may look. It will provide a snapshot that will be able to demonstrate the effectiveness The RESOLVE Implementation Strategy at facilitating the implementation of PCOMs into routine palliative care practice. It will also provide us with explanations as to how, why, and in what contexts our intervention works/needs refining so that it can be tailored for particular organisational contexts and/or settings of care.

[INSERT FIGURE 1 HERE]

For peer review only

For peer review only - http://bmjopen.bmj.com/site/about/guidelines.xhtml

ETHICAL CONSIDERATIONS

Ethical approval for this project was obtained on 16th September 2020 from the East of England - Cambridge Central NHS Research Ethics Committee [ref: 20/EE/0188]. Ethical approval for strands 2 and 3 of this study was obtained on 18th January 2021 from Hull York Medical School ethics committee [21 05].

DISSEMINATION

We will publish the findings of this study in peer reviewed journals and abstracts will be submitted to national and international conferences. We will also disseminate this work to patients, healthcare professionals, academics, and anybody else who is interested in this work through various non-traditional routes. These will include:

- Producing lay briefs of research findings that we will disseminate to patients and service users through our Palliative Daycare Patient Group and the PPIE network at the University of Hull.
- Producing policy briefs of research findings for policymakers and commissioners
- Sharing the results of research and study newsletters on the RESOLVE and Wolfson Palliative Care Research Centre websites and sharing them on social media.
- Delivering educational workshops to clinicians in order to feedback site-specific findings on what does and does not work with regards to implementing PCOMs within their service
- Utilise the Hospice UK's ECHO Network for Outcomes - monthly national interactive webinars to disseminate the results to clinicians, allied health-care professionals, and others who have an interest in palliative care.

For peer review only - http://bmjopen.bmj.com/site/about/guidelines.xhtml

Figure 1 caption: Example of how outputs will be integrated after analysis.

Author contributions: Conceptualisation and design – AB, AK, MP, and FEM. Development of analysis plan – AB, AK, and FEM. Leading the writing process and drafting the original article – AB. Critically reviewed article for important content – AB, MP, MS, AK, KS, JB, MJ and FEM. All authors take responsibility for the protocol and approved the final version of this paper.

Funding statement: This work was supported by Yorkshire Cancer Research, grant number [L412]. Professor Fliss Murtagh is a National Institute for Health Research (NIHR) Senior Investigator. The views expressed in this article are those of the author(s) and not necessarily those of the NIHR, or the UK Department of Health and Social Care.

Competing interest's statement: The author(s) declared no potential conflicts of interest with respect to the research, authorship, and/or publication of this article.

For peer review only - http://bmjopen.bmj.com/site/about/guidelines.xhtml

References

1. Twycross RG. *Introducing palliative care*. Radcliffe Publishing, 2003.
2. Sleeman KE, de Brito M, Etkind S, et al. The escalating global burden of serious health-related suffering: projections to 2060 by world regions, age groups, and health conditions. *The Lancet Global Health* 2019; 7: e883-e892. DOI: 10.1016/S2214-109X(19)30172-X.
3. Donabedian A. *Explorations in quality assessment and monitoring*. Ann Arbor, MI: Health Administration Press, 1980.
4. Dudgeon D. The impact of measuring patient-reported outcome measures on quality of and access to palliative care. *Journal of palliative medicine* 2018; 21: S-76-S-80.
5. Antunes B, Harding R, Higginson IJ, et al. Implementing patient-reported outcome measures in palliative care clinical practice: a systematic review of facilitators and barriers. *Palliative medicine* 2014; 28: 158-175.
6. Bausewein C, Daveson BA, Currow DC, et al. EAPC White Paper on outcome measurement in palliative care: Improving practice, attaining outcomes and delivering quality services–Recommendations from the European Association for Palliative Care (EAPC) Task Force on Outcome Measurement. *Palliative medicine* 2016; 30: 6-22.
7. Etkind SN, Daveson BA, Kwok W, et al. Capture, transfer, and feedback of patient-centered outcomes data in palliative care populations: does it make a difference? A systematic review. *Journal of pain and symptom management* 2015; 49: 611-624.
8. Hill N. Use of quality-of-life scores in care planning in a hospice setting: a comparative study. *International journal of palliative nursing* 2002; 8: 540-547.
9. Currow DC, Allingham S, Yates P, et al. Improving national hospice/palliative care service symptom outcomes systematically through point-of-care data collection, structured feedback and benchmarking. *Supportive Care in Cancer* 2015; 23: 307-315.
10. Greenhalgh J. The applications of PROs in clinical practice: what are they, do they work, and why? *Qual Life Res* 2009; 18: 115-123. 2008/12/24. DOI: 10.1007/s11136-008-9430-6.
11. Dunckley M, Aspinal F, Addington-Hall JM, et al. A research study to identify facilitators and barriers to outcome measure implementation. *International journal of palliative nursing* 2005; 11: 218-225.
12. Pinto C, Bristowe K, Witt J, et al. Perspectives of patients, family caregivers and health professionals on the use of outcome measures in palliative care and lessons for implementation: a multi-method qualitative study. *Annals of palliative medicine* 2018; 7: S137.
13. Bradshaw A, Santarelli M, Mulderrig M, et al. Implementing person-centred outcome measures in palliative care: An exploratory qualitative study using Normalisation Process Theory to understand processes and context. *Palliative Medicine*; 0: 0269216320972049. DOI: 10.1177/0269216320972049.
14. Craig P, Dieppe P, Macintyre S, et al. Developing and evaluating complex interventions: the new Medical Research Council guidance. *BMJ* 2008; 337: a1655. DOI: 10.1136/bmj.a1655.
15. Moore GF, Audrey S, Barker M, et al. Process evaluation of complex interventions: Medical Research Council guidance. *BMJ : British Medical Journal* 2015; 350: h1258. DOI: 10.1136/bmj.h1258.
16. Witt J, Murtagh F, de Wolf-Linder S, et al. Introducing the Outcome Assessment and Complexity Collaborative (OACC) Suite of Measures-A Brief Introduction. *Kings College London* 2014.
17. Masso M, Allingham SF, Banfield M, et al. Palliative care phase: inter-rater reliability and acceptability in a national study. *Palliative medicine* 2015; 29: 22-30.
18. Hearn J and Higginson I. Development and validation of a core outcome measure for palliative care: the palliative care outcome scale. Palliative Care Core Audit Project Advisory Group. *BMJ Quality & Safety* 1999; 8: 219-227.
19. Murtagh FE, Ramsenthaler C, Firth A, et al. A brief, patient- and proxy-reported outcome measure in advanced illness: Validity, reliability and responsiveness of the Integrated Palliative care

For peer review only - http://bmjopen.bmj.com/site/about/guidelines.xhtml

Outcome Scale (IPOS). *Palliative medicine* 2019; 33: 1045-1057. 2019/06/12. DOI: 10.1177/0269216319854264.

20. Abernethy AP, Shelby-James T, Fazekas BS, et al. The Australia-modified Karnofsky Performance Status (AKPS) scale: a revised scale for contemporary palliative care clinical practice [ISRCTN81117481]. *BMC Palliative Care* 2005; 4: 7. DOI: 10.1186/1472-684X-4-7.

21. Hoffmann TC, Glasziou PP, Boutron I, et al. Better reporting of interventions: template for intervention description and replication (TIDieR) checklist and guide. *BMJ : British Medical Journal* 2014; 348: g1687. DOI: 10.1136/bmj.g1687.

22. May CR, Mair F, Finch T, et al. Development of a theory of implementation and integration: Normalization Process Theory. *Implementation Science* 2009; 4: 29.

23. May CR, Cummings A, Girling M, et al. Using Normalization Process Theory in feasibility studies and process evaluations of complex healthcare interventions: a systematic review. *Implementation Science* 2018; 13: 80.

24. Murray E, Treweek S, Pope C, et al. Normalisation process theory: a framework for developing, evaluating and implementing complex interventions. *BMC medicine* 2010; 8: 63.

25. Research NIoH. UK standards for public involvement: Better public involvement for better health and social care research, https://www.invo.org.uk/current-work/standards/ (2021).

26. Kelley K, Clark B, Brown V, et al. Good practice in the conduct and reporting of survey research. *International Journal for Quality in Health Care* 2003; 15: 261-266. DOI: 10.1093/intqhc/mzg031.

27. Saleh A and Bista K. Examining Factors Impacting Online Survey Response Rates in Educational Research: Perceptions of Graduate Students. *Online Submission* 2017; 13: 63-74.

28. Etikan I, Musa SA and Alkassim RS. Comparison of convenience sampling and purposive sampling. *American journal of theoretical and applied statistics* 2016; 5: 1-4.

29. Finch TL, Girling M, May CR, et al. Improving the normalization of complex interventions: part 2 - validation of the NoMAD instrument for assessing implementation work based on normalization process theory (NPT). *BMC Medical Research Methodology* 2018; 18: 135. DOI: 10.1186/s12874-018-0591-x.

30. Sparkes AC and Smith B. *Qualitative research methods in sport, exercise & health. From process to product*. London: Routledge, 2014.

31. Ritchie L, Lewis J, Nicholls CM, et al. *Qualitative research practice: A guide for social science students and researchers* 2ed. Los Angeles, CA: Sage, 2013.

For peer review only - http://bmjopen.bmj.com/site/about/guidelines.xhtml

Figure 1: Example of how outputs will be integrated after analysis.

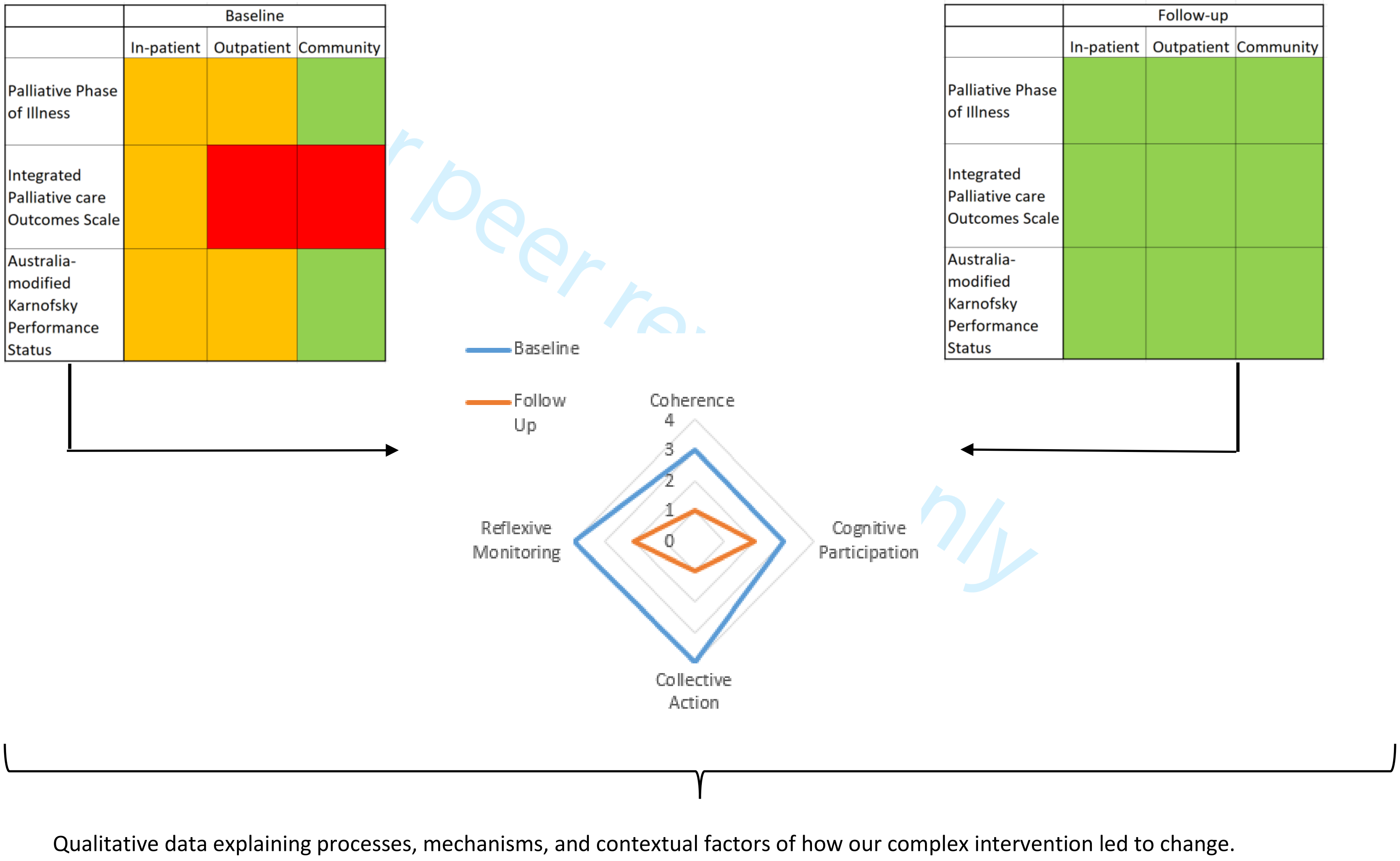

Aspects of implementation<br>TIDIER Checklist Components<br>Component Name	Why	What	Who	How	Where	When/How much	Tailoring	Modifications	How well
**General implementation strategies**									
**Development of educational resources**	To enhance healthcare professionals' knowledge of what outcome measures are, their skills in using them, and how they may build practice around their use.	***Materials and procedures:***<br>Providing healthcare professionals with informational support to facilitate their understanding of what outcome measures are and how/why to use them. This is through the development of teaching and learning materials, including:<br><br>Pocket-guides, outcomes manual, instructional videos, online quizzes	Quality improvement facilitator (Martina), research nurse (Kath), PI (Fliss)	Print-based resources that can be used as an aide to memoir<br><br>Online resources (e.g., quizzes and instructional videos for each measure) available online through the RESOLVE website	Materials are for use during clinical practice and for reflection	Provided to all RESOLVE sites, with additional material being sent upon request	n/a. All sites have access to the same genenral educational resources	To complete retrospectively	To complete retrospectively
**Workshops and conference events**	Workshop and conference events aimed at responding to and discussing the common challenges that participating sites experience with regards to collecting, storing, and using outcome measures	***Materials and procedures:***<br>Providing informational support through teaching and guidance materials on outcome measures. This is through:<br><br>- Collective workshops and conference training events including representatives from all sites across the project	Entire RESOLVE team	Powerpoint presentations, collaborative group-based activities, question and answer sessions, and expert panel discussions.<br><br>Site representatives present at these events are encouraged to disseminate the knowledge and resources gained from attendance with their colleagues at site.	Conference/workshop events	Twice a year.	The content and agendas of workshop and conference events are derived from the common needs shared across sites participating in RESOLVE	To complete retrospectively	To complete retrospectively
**Site-specific implementation strategies**									
**Determining organisational needs**	Aimed at understanding the site-specific challenges to implementing PCOMs so that we can tailor our intervention according to each site's needs	A baseline assessment - developed through initial interview data and additional liaison with service leads at each site	Andy (research fellow) Martina (QIF)	Interviews, email, telephone communications, site visits	All sites	At the start of tailoring our intervention for each site and revisited regularly throughout implementation	Tailoring intervention components to site-specific needs	To complete retrospectively	To complete retrospectively
**Formal training**	Enhance health care professionals' individual and collective understanding of, skills in using, and ability to build organisational practices around, PCOMs	***Materials and procedures:***<br>Providing participating sites with informational and organisational support through teaching and guidance on outcome measures. This is through:<br><br>- Site-based training sessions	Quality improvement facilitator (Martina), research nurse (Kath), PI (Fliss)	Working with sites to understand the challenges that they face to using outcome measures in practice. This information is then used to develop formal training sessions (e.g., presentations, case studies etc.) that help in addressing these issues. This is so that we can ensure that participating sites have the knowledge, understanding, and appropriate organisational resources in place to facilitate the consistent collection and use of outcome measures	At sites	Upon request from site	The nature and content of training sessions are tailored to each site's needs and priorities	To complete retrospectively	To complete retrospectively
**I.T. support**	To ensure local I.T. system readiness. That is, making sure that participating services have the analytic capacity and infrastructure in place so that they are able to input the appropriate data items into an electronic system and extract/ submit these to the RESOLVE team	***Materials and procedures:*** Informational, practical and technical support provided to data teams/analysts to assist them with inputting outcomes data into, and extracting it out of, their electronic system	Fliss (PI) and Assem (Data Analyst)	Practical and technical advice via face-to-face site visits, email and telephone	At sites/electronically	Upon request from site	Site specific support for:<br>- Inputting<br>- Viewing and using<br>- Extraction<br>- Submission of routine outcomes data to RESOLVE team	To complete retrospectively	To complete retrospectively
**Reporting and feedback**	Motivating and reinforcing the use of outcome measures through providing sites with meaningful feedback of their outcomes data. This can then be used to demonstrate how what they do as individuals/teams/organisations impacts patient care and outcomes	***Materials and procedures:***<br>Producing site based reports and graphs on outcomes data through our Registry protocol and feeding this back to each site.	Fliss (PI) and Assem (Data Analyst)	Electronic feedback via email	Electronic	Twice yearly	Site-specific support for:<br>- Producing outcomes reports<br>- Using outcomes data to inform practice at patinet, team, and service levels	To complete retrospectively	To complete retrospectively
**Running through general and site-specific implementation strategies**									
**Quality improvement facilitator (QIF)**	To champion the implementation of PCOMs at sites	Liase and work work sites locally in driving the implementation of PCOMs into practice, identifying and responding to local challenges/needs, keeping PCOMs on the agenda, and acting as a direct link between the research team and sites.	QIF (Martina)	Face-to-face support, email and telephone correspondence	At site	Throughout the RESOLVE implementation strategy	All support from the WIF will be tailored to local site needs and challenges	To complete retrospectively	To complete retrospectively

For peer review only

Version 1.0 08/07/2019

Data item specification

Note: Whilst we plan on collecting data items on dates (e.g., date of referral, first contact data, date ready for care etc.), these will be pseudonymised in Excel by participating sites prior to being submitted to the Registry through the formula below.

Before pseudonymising dates, please make sure that there are no cases from before the date requested. For example, if data is requested from June onwards, disregard any cases with a starting episode date before June.

1. Excel handles dates numerically in the form of 'serial numbers', with each date being represented by a specific serial number. For example, the serial number for the date 24/10/2019 is '42667'. In pseudonymising dates, therefore, each site will use Excel to convert each date (dd/mm/yy) into its serial number through formatting cells into ‘general’ instead of ‘date’.

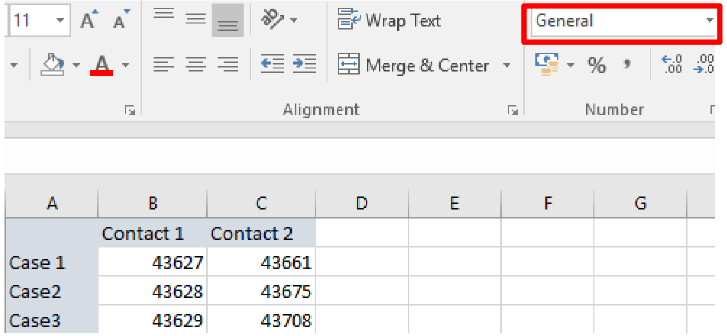

2. Hospices will then select and consistently use a random number between 1-500 (that the research team will not be made aware of) and add this to the serial number before submitting it to the registry.

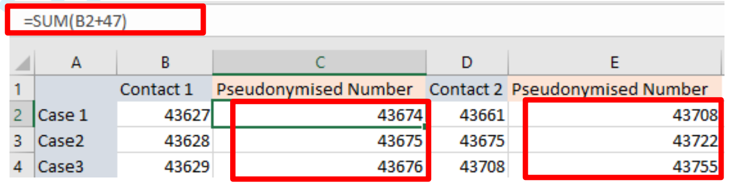

3. This will allow dates to be pseudonymised without the research team being able to trace back to the original date. The numbers that are produced can then be used to determine temporal factors of care that we are interested in(e.g., length of stay, time from referral to first contact) but without receiving dates which may identify participants.

For peer review only - http://bmjopen.bmj.com/site/about/guidelines.xhtml

Version 1.0 08/07/2019

Level 1: Patient Details – reported at patient level

No.	Variable Name	Definition	Format/Document	Use
1	Site ID	Unique identifier for each provider	A two letter and two digit code issued by the RESOLVE team (e.g., AA00)	To identify what palliative care provider is submitting data to the registry
2	Pseudonymised Unique Patient ID	A unique and pseudonymised patient identifier within the service's IT system that is used to identify the patient that is being cared by service providers.	This will be created by applying a macro to the NHS number, which provides a unique code which cannot be use to reverse to NHS number	To ensure the positive identification of a patient and that each individual's health records are associated with that individual and no other, while also enabling identification of repeat episodes of care for any one individual.
3	Age	The age of a patient at the start of their first episode of care	Age (in years old)	To determine the age of individual patients for conducting demographic analyses.
4	Gender	The gender of a patient	One of the following:<br>• Male<br>• Female<br>• Other<br>• Unknown	To determine gender of individual patients in a standardized format so that data can be used for related to service utilization, service needs, and epidemiological studies
5	Ethnicity	The ethnicity of the patient	One of the following:<br>• Mixed/multiple ethic groups<br>• Asian/Asian British<br>• Black/African/Caribbean/Black British<br>• Other ethnic group<br>• White	To determine ethnicity of individual patients in a standardized format so that data can be used for related to service utilization, service needs, and epidemiological studies
6	Primary Diagnosis	The primary life-limiting illness responsible for the patient receiving palliative care	Options showed in appendix 1 (ICD10 classification)	To determine a patients primary diagnosis.
7a	Secondary Diagnosis 1	Any secondary conditions / co-morbidities the patient has.	Options showed in appendix 1 (ICD10 classification)	To determine a patients secondary diagnosis.
7b	Secondary Diagnosis 2			
7c	Secondary Diagnosis 3			
8	Lives alone?	Whether or not the patient lives alone.	One of the following options:<br>• Yes	To understand a patients living arrangements for analysis (e.g., impact of living arrangements of cost of care and other outcomes)

For peer review only

Version 1.0 08/07/2019

No.	Variable Name	Definition	Format/Document	Use
			• No<br>• Unknown	
9	Usual Place of Residence	The patient's current main or permanent place of residence	One of the following:<br>• Patient's own home<br>• Other Private Residence (e.g., Relative's/Carers home)<br>• Care Home (residential/nursing facility)<br>• Hospice (inpatient specialist palliative care)<br>• Hospital<br>• Homeless<br>• Traveler<br>• Other	To understand place of care
10	Marital Status	A patient's marital status.	One of the following options:<br><br>• Single<br>• Married/Civil Partner/Cohabiting<br>• Divorced/Person whose Civil Partnership has been dissolved<br>• Widowed/Surviving Civil Partner<br>• Separated<br>• Not disclosed<br>• Unknown	To understand a patients marital status for analysis

For peer review only

Level 2: Episode Details – reported at episode level

No.	Variable Name	Definition	Format/Document	Use
11	Palliative episode number	The number episode that a patient is in	The corresponding number with a patient's episode (e.g., 1 for their first episode of care, 2 for their second, 3 for their third etc.)	To identify the number episodes a patient has experienced.
12	Referral Date	The date that a service receives a referral to provide palliative care for a patient for this episode	Date of referral (DD/MM/YYYY)	This will serve as a **key benchmarking variable**; the measurement of the time between referral and other variables (e.g., first contact date, date ready for care, episode start date). ***This date will be pseudonymised using the formula outlined at the start of this document.**
13	Primary Reason for referral	The primary reason for referral to specialist palliative care, as identified by person who refers patient.	One of the following options:<br>• Pain<br>• Other symptom control<br>• Emotional/psychological/spiritual support (patient)<br>• Emotional/psychological/spiritual support (family)<br>• Social/financial support (patient)<br>• Social/financial support (family)<br>• Rehabilitation<br>• Discharge planning<br>• Care in last days of life<br>• Respite<br>• Other	For analyses regarding why patients are referred to specialist palliative care services
14	Referral Source	The facility/organization which the patient was referred from for the start of a particular episode.	One of the following:<br>• Hospital – NHS<br>• Hospital – Outpatient<br>• Hospice – Inpatient<br>• Hospice – Day services<br>• Hospice – Outpatient<br>• Community – GP<br>• Community – Hospice at home<br>• Community – Out of hours services	To assist in understanding referral patterns and service planning.

For peer review only - http://bmjopen.bmj.com/site/about/guidelines.xhtml

Version 1.0 08/07/2019

No.	Variable Name	Definition	Format/Document	Use
			• Community – District/Community matron<br>• Self-referral/family-referral<br>• Internal referral within organisation<br>• Other (please state)	
15	First Contact Date	The date the clinical team makes contact with patient to assess needs after referral	Date of first clinical contact (DD/MM/YYYY)	This is a **key benchmarking variable** as it can be used to measure response time. ***This date will be pseudonymised using the formula outlined at the start of this document.**
16	Date Ready for Care	The date the patient is ready and available to receive palliative care. This may be determined through referral or first contact with patient	Date identified that patient is ready to receive palliative care (DD/MM/YYYY)	**Key benchmarking variable** as it is used to identify and account for early referrals (i.e., when a referral is received by a patient is not ready for care). ***This date will be pseudonymised using the formula outlined at the start of this document.**
17	Episode Start Date	The date of the first face-to-face palliative care assessment, using the set of core outcomes listed above. This is when clinical care starts	The date when clinical care starts (DD/MM/YYYY)	This date (along with episode end date) will be used to determine the length of stay for each episode of care. ***This date will be pseudonymised using the formula outlined at the start of this document.**
18	Care Setting	The setting/location where a patient is overnight.	One of the following:<br>• Hospital – Inpatient<br>• Hospital – Outpatient<br>• Hospice – Inpatient<br>• Community – Outpatient<br>• Community - Home visiting<br>• Community – Day care or day service<br>• Community – Nursing/residential home<br>• Community – Patient's/Carer's own home<br>• Community – Community hospital<br>• Other (please state)	To determine where an episode of care takes place
19	Team delivering care	The team delivering care within setting	Provided by the reporting organisation	Each organisation has different 'labels' for their teams (such as different clinics, therapy teams etc), yet may require sub-analysis by team to support effective quality improvement – we will therefore allow each reporting organisation to self-specify this variable
20	Episode end date	The date when a patient moves from one setting of care to another, dies, or	Date of episode end (DD/MM/YYYY)	To use with episode start date to determine the length of care for each episode. ***This date will be pseudonymised using the formula outlined at the**

For peer review only

Version 1.0 08/07/2019

No.	Variable Name	Definition	Format/Document	Use
		is no longer receiving palliative care		**start of this document.**
21	Episode result	The reason that an episode of palliative care ended	One of the following:<br>• Discharged to own home (or relatives/carers home)<br>• Discharged to nursing/residential home<br>• Discharged to hospital<br>• Discharged to NHS hospice<br>• Discharged to non-NHS hospice<br>• Died<br>• Unknown	Describes how the episode ended, determines number of deaths, and discharge locations.
22	Preferred place of death (if relevant)		One of the following:<br>• Nursing/residential home<br>• Home<br>• Hospice<br>• Hospital<br>• Other	
23	Whether patient is now deceased		• Yes<br>• No<br>• Unknown	Reporting may occur some time after episodes of care are completed. Knowing if the patient subsequently died will enable reporting of how early in the trajectory of illness patients are being seen.
24	Date of death		Date of death (DD/MM/YYYY)	This date will be used to determine at what point in the illness trajectory, the episode of care was delivered. ***This date will be pseudonymised using the formula outlined at the start of this document.**
25	Place of death (if relevant)		One of the following:<br>• Nursing/residential home<br>• Home<br>• Hospice<br>• Hospital<br>• Other	

For peer review only

Version 1.0 08/07/2019

Level 3: Phase Details – reported at contact level

No.	Variable Name	Definition	Format/Document	Use
26	Palliative Phase Number	The number phase that a patient has within an episode.	The corresponding number with a patient's phase (e.g., 1 for their first phase of illness, 2 for their second, 3 for their third etc.)	To identify the number phases a patient has experienced within an episode and to work out number of patient contacts.
27	Palliative Phase of Illness	Stage of a patient's illness, assessed at the start of the phase as assessed by Phase of Illness questionnaire (see appendix X)	One of the following:<br>• Stable<br>• Unstable<br>• Deteriorating<br>• Dying<br>• Deceased<br>• Bereavement/post death support<br>• Unknown (see appendix X for phase definitions)	To identify the stage of a patient's illness
28	Phase Start Date	The date each new phase of illness begins	Phase start date (DD/MM/YYYY)	When used with phase end date, can tell us the length of a phase of illness. ***This date will be pseudonymised using the formula outlined at the start of this document.**
29	Phase End Date	The date each phase of illness ends	Phase end date (DD/MM/YYYY)	When used with phase start date, can tell us the length of a phase of illness. ***This date will be pseudonymised using the formula outlined at the start of this document.**
30	Is this the End of a Phase?	Whether the patient has reached the end of a phase of illness.	One of the following:<br>• Yes<br>• No	To clarify the end of a phase of illness
31	Has the Phase Changed?	To highlight whether a patient has undergone a change in their phase of illness.	One of the following:<br>• Start Phase<br>• Continuing Phase<br>• End Phase	To cross check with phase of illness assessments
32	Contact Date	The date a health-care professional has clinical contact with patient.	DD/MM/YYYY.	To align contact dates – and clinical data collected at these - with changes in episodes and phases. ***This date will be pseudonymised using the formula outlined at the start of this document.**
33	Australia-Modified Karnofsky Performance	An assessment a patient's functional status at the beginning of a phase of illness	Functional status, as measured by AKPS ranging from 0-100% in 10% increments, (where 100% is fully functioning and 10% is unarousable). See	**Key benchmarking variable**. An analysis of functional status at phase start

For peer review only

Version 1.0 08/07/2019

No.	Variable Name	Definition	Format/Document	Use
	Scale (AKPS) Score		appendix X for scale	
34	Integrated Patient Outcomes Scale (IPOS) at	An assessment of a patient's symptoms and other concerns at phase start (see items between numbers X-X)	Inputting data from each item of IPOS (see appendix X)	**A key benchmarking variable**. A measure of a patient's perceptions of their symptoms at the start of a phase of illness
35	Barthel Activities of Daily Living	To assess patient's activities of daily living at phase start	Inputting data from each item of BARTHEL (see appendix X)	
36	Views on Care (VOC)	To assess a patient's ratings of their quality of life, overall well-being, and their view of the impact of the service on their problem(s) at the start of a phase of illness	Inputting data from each item of VOC (see appendix X)	To measure a patients views on care

For peer review only

Version 1.0 08/07/2019

Appendix 1: ICD10 Classifications

No.	RESOLVE Diagnoses Groups
1	Cancer - Cancer of bone, skin, mesothelial and soft tissue, thyroid or endocrine [C40-C49 & C73-C75]
2	Cancer - Cancer of breast [C50]
3	Cancer - Cancer of independent multiple sites [C97]
4	Cancer - Cancer of liver, intrahepatic bile ducts, gallbladder [C22-C24]
5	Cancer - Cancer of respiratory & intrathoracic organs, including lung (C30-C39)
6	Cancer - Cancer of the digestive organs, including colon, rectum, stomach, excluding liver, GB, pancreas [C15-C21 & C26]
7	Cancer - Cancer of the eye, brain and other CNS [C69-C72]
8	Cancer - Cancer of the female genital organs [C51-58]
9	Cancer - Cancer of the lip, oral cavity & pharynx [C00-C14]
10	Cancer - Cancer of the male genital organs, including prostate [C60-C63]
11	Cancer - Cancer of the pancreas [C25]
12	Cancer - Cancer of the urinary tract [C64-C68]
13	Cancer - Cancer of unknown primary or other unspecified [C76-C80]
14	Cancer - Lymphoid & haematopoietic cancer [C81-C96]
15	Multiple non-cancer conditions - addition to help with multi-morbidity
16	Non-cancer - All other non-cancer diagnoses
17	Non-cancer - Chronic renal failure [N18]
18	Non-cancer - Chronic respiratory disease [J40-J70]
19	Non-cancer - Dementia including Alzheimer's disease [G30 & F00-F03]
20	Non-cancer - Diabetes mellitus [E10-E14]
21	Non-cancer - Heart failure [I50]
22	Non-cancer - HIV disease/AIDS [B20-B24]
23	Non-cancer - Liver failure, chronic liver disease, other non-malignant liver disease [K70-K77]
24	Non-cancer - Motor neurone disease [G12]
25	Non-cancer - Neurological conditions (excluding MND and Alzheimer's) [G00-G99

For peer review only

Version 1.0 08/07/2019

No.	RESOLVE Diagnoses Groups
	excluding G12 & G30]
26	Non-cancer - Other heart & circulatory conditions excluding heart failure and stroke [I00-I99 excluding I50, I61, I63]
27	Non-cancer - Stroke, infarction or haemorrhagic [I61 & I63]
28	Unknown

For peer review only

Version 1 (15/12/2020) **IMPLEMENTING PERSON-CENTRED OUTCOMES MEASURES INTO PALLIATIVE CARE**

Information about this survey

What is this survey about?

This survey is part of a study called 'RESOLVE'. RESOLVE is a research programme aimed at facilitating the implementation of Person-Centred Outcome Measures into routine palliative care. This is so that, as a palliative care community, we ensure regular assessment and monitoring of symptoms and other concerns of those receiving palliative care. As part of our project we have developed an intervention strategy, and this survey is one way in which we are trying to understand if it is effective at facilitating the integration of outcome measures into everyday practice. More information on the RESOLVE project can be found through our website: https://www.hyms.ac.uk/research/research-centres-and-groups/wolfson/resolve.

Why have I been chosen?

You have been selected to complete this survey as you are a staff member at a RESOLVE-affiliated site that has received the RESOLVE implementation strategy. Sites with the best completion rate will receive a voucher for a textbook or one-year journal subscription.

Do you consent to taking part in this survey?

☐ Yes ☐ No

What about my data?

By completing and submitting this survey you are providing consent for your responses to be used as part of the RESOLVE research project. All data is anonymised and confidential and will be stored in accordance with General Data Protection Regulation (GDPR). If you have any questions, please contact the RESOLVE team (resolve@hyms.ac.uk).

Survey Instructions

This survey is split into 3 parts and should take no longer than 15 minutes to complete. Part A asks some brief questions about yourself and your role. Part B includes questions about what outcome measures you use and how you use them to inform your everyday practice. Part C asks more detailed questions about PCOMs.

For peer review only - http://bmjopen.bmj.com/site/about/guidelines.xhtml

Please take the time to decide which answer **best suits your experience for each statement and tick the appropriate box**

Part A: About yourself

➢ What is your professional role?

☐Nurse ☐Doctor ☐Allied health professional ☐Healthcare assistant ☐Chief exec/manager ☐ I.T. or data ☐Other (dropdown box)

➢ What is the name of your organisation?

☐ Dove House Hospice ☐ Marie Curie Bradford Hospice ☐ Wheatfields Hospice ☐ St Gemma's Hospice ☐ St Andrew's Hospice
☐ Grimsby Care Plus Group ☐ Kirkwood Hospice ☐ St Luke's Hospice ☐ St Catherine's Hospice
☐ City Health Care Partnership Hull ☐ Bradford District Care Trust ☐ York Teaching Hospital NHS Foundation Trust

➢ What is your age?

☐ 18-24 ☐ 25-34 ☐ 35-44 ☐ 45-54 ☐ 55+ ☐ Prefer not to say

➢ What is your gender?

☐ Male ☐ Female ☐ Other ☐ Prefer not to say

➢ By which ethnicity do you identify?

☐ White ☐ Black/African/Caribbean/Black British ☐ Asian/Asian British ☐ Mixed/multiple ethic groups ☐ Other
☐ Prefer not to say

➢ What setting do you work in (tick multiple if you work across settings)?

☐ In-patient hospice ☐ Outpatient/day therapy ☐ Community ☐ Hospital

➢ How long have you worked in palliative care?

☐ Less than a year ☐ 1-5 years ☐ 6-10 years ☐ 11-15 years ☐ 15+ years

➢ From the statements below, please choose an option that best describes ***your main role*** in relation to outcome measures:

☐ I am involved in managing or overseeing the implementation and use of outcome measures
☐ I am involved in using outcome measures as part of my everyday practice
☐ I use outcome measures occasionally as part of my everyday practice
☐ I am not using outcome measures as part of my everyday practice

For peer review only
For peer review only - http://bmjopen.bmj.com/site/about/guidelines.xhtml

Part B: What outcome measures do you use?

Outcome measures are short questionnaires that provide healthcare professionals with information on a person's own perception of their health status and well-being. They include palliative Phase of Illness, Integrated Palliative care Outcome Scale (IPOS), and Australia-modified Karnofsky Performance Scale (AKPS). This survey relates to your use of these outcome measures.

➢ Do you use the following outcome measures?

	At 1st assessment	At follow-up
palliative Phase of Illness	☐ Yes ☐ No	☐ Daily ☐ Weekly ☐ At clinical assessment ☐ Other (open free text response box)

	At 1st assessment	At follow-up
Integrated Palliative care Outcome Scale (IPOS)	☐ Yes ☐ No	☐ Yes ☐ No
Australia-modified Karnofsky Performance Scale (AKPS)	☐ Yes ☐ No	☐ Yes ☐ No

➢ At what follow up interval do you collect IPOS and AKPS data?

☐ At change of palliative Phase of Illness ☐ End of episode of care ☐ Other (open free text response box)

➢ Do you or your team use these measures to shape the care of individual patients, including in multi-disciplinary team meetings?

➢ Do you or your team use these measures to shape the services that your team or organisation delivers?

For peer review only

Part C: Detailed questions about outcome measures

These questions are designed to help us find out more about whether people and teams understand what outcome measures are and how they may be used during practice. For each statement please select an answer that best suits your experience.

**Section C1**	Strongly agree	Agree	Neither agree nor disagree	Disagree	Strongly disagree	Not relevant to my role	Don't know
**I can see how outcome measures differs from usual ways of working**	☐	☐	☐	☐	☐	☐	☐
**Palliative care staff in this organisation have a shared understanding of the purpose of outcome measures**	☐	☐	☐	☐	☐	☐	☐
**I understand how using outcome measures affects the nature of my own work**	☐	☐	☐	☐	☐	☐	☐
**I can see the potential value of outcome measures**	☐	☐	☐	☐	☐	☐	☐

- Please feel free to expand on your responses below

For peer review only - http://bmjopen.bmj.com/site/about/guidelines.xhtml

These questions are designed to help us find out more about how everyday practice is built around using outcome measures and how you think they relate to your role. For each statement please select an answer that best suits your experience.

**Section C2**	Strongly agree	Agree	Neither agree nor disagree	Disagree	Strongly disagree	Not relevant to my role	Don't know
**There are key people who drive the use of outcome measures and get others involved**	☐	☐	☐	☐	☐	☐	☐
**I believe that using outcome measures is a legitimate part of my role**	☐	☐	☐	☐	☐	☐	☐
**I am open to working with colleagues in new ways to use outcome measures**	☐	☐	☐	☐	☐	☐	☐
**I will continue to support the use of outcome measures**	☐	☐	☐	☐	☐	☐	☐

- Please feel free to expand on your responses below

For peer review only

These questions are designed to help us better understand the ways in which you experience using outcome measures as part of your role. For each statement please select an answer that best suits your experience.

**Section C3**	Strongly agree	Agree	Neither agree nor disagree	Disagree	Strongly disagree	Not relevant to my role	Don't know
**I can easily integrate outcome measures into existing work**	☐	☐	☐	☐	☐	☐	☐
**Outcome measures disrupt working relationships**	☐	☐	☐	☐	☐	☐	☐
**I have confidence in other people's ability to use outcome measures**	☐	☐	☐	☐	☐	☐	☐
**Work is assigned to those with skills appropriate to using outcome measures**	☐	☐	☐	☐	☐	☐	☐
**Sufficient training is provided to enable palliative care staff to implement and use outcome measures**	☐	☐	☐	☐	☐	☐	☐
**Sufficient resources are available to support outcome measures use**	☐	☐	☐	☐	☐	☐	☐
**Management adequately supports the use of outcome measures**	☐	☐	☐	☐	☐	☐	☐

- Please feel free to expand on your responses below

For peer review only

These questions are designed to help us understand the ways in you appraise the value of outcome measures. For each statement please select an answer that best suits your experience.

**Section C4**	Strongly agree	Agree	Neither agree nor disagree	Disagree	Strongly disagree	Not relevant to my role	Don't know
**I am aware of reports about the effects of outcome measures on patient care**	☐	☐	☐	☐	☐	☐	☐
**The palliative care staff agree that using outcome measures is worthwhile**	☐	☐	☐	☐	☐	☐	☐
**I value the effects that outcome measures have had on my work**	☐	☐	☐	☐	☐	☐	☐
**Feedback about outcome measure data can be used to improve how they are used in future**	☐	☐	☐	☐	☐	☐	☐
**I can modify how I work with outcome measures**	☐	☐	☐	☐	☐	☐	☐

- Please feel free to expand on your responses below

For peer review only

- Are you happy to be contacted to complete this survey again (in around 8-12 months' time)? This is so that we are able to explore the impact of the RESOLVE implementation strategy over time.
  - ☐ Yes (if selected then include dropdown box that asks for email address so that we are able to contact, alongside statement that we will send them a certificate of participation for this survey)
  - ☐ No (if selected then include dropdown box that asks if respondent wants to receive a certification of participation. If they do, prompt for email address)

Thank you very much for completing this survey.

What happens next?

This data will be stored confidentially and analysed as part of the RESOLVE project to understand the ways in which the RESOLVE implementation strategy has (or has not) worked to help implement outcome measures into routine clinical practice. This data will be used as part of reports, publications, and conference presentation. All information included within these outputs will be anonymised and it will not be possible for others to identify you. If you have any questions, please contact the RESOLVE team (resolve@hyms.ac.uk).

For peer review only

Version 1 (15/12/2020)

Introduction to the Interview

Thank you very much for volunteering to be interviewed for the RESOLVE project. The aim of this project is to implement Person-Centred Outcome Measures (PCOMs) into routine palliative care across Yorkshire.

As part of RESOLVE, we have developed an implementation strategy to facilitate the implementation of PCOMs into practice. This strategy was developed based on what health professionals' thought were important challenges that needed to be addressed in order to integrate PCOMs into routine practice. Our strategy includes:

- Educational resources
- Workshop and conference events
- Determining organisational and team needs
- Formal and informal training
- IT support
- Reporting and feedback
- Quality improvement facilitator

The purpose of this discussion is to understand your thoughts and feelings on the extent to which you think this implementation strategy has been effective at enhancing the uptake and routine use of PCOMs in everyday practice. It should take around 30 minutes.

Do you have any questions before we start?

The first few questions are around what measures you currently use and how you use them.

- ➢ Are you able to tell me what palliative care outcome measures you use in your everyday practice? (probe for use of the three core measures: palliative Phase of Illness, AKPS, and IPOS)
- ➢ When were these measures introduced? (probe for when each of the three core was introduced)
- ➢ How do you use these measures in practice? Probe for:
    - Who collects these measures?
    - How often they are collected/timings?

- Which version is used (largely version of IPOS, but also version of Barthel if mentioned)?

- Has your use of outcome measures changed over the last 1-2 years?
  - Collecting?
  - Scoring?
  - Reporting?
  - General understanding of each measure (i.e., what they are, how to use them, their value)?
- Do you think that the way in which you currently use outcome measures is better or worse than previously?
  - What has changed or remained the same?
  - Why?

The next few questions focus on the ways in which you may have used different components of the RESOLVE implementation strategy and how, if at all, you feel as though it may have impacted how you use outcome measures.

- Have you, or your team/service, used any of the RESOLVE implementation strategies
  - Educational resources
  - Workshop and conference events
  - Determining organisational and team needs
  - Formal and informal training
  - IT support
  - Reporting and feedback
  - Quality Improvement Facilitator, or other RESOLVE team members

For peer review only

Version 1 (15/12/2020)

- Can you comment on the extent to which you feel the RESOLVE implementation has been put into practice within your service?
    - Which elements?
    - How much?

Coherence

- What do outcome measures mean to you in relation to your role?
    - Have these views changed over time?
    - Why?
- Do you feel as though the RESOLVE implementation strategy has changed your own (and other colleagues') understandings of what outcome measures are and how to use them?
    - On scoring consistently and confidently?
    - On what they mean (especially Phase of Illness)?
    - Frequency of collection (and selecting the relevant versions)?
- How and why do you think these things?

Cognitive Participation

- Are you able to reflect on the ways in which the RESOLVE implementation strategy may have contributed to building clinical/team practice around outcome measures?
    - Has it influenced communicating with outcome measures in relation to individual patients (i.e., ward rounds, MDT's, handovers etc.)?
    - How?
    - Has it shaped the services that your team or organisation delivers?
    - How?

For peer review only

Version 1 (15/12/2020)

Collective Action

- Thinking back over the last year, has the way in which you use outcome measures (both individually and as part of your team) changed as a result of the RESOLVE implementation strategy?
  - How?
  - What is different now?
  - What parts of the RESOLVE implementation strategy do you feel are responsible for these changes?
- Has the implementation strategy helped to develop your own, and your teams, skills, and confidence in using outcome measure to inform care at a patient/team/service level?
  - What has helped with this?
- Were there any organisational barriers to using outcome measures (individually and as part of your team) that the RESOLVE implementation strategy has helped to address?
  - What were these?
  - How/why did the implementation strategy help?
- Has your confidence to confidently/efficiently input, share, and extract outcomes data within your electronic systems changed?
  - If any, what part of the RESOLVE implementation strategy has been key to this?

Reflexive Monitoring

- Are you able to reflect on the ways in which the RESOLVE implementation strategy has changed your views on how you value outcome measures in everyday practice?

For peer review only

Version 1 (15/12/2020)

  - How?
  - Why?

- How do you share feedback on outcome measures?
  - Do you feel as though the RESOLVE implementation strategy has had an impact on for you/your team/the organisation to do this?
  - How/Why?
- Are there any aspects of the RESOVLE implementation strategy that you think have contributed/will contribute to sustained use of outcome measures?
  - What are these?
  - Why?
  - How will use be sustained once the RESOLVE project is over?

Thank you for taking part in this interview. Before we finish, do you have any other questions?

For peer review only

BMJ Open

Implementing Person-Centred Outcome Measures (PCOMs) into routine palliative care: Protocol for a mixed-methods process evaluation of the RESOLVE PCOM Implementation Strategy

Journal:	*BMJ Open*
Manuscript ID	bmjopen-2021-051904.R2
Article Type:	Protocol
Date Submitted by the Author:	18-Aug-2021
Complete List of Authors:	Bradshaw, Andy; University of Hull<br>Santarelli, Martina; University of Hull<br>Khamis, Assem M; Hull York Medical School<br>Sartain, Kathryn; University of Hull; York and Scarborough Teaching Hospitals NHS Foundation Trust<br>Johnson, Miriam; The University of Hull, Hull York Medical School<br>Boland, Jason; Hull York Medical School<br>Pearson, Mark; Hull York Medical School, Wolfson Palliative Care Research Centre<br>Murtagh, Fliss; Wolfson Palliative Care Research Centre, Hull York Medical School, University of Hull, UK
**Primary Subject Heading**:	Palliative care
Secondary Subject Heading:	Evidence based practice, Patient-centred medicine, Qualitative research, Research methods
Keywords:	PALLIATIVE CARE, Protocols & guidelines < HEALTH SERVICES ADMINISTRATION & MANAGEMENT, Adult palliative care < PALLIATIVE CARE

SCHOLARONE™
Manuscripts

I, the Submitting Author has the right to grant and does grant on behalf of all authors of the Work (as defined in the below author licence), an exclusive licence and/or a non-exclusive licence for contributions from authors who are: i) UK Crown employees; ii) where BMJ has agreed a CC-BY licence shall apply, and/or iii) in accordance with the terms applicable for US Federal Government officers or employees acting as part of their official duties; on a worldwide, perpetual, irrevocable, royalty-free basis to BMJ Publishing Group Ltd ("BMJ") its licensees and where the relevant Journal is co-owned by BMJ to the co-owners of the Journal, to publish the Work in this journal and any other BMJ products and to exploit all rights, as set out in our licence.

The Submitting Author accepts and understands that any supply made under these terms is made by BMJ to the Submitting Author unless you are acting as an employee on behalf of your employer or a postgraduate student of an affiliated institution which is paying any applicable article publishing charge ("APC") for Open Access articles. Where the Submitting Author wishes to make the Work available on an Open Access basis (and intends to pay the relevant APC), the terms of reuse of such Open Access shall be governed by a Creative Commons licence – details of these licences and which Creative Commons licence will apply to this Work are set out in our licence referred to above.

Other than as permitted in any relevant BMJ Author's Self Archiving Policies, I confirm this Work has not been accepted for publication elsewhere, is not being considered for publication elsewhere and does not duplicate material already published. I confirm all authors consent to publication of this Work and authorise the granting of this licence.

For peer review only

Title: Implementing Person-Centred Outcome Measures (PCOMs) into routine palliative care: Protocol for a mixed-methods process evaluation of the RESOLVE PCOM Implementation Strategy

Bradshaw, A [1]., Santarelli, M [1]., Khamis, A [1]., Sartain, K [1,2]., Johnson, M. J [1]., Boland, J. W [1]., Pearson, M [1]., Murtagh, F.E.M [1].*

[1] *Wolfson Palliative Care Research Centre, Hull York Medical School, University of Hull, Hull, UK*

[2] *York and Scarborough Teaching Hospitals NHS Foundation Trust, York, UK*

*Corresponding author: Andy Bradshaw, Allam Medical Building, University of Hull, Hull, HU6 7RX. Email: andrew.bradshaw@hyms.ac.uk

Word count (excluding title page, abstract, references, figures and tables): 4,052

For peer review only - http://bmjopen.bmj.com/site/about/guidelines.xhtml

Abstract

Introduction: Person-centred outcome measures improve quality of care and patient outcomes but are used inconsistently in palliative care practice. To address this implementation gap, we developed the 'RESOLVE Implementation Strategy'. This protocol describes a process evaluation to explore mechanisms through which this strategy does, or does not, support the implementation of outcome measures in routine palliative care practice.

Methods and analysis: Multistrand, mixed methods process evaluation. Strand one will collect routine outcomes data (palliative Phase of Illness, Integrated Palliative care Outcomes Scale, Australia-modified Karnofsky Performance Status) to map the changes in use of outcome measures over 12 months (July 2021-July 2022). Strand two will collect survey data over the same 12-month period to explore how professionals' understandings of, skills in using, and ability to build organisational practices around, outcome measures change over time. Strand three will collect interview data to understand the mechanisms underpinning/affecting our implementation strategy. Thematic Framework analysis and descriptive statistics will be used to analyse qualitative and quantitative data, respectively.

Ethics and dissemination: For strand one, ethical approval has been obtained (Cambridge REC, REF: 20/EE/0188). For strands two and three ethical approval has been obtained from Hull York Medical School ethics committee [21 05]. Tailored feedback of study findings will be provided to participating sites. Abstracts and papers will be submitted to national/international conferences and peer-reviewed journals. Lay and policy briefings and newsletters will be shared through PPI and project networks, plus via the project website.

Keywords: Implementation science; Outcome measures; Palliative care; Process evaluation

For peer review only

Strengths and limitations of this study

- This is the first process evaluation of an implementation strategy designed to facilitate the integration of Person-Centred Outcome Measures into routine palliative care practice.
- This protocol follows the Medical Research Council's guidance on process evaluations by adopting a systematic and theoretically informed approach to study design and conduct.
- We will adopt a multi-strand, mixed methods approach in which we will integrate routine outcomes, survey, and focused interview data to demonstrate the mechanisms through which our intervention strategy may or may not work across different settings of palliative care.
- Since we only focus on implementing a core set of outcome measures, the applicability of findings may be limited to other outcome measures, many of which have measure-specific challenges to implementation.

For peer review only - http://bmjopen.bmj.com/site/about/guidelines.xhtml

INTRODUCTION

Person-Centred Outcome Measures in Palliative Care

Palliative care aims to alleviate suffering due to progressive, life-limiting medical conditions and improve quality of life through the adoption of a holistic, person-centred, and multidisciplinary approach that is delivered across multiple settings (in-patient, outpatient, hospital, and community).[1] It is projected that by 2060, the global need for palliative care will double (from 26 million to 48 million people).[2] It is important, therefore, that we are able to clearly demonstrate the quality of palliative care services by showing if and how they are able to improve a person's symptoms, and sustain or improve wellbeing and functional ability. To do this, focusing on outcomes is essential.

In a healthcare context, an outcome refers to 'the change in a patient's current and future health status that can be attributed to preceding healthcare'.[3] Within palliative care, measuring outcomes is important because they allow us to evaluate whether the care that is given to patients and their families makes a difference to their quality of life. [4] In capturing this information, Person-Centred Outcome Measures (PCOMs) are considered the 'gold standard' for outcome measurement in palliative care. [5, 6] These are standard, validated questionnaires – usually filled in by patients themselves - that provide healthcare professionals with important information on a person's own perception of their health status and well-being. [7] Moreover, because many patients with life-limiting illnesses may have impaired cognition and/or be too unwell to fill out PCOMs on their own accord, [8] PCOMs may sometimes be completed using proxy-reported ratings (e.g., by healthcare professionals and/or a patient's family member).

Current evidence demonstrates the value of PCOMs in palliative care in helping to identify (often unrecognised), continually monitor, and take direct clinical action to address, a

For peer review only - http://bmjopen.bmj.com/site/about/guidelines.xhtml

person's needs/symptoms; facilitate communication between patients and clinicians; and improve outcomes. [4, 7, 9] This is through using PCOMs at individual/patient (e.g., for clinical assessment), team (e.g., focusing patient reviews and workload planning), service (e.g., service evaluation/development and making business cases), and population (e.g., benchmarking) levels of practice. [10] For these reasons, the European Association for Palliative Care Task Force on Outcome Measurement recommended that PCOMs should be firmly embedded into routine clinical practice across all settings in which palliative care is delivered. [6]

Despite the evidence underpinning the value of using PCOMs, they are used inconsistently in clinical practice, if at all. Reasons for this include issues such as time constraints, lack of training and knowledge, tools being perceived as burdensome, negative attitudes towards outcome measures, inefficient electronic systems, top-down approaches to implementation, and fear of added work. [5, 11-13] In addressing these challenges and facilitating the implementation of PCOMs into everyday clinical practice, various studies have provided tables of recommendations to consider before and during implementation. [5, 12, 13] These have recommended focusing on individual, management, and organisational issues during the preparation and roll-out stages of implementation. To date, however, there has been no attempt to develop these into, and subsequently evaluate, an implementation strategy that may be applied across all palliative care settings.

The RESOLVE project

The RESOLVE project is a multi-site implementation and research programme aimed at implementing PCOMs into routine clinical practice in 11 specialist palliative care services across Yorkshire, England. These services vary in size, funding source (e.g., charitable/NHS), location (e.g., urban/semi-rural/rural), and span hospice inpatient, outpatient/day therapy, and home-based/community settings. As part of this study, we conducted an exploratory

For peer review only - http://bmjopen.bmj.com/site/about/guidelines.xhtml

qualitative study in which semi-structured interviews were used to understand the processes and causal mechanisms that underpin the successful implementation of PCOMs into routine practice. [13] Building on insights from this study about implementation and our collaborative work with sites, this study protocol describes a process evaluation of the complex intervention – named 'The RESOLVE PCOM Implementation Strategy' – aimed at facilitating the integration of PCOMs into routine practice. It is important to evaluate these types of interventions to understand what does (and does not work) and in what contexts.

Process evaluations are useful in understanding the ways in which complex interventions work because they provide insight into the causal mechanisms and contextual factors at play during implementation. [14] Currently, however, no process evaluations have been conducted that explore the impact of implementation strategies aimed to promote the uptake and sustained use of PCOMs within palliative care contexts.

Therefore, we aim to conduct a process evaluation that explores the mechanisms through which The RESOLVE PCOM Implementation Strategy does, or does not, support the implementation of PCOMs in routine palliative care practice. We aim to achieve this by answering three research questions:

1. Does The RESOLVE PCOM implementation strategy enhance the uptake and routine use of PCOMs?
2. Does The RESOLVE PCOM implementation strategy enhance health care professionals' individual and collective understanding of, skills in using, and ability to build organisational practices around, PCOMs?
3. If The RESOLVE PCOM implementation strategy is effective, through which mechanisms are its intended effects achieved?

For peer review only - http://bmjopen.bmj.com/site/about/guidelines.xhtml

METHODS AND ANALYSIS

Study design

A multistrand, mixed methods, longitudinal process evaluation informed by The Medical Research Council guidance. [15] To do this, we will adopt a systematic approach in which we provide clear descriptions of, and collect relevant data pertaining to: (i) our complex intervention; (ii) the implementation process; (iii) mechanisms; (iv) context; and (v) outcomes/results (see Table 1). This will be achieved through three strands of work. An overview these can be seen in Table 2.

Core PCOMs set

The three outcome measures we aim to implement are a nationally-recognised and validated core set of PCOMs for specialist palliative care. [16] These measures are based on what patients and their families prioritise as important in advanced illness and have been endorsed by NHS England and Public Health England. They include the palliative Phase of Illness, [17] Integrated Palliative care Outcomes Scale (IPOS), [18, 19] and Australia-modified Karnofsky Performance Status (AKPS). [20] More information about each of these measures can be seen in Table 3.

The implementation of these PCOMs is not simply about increased use, but the wrap-around work that is needed for the effective and appropriate use of these measures to improve care. This includes: organizational and team recognition of why using measures is important, using the right measures at the right time, ensuring follow-up assessments using the measures, getting all teams/team members on board, having efficient feedback systems in place, having all members of the team understanding the purpose of PCOMs, and embedding PCOMs into the 'cultural fabric' of how teams and services operate. [13]

For peer review only - http://bmjopen.bmj.com/site/about/guidelines.xhtml

Table 1: Key components of a process evaluation and how this study has considered these. Grey shaded areas represent key components of a process evaluation according to Medical Research Council guidance.

**Key components of process evaluations**	**Medical Research Council guidance [15]**	**This study**
**Describing the complex intervention and clarifying causal assumptions**	'A clear description of the intended intervention, how it will be implemented, and how it is expected to work, will ideally have been developed before evaluation'	We describe The RESOLVE Person-Centred Outcome Measure (PCOM) Implementation strategy and have drawn on Normalisation Process Theory to explain the causal assumptions through which we expect it to work (see pages 10-12)
**Implementation: what is implemented, and how?**	• 'Implementation process (How delivery is achieved; training, resources etc.)'<br>• 'What is delivered (fidelity, dose, adaptations, reach)'	We have outlined, in detail, the features of our complex interventions using the template for intervention description and replication (TIDieR) checklist (see supplementary file 1). This outlines what each component is, why we are doing it, who is delivering, how much they are delivering, where and when it is being delivered, how it may be tailored/modified, and how well it will be delivered (e.g., fidelity).
**Mechanisms of impacts**	• 'Participant responses to and interactions with the intervention'<br>• 'Mediators'<br>• 'Unexpected pathways and consequences'	In understanding mechanisms of impacts, we are collecting three types of data:<br>- Routinely collected Person-Centred Outcome Measures data (see pages 13-14).<br>- Survey data (see pages 14-17).<br>- Focused interview data (see page 17-19).
**Context**	• 'Contextual factors that shape theories of how the intervention works'<br>• 'Contextual factors that affect (and may be affected by) implementation, intervention mechanisms and outcomes'<br>• 'Causal mechanisms present within the context which act to sustain the status quo, or potentiate effects'	Analysis of focused interview data which provide insight into the contextual factors and mechanisms (e.g., settings of care or organisational characteristics) that affect (and are affected by) the implementation process. (see page 19).
**Outcomes**	Analysis of process data, and integration of process	We will combine results/analysis from quantitative and

For peer review only - http://bmjopen.bmj.com/site/about/guidelines.xhtml

	and outcome data	qualtiative analyses to produce numerous outputs that answer the research questions of this study. (see page 19-20).

For peer review only

Table 2: An overview of the research design for this study

Strand	Data collection method	Data collection timepoints	Reason/output
**1**	Routine outcomes	Baseline and follow-up	To map changes in use (i.e., implementation status) of Person-Centred Outcome Measures over time **(Research Question 1)**
**2**	Survey	Initial piloting phase and then at baseline and follow-up	To determine how changes in Person-Centred Outcome Measures over time (RQ 1) use relates to changes in participants' coherence, engagement, action, and reflexive monitoring of measures **(Research Question 2)**
**3**	Focused interviews	Towards end of project	To determine why? how? context? Of any changes (i.e., mechanisms) **(Research Questions 2&3)**

Table 3: Description of the core set of PCOMs that we aim to implement into routine palliative care practice as part of the RESOLVE project.

Measure	Description	Frequency/timing of collection
palliative Phase of Illness [17]	Palliative Phase of Illness is a measure which describes the urgency of care needs for a person receiving palliative care. It does so by describing four distinct phases of a patient's illness, including: stable, unstable, deteriorating, dying, and deceased. These Phases are measured through determining the care needs of a patient and/or their family and provide a clinical indication of a patient's Phase of Illness which can be used to inform care planning.	**Inpatients (hospice)**<br>- Daily<br><br>**Community**<br>- Each contact
Integrated Palliative care Outcomes Scale (IPOS) [18, 19]	A holistic, well-validated, and global measure of symptom burden that uses 10 questions (scored on a 0-4 Likert type scale) to assess the most important symptoms and concerns of patients affected by life-limiting illnesses across physical, psychological, social, and existential domains of well-being. There are two forms of IPOS; patient-IPOS (where patients complete the questionnaire as a self-report) and staff-IPOS (a proxy version which is completed by staff).	**Inpatients (hospice)**<br>- Initial assessment<br>- Change in Phase of Illness<br>- End of episode<br><br>**Community**<br>- Each contact
Australia-modified Karnofsky Performance Status (AKPS) [20]	Assesses a patient' overall performance/functional status across 3 dimensions: activity, work, and self-care. Healthcare professionals use their observations of patients' ability to perform everyday tasks and scores them at 10% increments between 0% (i.e., the patient is dead) and 100% (i.e., no complaints or evidence of disease).	

For peer review only

For peer review only - http://bmjopen.bmj.com/site/about/guidelines.xhtml

Description and causal assumptions of the RESOLVE PCOM implementation strategy

Description of complex intervention

Whilst all participating sites collect PCOMs in some form, their use is variable. For example, some use all three measures but not consistently across settings, others may use certain measures consistently in some settings but not others, whilst some are seeking to introduce new measures that they do not currently collect. In facilitating the implementation of PCOMs into routine practice in these instances, we have worked collaboratively with specialist palliative care services and health professionals to develop 'The RESOLVE PCOM Implementation Strategy'. This represents a complex intervention in that it 'comprise[s] [of] multiple interacting components' (see Table 4 for a detailed breakdown of each component).[15] The first two of these are general elements that are mostly pre-specified and provided to sites in a standardised manner. The next four are tailored closely to the needs of each site. The final component of this implementation strategy is the role of the quality improvement facilitator which permeates both general and site-specific implementation strategies.

All elements of this implementation strategy are rooted in an assessment of learning needs at each site, the development of rapport with staff, and a visible response to ongoing feedback. They are intended to empower clinical staff to appropriately use PCOMs, including guidance on ensuring the frequency/timing of collection is suitable for the setting in which they are working (see table 3). This may take the form of sharing formal knowledge about PCOMs and how to use them, practical and technical knowledge about IT systems, and/or experiential knowledge about the use of PCOMs in practice. Where PCOM use is affected by team or organisational issues, quality improvement facilitators can empower clinical staff to address these issues and may also play an advocacy role through liaison with senior clinical staff or management. A more in-depth version of the RESOLVE PCOM implementation

For peer review only - http://bmjopen.bmj.com/site/about/guidelines.xhtml

strategy (mapped against the different components included in the TIDIER checklist [21]) can be found in supplementary file 1.

Table 4: An outline of the main components of the RESOLVE PCOM implementation strategy

Implementation component	Characteristics
**General implementation strategies**	
**1. Development of educational resources**	Providing healthcare professionals with resources (e.g., brief instructional videos and pocket guides/outcomes manual) aiming to enhance their knowledge and skills of what outcome measures are, and how to use the right measures at the right time in clinical practice
**2. Workshop and conference events**	Developing and running national and regional workshop events that aim to address common challenges to implementation and facilitate collaborative learning networks between palliative care services
**Site-specific implementation strategies**	
**3. Determining organisational needs**	A baseline assessment - developed through initial interview data and additional liaison with service leads at each site – aimed at understanding the site-specific challenges to implementing PCOMs so that the intervention can be tailored to each site's needs. This includes understanding whether sites want help with better implementing a measure that is already used, or with implementing new measure(s) across or in specific settings
**4. Formal training**	Face-to-face and online educational sessions/presentations with healthcare professionals at sites in order to address local implementation issues/challenges
**5. I.T. support**	Providing informational, practical, and technical support to ensure that each site has the analytic capacity to input outcomes data into, and extract it back out of, electronic systems
**6. Reporting and feedback**	Providing sites with tailored, service-level reports and feedback of their outcomes data to motivate continued use
**Running through general and site-specific implementation strategy**	
**7. Quality improvement facilitator**	Working directly with sites by local site liaison, championing the implementation of PCOMs into practice, identifying and responding to local challenges/needs, keeping PCOMs on the agenda, and acting as a direct link between the research team and sites. An important aspect of this role is to make judicious use of the implementation strategy elements, rather than necessarily deliver the strategy as a whole

Theoretical approach and causal assumptions

As part of their guidance on process evaluations, Moore et al. [15] recommend drawing on theory to propose causal assumptions underpinning how and why The RESOLVE PCOM Implementation Strategy is expected to work. To this end, the components of our

For peer review only - http://bmjopen.bmj.com/site/about/guidelines.xhtml

implementation strategy are theoretically informed by Normalisation Process Theory (NPT).[22, 23] The reason behind selecting NPT is because there is evidence of its effectiveness and value in implementing complex interventions within healthcare settings.[23]

NPT is a type of implementation theory that describes the different types of 'work' that individuals and organisations do in order to 'normalise' complex interventions into clinical practice (i.e., such deep embedment that they become 'invisible'). It does so through proposing four inter-related constructs:

Coherence: The 'sense-making work' that individuals and teams do in order to understand PCOMs.

Cognitive participation: The 'relational work' that occurs in legitimising PCOMs and building everyday clinical practice around using them.

Collective action: The 'operational work' that is performed in order to use PCOMs, including the skills-sets of a workforce and the organisational resources in place that enable the use of PCOMs.

Reflexive monitoring: The 'appraisal work' through which people assess and evaluate the relative value of PCOMs.[22, 24]

We therefore propose that the RESOLVE PCOM implementation strategy will facilitate the implementation of PCOMs into routine practice in four ways: (1) by enhancing professionals' understanding and awareness of how and why to use PCOMs (i.e., improving coherence); (2) by creating an organisational culture where PCOMs are valued and seen as a legitimate way to improve patient care (i.e., enhancing cognitive participation); (3) by ensuring that systems and resources are in place that allows healthcare professionals to easily and meaningfully use PCOMs in everyday practice (i.e., facilitating collective action); and (4) by providing

For peer review only - http://bmjopen.bmj.com/site/about/guidelines.xhtml

meaningful feedback on PCOMs data as to motivate people to continue using them (i.e., enhancing reflexive monitoring).

Patient and public involvement

We support the UK Standards for Public Involvement in research as outlined by the National Institute of Health Research. [25] We have already conducted patient public involvement for strand one of this project (the collection and use of routine outcomes data) in which we consulted with a diverse group of patients about implementation. Furthermore, the RESOLVE project has recruited a lay representative who attends regular project meetings and will be involved in the discussions regarding the interpretation and dissemination of study findings throughout the duration of the project.

Strand one: Mapping change through routinely collected outcomes data

Sampling and recruitment

In recruiting to this study, we will send an invitation letter and participant information sheet to local collaborators at each site. Should sites wish to participate, they will be required to sign and return the study invitation letter. We expect all 11 RESOLVE-affiliated sites to participate in this project.

Data collection

The RESOLVE team have already been working with sites in implementing the palliative Phase of Illness, Integrated Palliative care Outcomes Scales (IPOS), and Australia-modified Karnofsky Performance Status (AKPS) into routine clinical practice. Sites will be required to submit pseudonymised data related to these PCOMs to the RESOLVE team at baseline and follow-up (between 10-12 months later). A more detailed description of the data specification can be found in supplementary file 2.

For peer review only - http://bmjopen.bmj.com/site/about/guidelines.xhtml

Data Analysis

In order to map whether or not the uptake and routine use of PCOMs changes over time, we will use descriptive and analytical statistics to report the levels of missing data at baseline and follow-up (missing complete measures, and missing items).

For each site at baseline, we will assess the missing outcome data at three timepoints: a) start of episode of care, b) first change of Phase of Illness, and c) end of episode of care. To highlight the change in routine use of PCOMs, we will compare the missing outcome data between baseline and follow-up at these 3 timepoints. We will assess missing data on the level of these subscales: physical symptoms (10 items), psychological/emotional symptoms (4 items), and communication/practical issues (3 items). We defined missing outcome data in physical symptoms as 4 or more items missing data out of the 10 items at one point of time. For emotional and communication subscales, missing outcome data was defined as missing at least one item in any of them. We will also compare between the population samples at baseline and follow-up to investigate the comparability of both groups. The variables that we will use to do this will be gender, age, primary diagnosis, and whether a patient lives alone. We will then use t-tests to compare continuous data (e.g., age) with mean and SD, and chi-square to compare categorical data (e.g., gender). All statistical analyses will be conducted at alpha value of 0.05 two-sided level of significance.

Strand two: Online survey data

Conducting survey research will help us to understand whether The RESOLVE PCOM Implementation Strategy has enhanced health care professionals' understandings of, skills in using, and ability to build organisational practices around, PCOMs. The design of the survey strand of this project is informed by Kelley et al.'s [26] guidance on good practice on the conduct and reporting of survey research.

For peer review only - http://bmjopen.bmj.com/site/about/guidelines.xhtml

Sampling and recruitment

We estimate that, when combined, the number of staff across all of the participating services equates to approximately 550 participants. Whilst we aim to distribute this survey to all of these potential participants, we recognise the challenges to online surveys (e.g., participants interests/attitudes to research, communication, structure and length of survey etc.). [27] Therefore, of this number, due to the positive relationships that we have fostered with sites throughout this project, we expect between a 50%-60% response rate. Thus, for the survey part of this research, we estimate between 275-330 participants.

We will use a purposive maximum variation sampling technique [28] as a guide for recruitment, and sample across the following purposive criteria: role, seniority, experience, and settings of care (inpatient, outpatient/day therapy, home-based/community). Through recruiting in this way, we aim to sample an accurate cross-section of the different settings in which PCOMs are used, and the different types of staff members who use them within these settings. This will translate into recruiting staff at different levels/seniority (e.g., those at a medical, senior staff, and junior level) and of differing roles (e.g., doctors, nurses, healthcare assistants, educators, allied healthcare professionals).

To recruit participants, local collaborators at each site will be asked to disseminate an online survey link to healthcare professionals within their organisations. The introductory part of the survey will include the relevant participant information that explains what the purpose of the survey is, why they have been selected, and how data will be used. It will also state that by completing and submitting the survey that they are providing consent to participate in this study. To optimise recruitment and survey completion, we will offer participating sites the incentive of a textbook and/or journal subscription for the organisation for one year.

For peer review only - http://bmjopen.bmj.com/site/about/guidelines.xhtml

Data collection

In keeping with the theoretical framework of this project, we will collect survey data using an adapted version of the NPT survey (The Normalisation MeAsure Development questionnaire (NoMAD). This is a 23-item structured questionnaire that has been developed by the authors of NPT and measures the dynamic processes that underpin the implementation of PCOMs into clinical practice from the perspectives of healthcare professionals directly involved in their use. The NoMAD measure has been found to be an acceptable and psychometrically strong measure which reports good face validity, construct validity, and internal consistency. [29]

Our version of the NoMAD survey consists of three parts which comprise of a combination of closed- and open-ended questions. Part A asks participants to provide demographic information about themselves. Part B asks short questions on what PCOMs participants use and how they use them in everyday practice. Part C is split into four sections, each of which corresponds and asks questions in relation to the different constructs of NPT (coherence, cognitive participation, collective action, reflexive monitoring). This survey can be seen in more detail in supplementary file 3.

We will host our survey online using RedCap software in which participants will be sent a link through which they are able to access and complete the survey. The acceptability of this method, alongside the intelligibility of survey questions, will be piloted on a select number of participants to ensure that they understand the questions being asked and are easily able to complete the online form. Following this, we will make any necessary minor amendments to the NoMAD survey and then administer this to participants at baseline and then at follow-up (10-12 months after baseline collection).

Data analysis

For peer review only

We will conduct four analyses of survey data:

i. Response rate by capturing the number of participants who completed the NoMAD survey compared to the proportion of those who were approached.

ii. Descriptive statistics to describe the general characteristics of healthcare staff who participated in the survey.

iii. Comparison between respondent answers at baseline and follow-up using statistics tests such as paired t-test and McNemar test (when needed) to indicate any changes in PCOMs usage and understanding. All statistical analyses will be conducted at alpha value of 0.05 two-sided level of significance. We recognise that there are numerous potential limitations/challenges of conducting follow-up surveys 12 months after baseline. These include losing participants to follow-up, withdrawal, staff turnover, changed positions, each of which may lead to decrease the sample size at follow-up time. Thus, we decided if any group lost more than 70% of its participants, we will recruit other health staff from the site (who did not participate at baseline) to complete our follow-up sample. Therefore, we will change the analysis plan and treat both groups two independent samples.

iv. Free-text survey responses will be deductively thematically analysed using NPT constructs (coherence, cognitive participation, collective action, reflexive monitoring) as a framework, whilst also allowing for inductive thematic analysis if responses do not fit within the framework.

Strand three: Focused interview data

For strand three, we will collect focused interview data. The collection of qualitative interview data will be able to provide further insight into research questions two and three by providing rich and insightful data on the processes, mechanisms, and contextual factors that

For peer review only - http://bmjopen.bmj.com/site/about/guidelines.xhtml

underpin the ways in which The RESOLVE PCOM Implementation Strategy does, or does not, work to implement PCOMs into routine practice. [30]

Sampling and recruitment

For each site, depending on organisational size, we will recruit between two-five participants. To do this, we will use purposive sampling [28] (based on roles, disciplines, and experience) as a guide to recruit staff who are directly involved in the implementation of PCOMs across different settings, and able to offer a distinct insight/experience into the issues identified from the survey about each of the NPT constructs. Recruitment will be achieved through working with service leads at each site to approach the relevant participants for interview in this study. Participants will then be approached by their service leads with a participant information sheet. Willing participants will then be required to provide informed consent prior to participation.

Data collection

We will conduct a single focused interview with each participant towards the end of the project (i.e., when we finish our implementation work). These interviews will be used as an opportunity to generate rich insight and details of the processes, mechanisms, and contextual factors that underpin how The RESOLVE PCOM Implementation Strategy does, or does not, work to facilitate the implementation of PCOMs into routine palliative care practice. The interview guide (see supplementary file 4) will consist of a series of questions that relate to the different components of NPT (coherence, cognitive participation, collective action, reflexive monitoring) and will present participants with an opportunity to reflect on how their use of PCOMs has changed over time, and how The RESOLVE PCOM Implementation Strategy has impacted this. In mitigating for the COVID-19 pandemic, these interviews will

For peer review only - http://bmjopen.bmj.com/site/about/guidelines.xhtml

take place online (on whichever platform is most convenient for services) at a time that works for them.

Data analysis

Data will be analysed in NVivo using a thematic framework approach. [31] NPT constructs (coherence, cognitive participation, collective action, and reflexive monitoring) will provide a theoretical framework that will be applied deductively to interview transcripts, whilst also allowing for inductive thematic analysis of responses and outliers that do not fit within the framework. This will be through conducting the following interconnected steps: (1) transcription; (2) familiarisation; (3) coding; (4) developing an analytic framework based on NPT; (5) indexing this framework to transcripts; (6) charting data into a framework matrix (and then performing between- and within-case analyses for each theme); and (7) interpreting the data by using theory.

Outputs: Integration of datasets

Each strand of this project will produce different outputs. In strand one, we will use routine outcomes data to produce a traffic light system in which green represents that PCOMs are used appropriately (e.g., in line with the timings/frequencies of collections outlined in table 3), amber that PCOMs are used but inconsistently (e.g., they are used but not in line with the timings/frequencies of collection in table 3), and red that PCOMs are not being used at all. At a glance, this will provide an objective indicator of whether there has been a change (from baseline to follow-up) in the uptake and use of each PCOM across settings, thus enabling us to determine whether the RESOLVE implementation strategy has been successful in implementing PCOMs into routine palliative care practice.

For strand two, we will produce outputs for NoMAD survey data in the form of spider plot graphs. By calculating how mean changes in each NPT component changes from

For peer review only - http://bmjopen.bmj.com/site/about/guidelines.xhtml

baseline to follow-up, this data will be able to complement our analyses of routine outcomes data by seeing if changes in use of PCOMs status coincides with changes in components of NPT.

For strand three, data from interviews will complement routine outcomes and NoMAD survey data by illuminating the mechanisms as to how and why the use of PCOMs have (or have not) changed over time. It will also be able to provide important information about the contextual/organisational factors that impacted this process.

Figure 1 demonstrates how the integration of outputs produced from the analysis of quantitative and qualitative data may look. It will provide a snapshot that will be able to demonstrate the effectiveness The RESOLVE Implementation Strategy at facilitating the implementation of PCOMs into routine palliative care practice. It will also provide us with explanations as to how, why, and in what contexts our intervention works/needs refining so that it can be tailored for particular organisational contexts and/or settings of care.

[INSERT FIGURE 1 HERE]

For peer review only

For peer review only - http://bmjopen.bmj.com/site/about/guidelines.xhtml

ETHICS AND DISSEMINATION

Ethical approval for this project was obtained on 16th September 2020 from the East of England - Cambridge Central NHS Research Ethics Committee [ref: 20/EE/0188]. Ethical approval for strands 2 and 3 of this study was obtained on 18th January 2021 from Hull York Medical School ethics committee [21 05].

We will publish the findings of this study in peer reviewed journals and abstracts will be submitted to national and international conferences. We will also disseminate this work to patients, healthcare professionals, academics, and anybody else who is interested in this work through various non-traditional routes. These will include:

- Producing lay briefs of research findings that we will disseminate to patients and service users through our Palliative Daycare Patient Group and the PPIE network at the University of Hull.
- Producing policy briefs of research findings for policymakers and commissioners
- Sharing the results of research and study newsletters on the RESOLVE and Wolfson Palliative Care Research Centre websites and sharing them on social media.
- Delivering educational workshops to clinicians in order to feedback site-specific findings on what does and does not work with regards to implementing PCOMs within their service
- Utilise the Hospice UK's ECHO Network for Outcomes - monthly national interactive webinars to disseminate the results to clinicians, allied health-care professionals, and others who have an interest in palliative care.

Figure 1 caption: Example of how outputs will be integrated after analysis.

For peer review only - http://bmjopen.bmj.com/site/about/guidelines.xhtml

Author contributions: Conceptualisation and design – AB, AK, MP, and FEM. Development of analysis plan – AB, AK, and FEM. Leading the writing process and drafting the original article – AB. Critically reviewed article for important content – AB, MP, MS, AK, KS, JB, MJ and FEM. All authors take responsibility for the protocol and approved the final version of this paper.

Funding statement: This work was supported by Yorkshire Cancer Research, grant number [L412]. Professor Fliss Murtagh is a National Institute for Health Research (NIHR) Senior Investigator. The views expressed in this article are those of the author(s) and not necessarily those of the NIHR, or the UK Department of Health and Social Care.

Competing interest's statement: The author(s) declared no potential conflicts of interest with respect to the research, authorship, and/or publication of this article.

References

For peer review only - http://bmjopen.bmj.com/site/about/guidelines.xhtml

1. Twycross RG. *Introducing palliative care*. Radcliffe Publishing, 2003.
2. Sleeman KE, de Brito M, Etkind S, et al. The escalating global burden of serious health-related suffering: projections to 2060 by world regions, age groups, and health conditions. *The Lancet Global Health* 2019; 7: e883-e892. DOI: 10.1016/S2214-109X(19)30172-X.
3. Donabedian A. *Explorations in quality assessment and monitoring*. Ann Arbor, MI: Health Administration Press, 1980.
4. Dudgeon D. The impact of measuring patient-reported outcome measures on quality of and access to palliative care. *Journal of palliative medicine* 2018; 21: S-76-S-80.
5. Antunes B, Harding R, Higginson IJ, et al. Implementing patient-reported outcome measures in palliative care clinical practice: a systematic review of facilitators and barriers. *Palliative medicine* 2014; 28: 158-175.
6. Bausewein C, Daveson BA, Currow DC, et al. EAPC White Paper on outcome measurement in palliative care: Improving practice, attaining outcomes and delivering quality services– Recommendations from the European Association for Palliative Care (EAPC) Task Force on Outcome Measurement. *Palliative medicine* 2016; 30: 6-22.
7. Etkind SN, Daveson BA, Kwok W, et al. Capture, transfer, and feedback of patient-centered outcomes data in palliative care populations: does it make a difference? A systematic review. *Journal of pain and symptom management* 2015; 49: 611-624.
8. Hill N. Use of quality-of-life scores in care planning in a hospice setting: a comparative study. *International journal of palliative nursing* 2002; 8: 540-547.
9. Currow DC, Allingham S, Yates P, et al. Improving national hospice/palliative care service symptom outcomes systematically through point-of-care data collection, structured feedback and benchmarking. *Supportive Care in Cancer* 2015; 23: 307-315.
10. Greenhalgh J. The applications of PROs in clinical practice: what are they, do they work, and why? *Qual Life Res* 2009; 18: 115-123. 2008/12/24. DOI: 10.1007/s11136-008-9430-6.
11. Dunckley M, Aspinal F, Addington-Hall JM, et al. A research study to identify facilitators and barriers to outcome measure implementation. *International journal of palliative nursing* 2005; 11: 218-225.
12. Pinto C, Bristowe K, Witt J, et al. Perspectives of patients, family caregivers and health professionals on the use of outcome measures in palliative care and lessons for implementation: a multi-method qualitative study. *Annals of palliative medicine* 2018; 7: S137.
13. Bradshaw A, Santarelli M, Mulderrig M, et al. Implementing person-centred outcome measures in palliative care: An exploratory qualitative study using Normalisation Process Theory to understand processes and context. *Palliative Medicine*; 0: 0269216320972049. DOI: 10.1177/0269216320972049.
14. Craig P, Dieppe P, Macintyre S, et al. Developing and evaluating complex interventions: the new Medical Research Council guidance. *BMJ* 2008; 337: a1655. DOI: 10.1136/bmj.a1655.
15. Moore GF, Audrey S, Barker M, et al. Process evaluation of complex interventions: Medical Research Council guidance. *BMJ : British Medical Journal* 2015; 350: h1258. DOI: 10.1136/bmj.h1258.
16. Witt J, Murtagh F, de Wolf-Linder S, et al. Introducing the Outcome Assessment and Complexity Collaborative (OACC) Suite of Measures-A Brief Introduction. *Kings College London* 2014.
17. Masso M, Allingham SF, Banfield M, et al. Palliative care phase: inter-rater reliability and acceptability in a national study. *Palliative medicine* 2015; 29: 22-30.
18. Hearn J and Higginson I. Development and validation of a core outcome measure for palliative care: the palliative care outcome scale. Palliative Care Core Audit Project Advisory Group. *BMJ Quality & Safety* 1999; 8: 219-227.
19. Murtagh FE, Ramsenthaler C, Firth A, et al. A brief, patient- and proxy-reported outcome measure in advanced illness: Validity, reliability and responsiveness of the Integrated Palliative care Outcome Scale (IPOS). *Palliative medicine* 2019; 33: 1045-1057. 2019/06/12. DOI: 10.1177/0269216319854264.

For peer review only - http://bmjopen.bmj.com/site/about/guidelines.xhtml

20. Abernethy AP, Shelby-James T, Fazekas BS, et al. The Australia-modified Karnofsky Performance Status (AKPS) scale: a revised scale for contemporary palliative care clinical practice [ISRCTN81117481]. *BMC Palliative Care* 2005; 4: 7. DOI: 10.1186/1472-684X-4-7.
21. Hoffmann TC, Glasziou PP, Boutron I, et al. Better reporting of interventions: template for intervention description and replication (TIDieR) checklist and guide. *BMJ : British Medical Journal* 2014; 348: g1687. DOI: 10.1136/bmj.g1687.
22. May CR, Mair F, Finch T, et al. Development of a theory of implementation and integration: Normalization Process Theory. *Implementation Science* 2009; 4: 29.
23. May CR, Cummings A, Girling M, et al. Using Normalization Process Theory in feasibility studies and process evaluations of complex healthcare interventions: a systematic review. *Implementation Science* 2018; 13: 80.
24. Murray E, Treweek S, Pope C, et al. Normalisation process theory: a framework for developing, evaluating and implementing complex interventions. *BMC medicine* 2010; 8: 63.
25. Research NIoH. UK standards for public involvement: Better public involvement for better health and social care research, https://www.invo.org.uk/current-work/standards/ (2021).
26. Kelley K, Clark B, Brown V, et al. Good practice in the conduct and reporting of survey research. *International Journal for Quality in Health Care* 2003; 15: 261-266. DOI: 10.1093/intqhc/mzg031.
27. Saleh A and Bista K. Examining Factors Impacting Online Survey Response Rates in Educational Research: Perceptions of Graduate Students. *Online Submission* 2017; 13: 63-74.
28. Etikan I, Musa SA and Alkassim RS. Comparison of convenience sampling and purposive sampling. *American journal of theoretical and applied statistics* 2016; 5: 1-4.
29. Finch TL, Girling M, May CR, et al. Improving the normalization of complex interventions: part 2 - validation of the NoMAD instrument for assessing implementation work based on normalization process theory (NPT). *BMC Medical Research Methodology* 2018; 18: 135. DOI: 10.1186/s12874-018-0591-x.
30. Sparkes AC and Smith B. *Qualitative research methods in sport, exercise & health. From process to product*. London: Routledge, 2014.
31. Ritchie L, Lewis J, Nicholls CM, et al. *Qualitative research practice: A guide for social science students and researchers* 2ed. Los Angeles, CA: Sage, 2013.

For peer review only

For peer review only - http://bmjopen.bmj.com/site/about/guidelines.xhtml

Figure 1: Example of how outputs will be integrated after analysis.

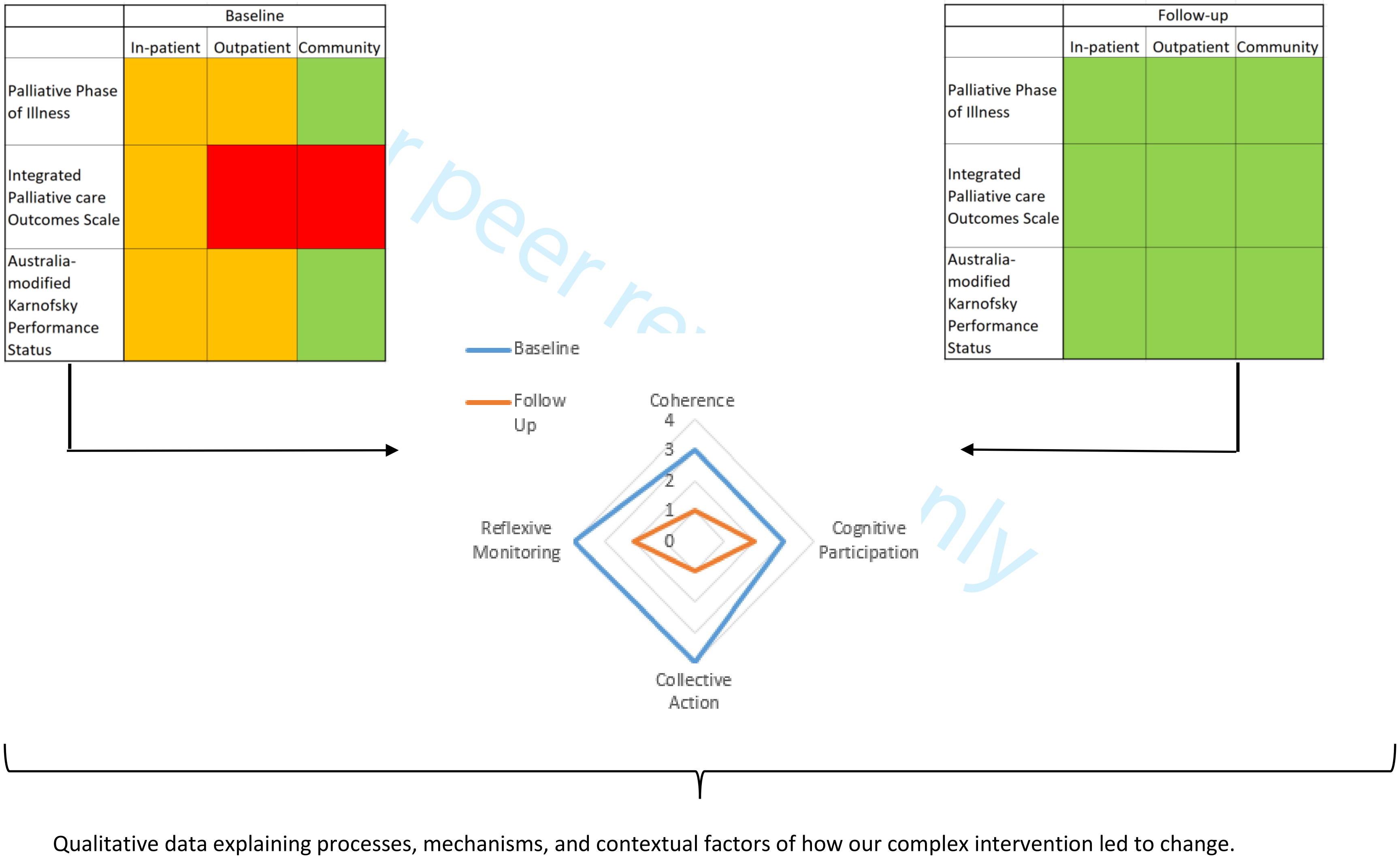

Aspects of implementation

TIDIER Checklist Components

Component Name	Why	What	Who	How	Where	When/How much	Tailoring	Modifications	How well
**General implementation strategies**									
**Development of educational resources**	To enhance healthcare professionals' knowledge of what outcome measures are, their skills in using them, and how they may build practice around their use.	***Materials and procedures:*** Providing healthcare professionals with informational support to facilitate their understanding of what outcome measures are and how/why to use them. This is through the development of teaching and learning materials, including:<br><br>Pocket-guides, outcomes manual, instructional videos, online quizzes	Quality improvement facilitator (Martina), research nurse (Kath), PI (Fliss)	Print-based resources that can be used as an aide to memoir<br><br>Online resources (e.g., quizzes and instructional videos for each measure) available online through the RESOLVE website	Materials are for use during clinical practice and for reflection	Provided to all RESOLVE sites, with additional material being sent upon request	n/a. All sites have access to the same genenral educational resources	To complete retrospectively	To complete retrospectively
**Workshops and conference events**	Workshop and conference events aimed at responding to and discussing the common challenges that participating sites experience with regards to collecting, storing, and using outcome measures	***Materials and procedures:*** Providing informational support through teaching and guidance materials on outcome measures. This is through:<br><br>- Collective workshops and conference training events including representatives from all sites across the project	Entire RESOLVE team	Powerpoint presentations, collaborative group-based activities, question and answer sessions, and expert panel discussions.<br><br>Site representatives present at these events are encouraged to disseminate the knowledge and resources gained from attendance with their colleagues at site.	Conference/workshop events	Twice a year.	The content and agendas of workshop and conference events are derived from the common needs shared across sites participating in RESOLVE	To complete retrospectively	To complete retrospectively
**Site-specific implementation strategies**									
**Determining organisational needs**	Aimed at understanding the site-specific challenges to implementing PCOMs so that we can tailor our intervention according to each site's needs	A baseline assessment - developed through initial interview data and additional liaison with service leads at each site	Andy (research fellow) Martina (QIF)	Interviews, email, telephone communications, site visits	All sites	At the start of tailoring our intervention for each site and revisited regularly throughout implementation	Tailoring intervention components to site-specific needs	To complete retrospectively	To complete retrospectively
**Formal training**	Enhance health care professionals' individual and collective understanding of, skills in using, and ability to build organisational practices around, PCOMs	***Materials and procedures:*** Providing participating sites with informational and organisational support through teaching and guidance on outcome measures. This is through:<br><br>- Site-based training sessions	Quality improvement facilitator (Martina), research nurse (Kath), PI (Fliss)	Working with sites to understand the challenges that they face to using outcome measures in practice. This information is then used to develop formal training sessions (e.g., presentations, case studies etc.) that help in addressing these issues. This is so that we can ensure that participating sites have the knowledge, understanding, and appropriate organisational resources in place to facilitate the consistent collection and use of outcome measures	At sites	Upon request from site	The nature and content of training sessions are tailored to each site's needs and priorities	To complete retrospectively	To complete retrospectively
**I.T. support**	To ensure local I.T. system readiness. That is, making sure that participating services have the analytic capacity and infrastructure in place so that they are able to input the appropriate data items into an electronic system and extract/ submit these to the RESOLVE team	***Materials and procedures:*** Informational, practical and technical support provided to data teams/analysts to assist them with inputting outcomes data into, and extracting it out of, their electronic system	Fliss (PI) and Assem (Data Analyst)	Practical and technical advice via face-to-face site visits, email and telephone	At sites/electronically	Upon request from site	Site specific support for:<br>- Inputting<br>- Viewing and using<br>- Extraction<br>- Submission of routine outcomes data to RESOLVE team	To complete retrospectively	To complete retrospectively
**Reporting and feedback**	Motivating and reinforcing the use of outcome measures through providing sites with meaningful feedback of their outcomes data. This can then be used to demonstrate how what they do as individuals/teams/organisations impacts patient care and outcomes	***Materials and procedures:*** Producing site based reports and graphs on outcomes data through our Registry protocol and feeding this back to each site.	Fliss (PI) and Assem (Data Analyst)	Electronic feedback via email	Electronic	Twice yearly	Site-specific support for:<br>- Producing outcomes reports<br>- Using outcomes data to inform practice at patinet, team, and service levels	To complete retrospectively	To complete retrospectively
**Running through general and site-specific implementation strategies**									
**Quality improvement facilitator (QIF)**	To champion the implementation of PCOMs at sites	Liase and work work sites locally in driving the implementation of PCOMs into practice, identifying and responding to local challenges/needs, keeping PCOMs on the agenda, and acting as a direct link between the research team and sites.	QIF (Martina)	Face-to-face support, email and telephone correspondence	At site	Throughout the RESOLVE implementation strategy	All support from the WIF will be tailored to local site needs and challenges	To complete retrospectively	To complete retrospectively

For peer review only

Version 1.0 08/07/2019

Data item specification

Note: Whilst we plan on collecting data items on dates (e.g., date of referral, first contact data, date ready for care etc.), these will be pseudonymised in Excel by participating sites prior to being submitted to the Registry through the formula below.

Before pseudonymising dates, please make sure that there are no cases from before the date requested. For example, if data is requested from June onwards, disregard any cases with a starting episode date before June.

1. Excel handles dates numerically in the form of 'serial numbers', with each date being represented by a specific serial number. For example, the serial number for the date 24/10/2019 is '42667'. In pseudonymising dates, therefore, each site will use Excel to convert each date (dd/mm/yy) into its serial number through formatting cells into 'general' instead of 'date'.

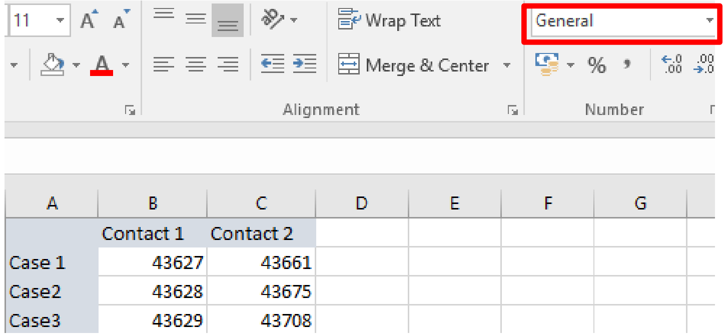

2. Hospices will then select and consistently use a random number between 1-500 (that the research team will not be made aware of) and add this to the serial number before submitting it to the registry.

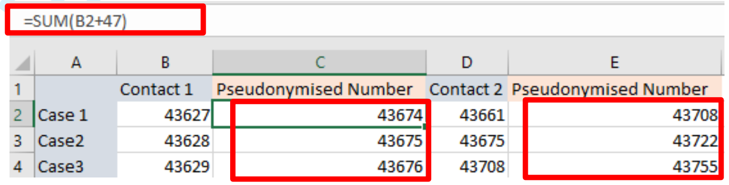

3. This will allow dates to be pseudonymised without the research team being able to trace back to the original date. The numbers that are produced can then be used to determine temporal factors of care that we are interested in(e.g., length of stay, time from referral to first contact) but without receiving dates which may identify participants.

For peer review only

Version 1.0 08/07/2019

Level 1: Patient Details – reported at patient level

No.	Variable Name	Definition	Format/Document	Use
1	Site ID	Unique identifier for each provider	A two letter and two digit code issued by the RESOLVE team (e.g., AA00)	To identify what palliative care provider is submitting data to the registry
2	Pseudonymised Unique Patient ID	A unique and pseudonymised patient identifier within the service's IT system that is used to identify the patient that is being cared by service providers.	This will be created by applying a macro to the NHS number, which provides a unique code which cannot be use to reverse to NHS number	To ensure the positive identification of a patient and that each individual's health records are associated with that individual and no other, while also enabling identification of repeat episodes of care for any one individual.
3	Age	The age of a patient at the start of their first episode of care	Age (in years old)	To determine the age of individual patients for conducting demographic analyses.
4	Gender	The gender of a patient	One of the following:<br>• Male<br>• Female<br>• Other<br>• Unknown	To determine gender of individual patients in a standardized format so that data can be used for related to service utilization, service needs, and epidemiological studies
5	Ethnicity	The ethnicity of the patient	One of the following:<br>• Mixed/multiple ethic groups<br>• Asian/Asian British<br>• Black/African/Caribbean/Black British<br>• Other ethnic group<br>• White	To determine ethnicity of individual patients in a standardized format so that data can be used for related to service utilization, service needs, and epidemiological studies
6	Primary Diagnosis	The primary life-limiting illness responsible for the patient receiving palliative care	Options showed in appendix 1 (ICD10 classification)	To determine a patients primary diagnosis.
7a	Secondary Diagnosis 1	Any secondary conditions / co-morbidities the patient has.	Options showed in appendix 1 (ICD10 classification)	To determine a patients secondary diagnosis.
7b	Secondary Diagnosis 2			
7c	Secondary Diagnosis 3			
8	Lives alone?	Whether or not the patient lives alone.	One of the following options:<br>• Yes	To understand a patients living arrangements for analysis (e.g., impact of living arrangements of cost of care and other outcomes)

For peer review only

Version 1.0 08/07/2019

No.	Variable Name	Definition	Format/Document	Use
			• No<br>• Unknown	
9	Usual Place of Residence	The patient's current main or permanent place of residence	One of the following:<br>• Patient's own home<br>• Other Private Residence (e.g., Relative's/Carers home)<br>• Care Home (residential/nursing facility)<br>• Hospice (inpatient specialist palliative care)<br>• Hospital<br>• Homeless<br>• Traveler<br>• Other	To understand place of care
10	Marital Status	A patient's marital status.	One of the following options:<br>• Single<br>• Married/Civil Partner/Cohabiting<br>• Divorced/Person whose Civil Partnership has been dissolved<br>• Widowed/Surviving Civil Partner<br>• Separated<br>• Not disclosed<br>• Unknown	To understand a patients marital status for analysis

For peer review only

Level 2: Episode Details – reported at episode level

No.	Variable Name	Definition	Format/Document	Use
11	Palliative episode number	The number episode that a patient is in	The corresponding number with a patient's episode (e.g., 1 for their first episode of care, 2 for their second, 3 for their third etc.)	To identify the number episodes a patient has experienced.
12	Referral Date	The date that a service receives a referral to provide palliative care for a patient for this episode	Date of referral (DD/MM/YYYY)	This will serve as a **key benchmarking variable**; the measurement of the time between referral and other variables (e.g., first contact date, date ready for care, episode start date). ***This date will be pseudonymised using the formula outlined at the start of this document.**
13	Primary Reason for referral	The primary reason for referral to specialist palliative care, as identified by person who refers patient.	One of the following options:<br>• Pain<br>• Other symptom control<br>• Emotional/psychological/spiritual support (patient)<br>• Emotional/psychological/spiritual support (family)<br>• Social/financial support (patient)<br>• Social/financial support (family)<br>• Rehabilitation<br>• Discharge planning<br>• Care in last days of life<br>• Respite<br>• Other	For analyses regarding why patients are referred to specialist palliative care services
14	Referral Source	The facility/organization which the patient was referred from for the start of a particular episode.	One of the following:<br>• Hospital – NHS<br>• Hospital – Outpatient<br>• Hospice – Inpatient<br>• Hospice – Day services<br>• Hospice – Outpatient<br>• Community – GP<br>• Community – Hospice at home<br>• Community – Out of hours services	To assist in understanding referral patterns and service planning.

For peer review only - http://bmjopen.bmj.com/site/about/guidelines.xhtml

Version 1.0 08/07/2019

No.	Variable Name	Definition	Format/Document	Use
			• Community – District/Community matron<br>• Self-referral/family-referral<br>• Internal referral within organisation<br>• Other (please state)	
15	First Contact Date	The date the clinical team makes contact with patient to assess needs after referral	Date of first clinical contact (DD/MM/YYYY)	This is a **key benchmarking variable** as it can be used to measure response time. ***This date will be pseudonymised using the formula outlined at the start of this document.**
16	Date Ready for Care	The date the patient is ready and available to receive palliative care. This may be determined through referral or first contact with patient	Date identified that patient is ready to receive palliative care (DD/MM/YYYY)	**Key benchmarking variable** as it is used to identify and account for early referrals (i.e., when a referral is received by a patient is not ready for care). ***This date will be pseudonymised using the formula outlined at the start of this document.**
17	Episode Start Date	The date of the first face-to-face palliative care assessment, using the set of core outcomes listed above. This is when clinical care starts	The date when clinical care starts (DD/MM/YYYY)	This date (along with episode end date) will be used to determine the length of stay for each episode of care. ***This date will be pseudonymised using the formula outlined at the start of this document.**
18	Care Setting	The setting/location where a patient is overnight.	One of the following:<br>• Hospital – Inpatient<br>• Hospital – Outpatient<br>• Hospice – Inpatient<br>• Community – Outpatient<br>• Community - Home visiting<br>• Community – Day care or day service<br>• Community – Nursing/residential home<br>• Community – Patient's/Carer's own home<br>• Community – Community hospital<br>• Other (please state)	To determine where an episode of care takes place
19	Team delivering care	The team delivering care within setting	Provided by the reporting organisation	Each organisation has different 'labels' for their teams (such as different clinics, therapy teams etc), yet may require sub-analysis by team to support effective quality improvement – we will therefore allow each reporting organisation to self-specify this variable
20	Episode end date	The date when a patient moves from one setting of care to another, dies, or	Date of episode end (DD/MM/YYYY)	To use with episode start date to determine the length of care for each episode. ***This date will be pseudonymised using the formula outlined at the**

For peer review only

Version 1.0 08/07/2019

No.	Variable Name	Definition	Format/Document	Use
		is no longer receiving palliative care		**start of this document.**
21	Episode result	The reason that an episode of palliative care ended	One of the following:<br>• Discharged to own home (or relatives/carers home)<br>• Discharged to nursing/residential home<br>• Discharged to hospital<br>• Discharged to NHS hospice<br>• Discharged to non-NHS hospice<br>• Died<br>• Unknown	Describes how the episode ended, determines number of deaths, and discharge locations.
22	Preferred place of death (if relevant)		One of the following:<br>• Nursing/residential home<br>• Home<br>• Hospice<br>• Hospital<br>• Other	
23	Whether patient is now deceased		• Yes<br>• No<br>• Unknown	Reporting may occur some time after episodes of care are completed. Knowing if the patient subsequently died will enable reporting of how early in the trajectory of illness patients are being seen.
24	Date of death		Date of death (DD/MM/YYYY)	This date will be used to determine at what point in the illness trajectory, the episode of care was delivered. ***This date will be pseudonymised using the formula outlined at the start of this document.**
25	Place of death (if relevant)		One of the following:<br>• Nursing/residential home<br>• Home<br>• Hospice<br>• Hospital<br>• Other	

For peer review only

Version 1.0 08/07/2019

Level 3: Phase Details – reported at contact level

No.	Variable Name	Definition	Format/Document	Use
26	Palliative Phase Number	The number phase that a patient has within an episode.	The corresponding number with a patient's phase (e.g., 1 for their first phase of illness, 2 for their second, 3 for their third etc.)	To identify the number phases a patient has experienced within an episode and to work out number of patient contacts.
27	Palliative Phase of Illness	Stage of a patient's illness, assessed at the start of the phase as assessed by Phase of Illness questionnaire (see appendix X)	One of the following:<br>• Stable<br>• Unstable<br>• Deteriorating<br>• Dying<br>• Deceased<br>• Bereavement/post death support<br>• Unknown (see appendix X for phase definitions)	To identify the stage of a patient's illness
28	Phase Start Date	The date each new phase of illness begins	Phase start date (DD/MM/YYYY)	When used with phase end date, can tell us the length of a phase of illness. ***This date will be pseudonymised using the formula outlined at the start of this document.**
29	Phase End Date	The date each phase of illness ends	Phase end date (DD/MM/YYYY)	When used with phase start date, can tell us the length of a phase of illness. ***This date will be pseudonymised using the formula outlined at the start of this document.**
30	Is this the End of a Phase?	Whether the patient has reached the end of a phase of illness.	One of the following:<br>• Yes<br>• No	To clarify the end of a phase of illness
31	Has the Phase Changed?	To highlight whether a patient has undergone a change in their phase of illness.	One of the following:<br>• Start Phase<br>• Continuing Phase<br>• End Phase	To cross check with phase of illness assessments
32	Contact Date	The date a health-care professional has clinical contact with patient.	DD/MM/YYYY.	To align contact dates – and clinical data collected at these - with changes in episodes and phases. ***This date will be pseudonymised using the formula outlined at the start of this document.**
33	Australia-Modified Karnofsky Performance	An assessment a patient's functional status at the beginning of a phase of illness	Functional status, as measured by AKPS ranging from 0-100% in 10% increments, (where 100% is fully functioning and 10% is unarousable). See	**Key benchmarking variable**. An analysis of functional status at phase start

For peer review only

Version 1.0 08/07/2019

No.	Variable Name	Definition	Format/Document	Use
	Scale (AKPS) Score		appendix X for scale	
34	Integrated Patient Outcomes Scale (IPOS) at	An assessment of a patient's symptoms and other concerns at phase start (see items between numbers X-X)	Inputting data from each item of IPOS (see appendix X)	**A key benchmarking variable**. A measure of a patient's perceptions of their symptoms at the start of a phase of illness
35	Barthel Activities of Daily Living	To assess patient's activities of daily living at phase start	Inputting data from each item of BARTHEL (see appendix X)	
36	Views on Care (VOC)	To assess a patient's ratings of their quality of life, overall well-being, and their view of the impact of the service on their problem(s) at the start of a phase of illness	Inputting data from each item of VOC (see appendix X)	To measure a patients views on care

For peer review only

Version 1.0 08/07/2019

Appendix 1: ICD10 Classifications

No.	RESOLVE Diagnoses Groups
1	Cancer - Cancer of bone, skin, mesothelial and soft tissue, thyroid or endocrine [C40-C49 & C73-C75]
2	Cancer - Cancer of breast [C50]
3	Cancer - Cancer of independent multiple sites [C97]
4	Cancer - Cancer of liver, intrahepatic bile ducts, gallbladder [C22-C24]
5	Cancer - Cancer of respiratory & intrathoracic organs, including lung (C30-C39)
6	Cancer - Cancer of the digestive organs, including colon, rectum, stomach, excluding liver, GB, pancreas [C15-C21 & C26]
7	Cancer - Cancer of the eye, brain and other CNS [C69-C72]
8	Cancer - Cancer of the female genital organs [C51-58]
9	Cancer - Cancer of the lip, oral cavity & pharynx [C00-C14]
10	Cancer - Cancer of the male genital organs, including prostate [C60-C63]
11	Cancer - Cancer of the pancreas [C25]
12	Cancer - Cancer of the urinary tract [C64-C68]
13	Cancer - Cancer of unknown primary or other unspecified [C76-C80]
14	Cancer - Lymphoid & haematopoietic cancer [C81-C96]
15	Multiple non-cancer conditions - addition to help with multi-morbidity
16	Non-cancer - All other non-cancer diagnoses
17	Non-cancer - Chronic renal failure [N18]
18	Non-cancer - Chronic respiratory disease [J40-J70]
19	Non-cancer - Dementia including Alzheimer's disease [G30 & F00-F03]
20	Non-cancer - Diabetes mellitus [E10-E14]
21	Non-cancer - Heart failure [I50]
22	Non-cancer - HIV disease/AIDS [B20-B24]
23	Non-cancer - Liver failure, chronic liver disease, other non-malignant liver disease [K70-K77]
24	Non-cancer - Motor neurone disease [G12]
25	Non-cancer - Neurological conditions (excluding MND and Alzheimer's) [G00-G99

For peer review only

Version 1.0

08/07/2019

No.	RESOLVE Diagnoses Groups
	excluding G12 & G30]
26	Non-cancer - Other heart & circulatory conditions excluding heart failure and stroke [I00-I99 excluding I50, I61, I63]
27	Non-cancer - Stroke, infarction or haemorrhagic [I61 & I63]
28	Unknown

For peer review only

IMPLEMENTING PERSON-CENTRED OUTCOMES MEASURES INTO PALLIATIVE CARE

Version 1 (15/12/2020)

Information about this survey

What is this survey about?

This survey is part of a study called 'RESOLVE'. RESOLVE is a research programme aimed at facilitating the implementation of Person-Centred Outcome Measures into routine palliative care. This is so that, as a palliative care community, we ensure regular assessment and monitoring of symptoms and other concerns of those receiving palliative care. As part of our project we have developed an intervention strategy, and this survey is one way in which we are trying to understand if it is effective at facilitating the integration of outcome measures into everyday practice. More information on the RESOLVE project can be found through our website: https://www.hyms.ac.uk/research/research-centres-and-groups/wolfson/resolve.

Why have I been chosen?

You have been selected to complete this survey as you are a staff member at a RESOLVE-affiliated site that has received the RESOLVE implementation strategy. Sites with the best completion rate will receive a voucher for a textbook or one-year journal subscription.

Do you consent to taking part in this survey?

☐ Yes ☐ No

What about my data?

By completing and submitting this survey you are providing consent for your responses to be used as part of the RESOLVE research project. All data is anonymised and confidential and will be stored in accordance with General Data Protection Regulation (GDPR). If you have any questions, please contact the RESOLVE team (resolve@hyms.ac.uk).

Survey Instructions

This survey is split into 3 parts and should take no longer than 15 minutes to complete. Part A asks some brief questions about yourself and your role. Part B includes questions about what outcome measures you use and how you use them to inform your everyday practice. Part C asks more detailed questions about PCOMs.

For peer review only - http://bmjopen.bmj.com/site/about/guidelines.xhtml

Please take the time to decide which answer **best suits your experience for each statement and tick the appropriate box**

Part A: About yourself

- What is your professional role?
  ☐Nurse ☐Doctor ☐Allied health professional ☐Healthcare assistant ☐Chief exec/manager ☐ I.T. or data ☐Other (dropdown box)

- What is the name of your organisation?
  ☐ Dove House Hospice ☐ Marie Curie Bradford Hospice ☐ Wheatfields Hospice ☐ St Gemma's Hospice ☐ St Andrew's Hospice
  ☐ Grimsby Care Plus Group ☐ Kirkwood Hospice ☐ St Luke's Hospice ☐ St Catherine's Hospice
  ☐ City Health Care Partnership Hull ☐ Bradford District Care Trust ☐ York Teaching Hospital NHS Foundation Trust

- What is your age?
  ☐ 18-24 ☐ 25-34 ☐ 35-44 ☐ 45-54 ☐ 55+ ☐ Prefer not to say

- What is your gender?
  ☐ Male ☐ Female ☐ Other ☐ Prefer not to say

- By which ethnicity do you identify?
  ☐ White ☐ Black/African/Caribbean/Black British ☐ Asian/Asian British ☐ Mixed/multiple ethic groups ☐ Other
  ☐ Prefer not to say

- What setting do you work in (tick multiple if you work across settings)?
  ☐ In-patient hospice ☐ Outpatient/day therapy ☐ Community ☐ Hospital

- How long have you worked in palliative care?
  ☐ Less than a year ☐ 1-5 years ☐ 6-10 years ☐ 11-15 years ☐ 15+ years

- From the statements below, please choose an option that best describes ***your main role*** in relation to outcome measures:
  ☐ I am involved in managing or overseeing the implementation and use of outcome measures
  ☐ I am involved in using outcome measures as part of my everyday practice
  ☐ I use outcome measures occasionally as part of my everyday practice
  ☐ I am not using outcome measures as part of my everyday practice

For peer review only

For peer review only - http://bmjopen.bmj.com/site/about/guidelines.xhtml

Part B: What outcome measures do you use?

Outcome measures are short questionnaires that provide healthcare professionals with information on a person's own perception of their health status and well-being. They include palliative Phase of Illness, Integrated Palliative care Outcome Scale (IPOS), and Australia-modified Karnofsky Performance Scale (AKPS). This survey relates to your use of these outcome measures.

- Do you use the following outcome measures?

	At 1st assessment	At follow-up
palliative Phase of Illness	☐ Yes ☐ No	☐ Daily ☐ Weekly ☐ At clinical assessment ☐ Other (open free text response box)

	At 1st assessment	At follow-up
Integrated Palliative care Outcome Scale (IPOS)	☐ Yes ☐ No	☐ Yes ☐ No
Australia-modified Karnofsky Performance Scale (AKPS)	☐ Yes ☐ No	☐ Yes ☐ No

- At what follow up interval do you collect IPOS and AKPS data?
  ☐ At change of palliative Phase of Illness ☐ End of episode of care ☐ Other (open free text response box)

- Do you or your team use these measures to shape the care of individual patients, including in multi-disciplinary team meetings?

- Do you or your team use these measures to shape the services that your team or organisation delivers?

For peer review only

Part C: Detailed questions about outcome measures

These questions are designed to help us find out more about whether people and teams understand what outcome measures are and how they may be used during practice. For each statement please select an answer that best suits your experience.

**Section C1**	Strongly agree	Agree	Neither agree nor disagree	Disagree	Strongly disagree	Not relevant to my role	Don't know
**I can see how outcome measures differs from usual ways of working**	☐	☐	☐	☐	☐	☐	☐
**Palliative care staff in this organisation have a shared understanding of the purpose of outcome measures**	☐	☐	☐	☐	☐	☐	☐
**I understand how using outcome measures affects the nature of my own work**	☐	☐	☐	☐	☐	☐	☐
**I can see the potential value of outcome measures**	☐	☐	☐	☐	☐	☐	☐

- Please feel free to expand on your responses below

These questions are designed to help us find out more about how everyday practice is built around using outcome measures and how you think they relate to your role. For each statement please select an answer that best suits your experience.

**Section C2**	Strongly agree	Agree	Neither agree nor disagree	Disagree	Strongly disagree	Not relevant to my role	Don't know
**There are key people who drive the use of outcome measures and get others involved**	☐	☐	☐	☐	☐	☐	☐
**I believe that using outcome measures is a legitimate part of my role**	☐	☐	☐	☐	☐	☐	☐
**I am open to working with colleagues in new ways to use outcome measures**	☐	☐	☐	☐	☐	☐	☐
**I will continue to support the use of outcome measures**	☐	☐	☐	☐	☐	☐	☐

- Please feel free to expand on your responses below

For peer review only - http://bmjopen.bmj.com/site/about/guidelines.xhtml

These questions are designed to help us better understand the ways in which you experience using outcome measures as part of your role. For each statement please select an answer that best suits your experience.

**Section C3**	Strongly agree	Agree	Neither agree nor disagree	Disagree	Strongly disagree	Not relevant to my role	Don't know
**I can easily integrate outcome measures into existing work**	☐	☐	☐	☐	☐	☐	☐
**Outcome measures disrupt working relationships**	☐	☐	☐	☐	☐	☐	☐
**I have confidence in other people's ability to use outcome measures**	☐	☐	☐	☐	☐	☐	☐
**Work is assigned to those with skills appropriate to using outcome measures**	☐	☐	☐	☐	☐	☐	☐
**Sufficient training is provided to enable palliative care staff to implement and use outcome measures**	☐	☐	☐	☐	☐	☐	☐
**Sufficient resources are available to support outcome measures use**	☐	☐	☐	☐	☐	☐	☐
**Management adequately supports the use of outcome measures**	☐	☐	☐	☐	☐	☐	☐

- Please feel free to expand on your responses below

For peer review only

These questions are designed to help us understand the ways in you appraise the value of outcome measures. For each statement please select an answer that best suits your experience.

**Section C4**	Strongly agree	Agree	Neither agree nor disagree	Disagree	Strongly disagree	Not relevant to my role	Don't know
**I am aware of reports about the effects of outcome measures on patient care**	☐	☐	☐	☐	☐	☐	☐
**The palliative care staff agree that using outcome measures is worthwhile**	☐	☐	☐	☐	☐	☐	☐
**I value the effects that outcome measures have had on my work**	☐	☐	☐	☐	☐	☐	☐
**Feedback about outcome measure data can be used to improve how they are used in future**	☐	☐	☐	☐	☐	☐	☐
**I can modify how I work with outcome measures**	☐	☐	☐	☐	☐	☐	☐

- Please feel free to expand on your responses below

For peer review only

- Are you happy to be contacted to complete this survey again (in around 8-12 months' time)? This is so that we are able to explore the impact of the RESOLVE implementation strategy over time.
  - ☐ Yes (if selected then include dropdown box that asks for email address so that we are able to contact, alongside statement that we will send them a certificate of participation for this survey)
  - ☐ No (if selected then include dropdown box that asks if respondent wants to receive a certification of participation. If they do, prompt for email address)

Thank you very much for completing this survey.

What happens next?

This data will be stored confidentially and analysed as part of the RESOLVE project to understand the ways in which the RESOLVE implementation strategy has (or has not) worked to help implement outcome measures into routine clinical practice. This data will be used as part of reports, publications, and conference presentation. All information included within these outputs will be anonymised and it will not be possible for others to identify you. If you have any questions, please contact the RESOLVE team (resolve@hyms.ac.uk).

For peer review only

Version 1 (15/12/2020)

Introduction to the Interview

Thank you very much for volunteering to be interviewed for the RESOLVE project. The aim of this project is to implement Person-Centred Outcome Measures (PCOMs) into routine palliative care across Yorkshire.

As part of RESOLVE, we have developed an implementation strategy to facilitate the implementation of PCOMs into practice. This strategy was developed based on what health professionals' thought were important challenges that needed to be addressed in order to integrate PCOMs into routine practice. Our strategy includes:

- Educational resources
- Workshop and conference events
- Determining organisational and team needs
- Formal and informal training
- IT support
- Reporting and feedback
- Quality improvement facilitator

The purpose of this discussion is to understand your thoughts and feelings on the extent to which you think this implementation strategy has been effective at enhancing the uptake and routine use of PCOMs in everyday practice. It should take around 30 minutes.

Do you have any questions before we start?

The first few questions are around what measures you currently use and how you use them.

- Are you able to tell me what palliative care outcome measures you use in your everyday practice? (probe for use of the three core measures: palliative Phase of Illness, AKPS, and IPOS)
- When were these measures introduced? (probe for when each of the three core was introduced)
- How do you use these measures in practice? Probe for:
  - Who collects these measures?
  - How often they are collected/timings?

- Which version is used (largely version of IPOS, but also version of Barthel if mentioned)?

- Has your use of outcome measures changed over the last 1-2 years?
  - Collecting?
  - Scoring?
  - Reporting?
  - General understanding of each measure (i.e., what they are, how to use them, their value)?
- Do you think that the way in which you currently use outcome measures is better or worse than previously?
  - What has changed or remained the same?
  - Why?

The next few questions focus on the ways in which you may have used different components of the RESOLVE implementation strategy and how, if at all, you feel as though it may have impacted how you use outcome measures.

- Have you, or your team/service, used any of the RESOLVE implementation strategies
  - Educational resources
  - Workshop and conference events
  - Determining organisational and team needs
  - Formal and informal training
  - IT support
  - Reporting and feedback
  - Quality Improvement Facilitator, or other RESOLVE team members

For peer review only

Version 1 (15/12/2020)

- Can you comment on the extent to which you feel the RESOLVE implementation has been put into practice within your service?
  - Which elements?
  - How much?

Coherence

- What do outcome measures mean to you in relation to your role?
  - Have these views changed over time?
  - Why?
- Do you feel as though the RESOLVE implementation strategy has changed your own (and other colleagues') understandings of what outcome measures are and how to use them?
  - On scoring consistently and confidently?
  - On what they mean (especially Phase of Illness)?
  - Frequency of collection (and selecting the relevant versions)?
- How and why do you think these things?

Cognitive Participation

- Are you able to reflect on the ways in which the RESOLVE implementation strategy may have contributed to building clinical/team practice around outcome measures?
  - Has it influenced communicating with outcome measures in relation to individual patients (i.e., ward rounds, MDT's, handovers etc.)?
  - How?
  - Has it shaped the services that your team or organisation delivers?
  - How?

For peer review only

Version 1 (15/12/2020)

Collective Action

- Thinking back over the last year, has the way in which you use outcome measures (both individually and as part of your team) changed as a result of the RESOLVE implementation strategy?
  - How?
  - What is different now?
  - What parts of the RESOLVE implementation strategy do you feel are responsible for these changes?
- Has the implementation strategy helped to develop your own, and your teams, skills, and confidence in using outcome measure to inform care at a patient/team/service level?
  - What has helped with this?
- Were there any organisational barriers to using outcome measures (individually and as part of your team) that the RESOLVE implementation strategy has helped to address?
  - What were these?
  - How/why did the implementation strategy help?
- Has your confidence to confidently/efficiently input, share, and extract outcomes data within your electronic systems changed?
  - If any, what part of the RESOLVE implementation strategy has been key to this?

Reflexive Monitoring

- Are you able to reflect on the ways in which the RESOLVE implementation strategy has changed your views on how you value outcome measures in everyday practice?

For peer review only

- How?
- Why?

➢ How do you share feedback on outcome measures?

- Do you feel as though the RESOLVE implementation strategy has had an impact on for you/your team/the organisation to do this?
- How/Why?

➢ Are there any aspects of the RESOVLE implementation strategy that you think have contributed/will contribute to sustained use of outcome measures?

- What are these?
- Why?
- How will use be sustained once the RESOLVE project is over?

Thank you for taking part in this interview. Before we finish, do you have any other questions?

For peer review only
